# Worldwide revision of synanthropic silverfish (Insecta: Zygentoma: Lepismatidae) combining morphological and molecular data

**DOI:** 10.1093/jisesa/ieae045

**Published:** 2024-05-04

**Authors:** Rafael Molero-Baltanás, Andrew Mitchell, Miquel Gaju-Ricart, Jairo Robla

**Affiliations:** Department of Zoology, University of Córdoba, Campus de Rabanales, C-1 Building, 3rd Floor, Córdoba 14071, Spain; Australian Museum, 1 William Street, Sydney, NSW 2010, Australia; Department of Zoology, University of Córdoba, Campus de Rabanales, C-1 Building, 3rd Floor, Córdoba 14071, Spain; Department of Conservation Biology, Doñana Biological Station (EBD-CSIC), C/Américo Vespucio 26, Seville 41092, Spain

**Keywords:** biological invasion, DNA barcode, COI sequence, identification key, Lepismatidae phylogeny

## Abstract

Synanthropic silverfish are the best-known and most widely distributed insects of the order Zygentoma. However, there is a great gap in the knowledge and confusion about the geographic distribution and the diagnostic characteristics that allow their identification. In this work, we provide an exhaustive and deep analysis of the most common 9 synanthropic silverfish of the world, combining previously published and newly derived morphological and molecular data. Updated descriptions of *Ctenolepisma calvum* (Ritter, 1910) and *Ctenolepisma* (*Sceletolepisma*) *villosum* (Fabricius, 1775) are included, and morphological remarks, illustrations, and photographs of the remaining synanthropic species are provided to clarify their diagnosis and differentiation among them and from other free-living species. In addition, *Ctenolepisma targionii* (Grassi and Rovelli, 1889) is synonymized with *C. villosum.* A molecular phylogeny is presented based on the COI sequences of all the synanthropic species deposited in BOLD and GenBank, with 15 new sequences provided by this study. This has allowed us to detect and correct a series of identification errors based on the lack of morphological knowledge of several species. Moreover, 2 different lineages of *Ctenolepisma longicaudatum*  Escherich, 1905 have also been detected. To help future studies, we also provide a taxonomic interpretation guide for the most important diagnostic characters of the order Zygentoma, as well as an identification key for all the Synanthropic studied species. Finally, an approximation of the global distribution of synanthropic silverfish is discussed. Several new records indicate that the expansion of these species, generally associated with the transport of goods and people, is still far from over.

## Graphical Abstract

**Graphical Abstract:**
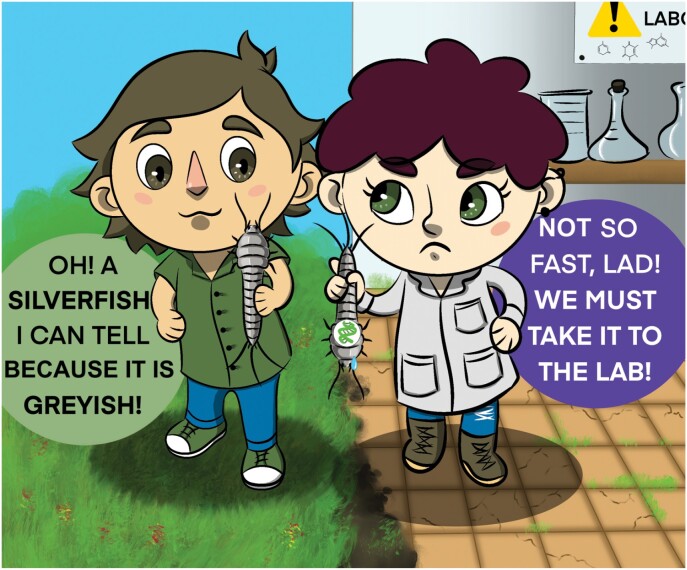

## Introduction

Lepismatidae Latreille is a family of primitive wingless insects, generally known as “silverfish,” that includes about 300 species distributed worldwide, although poorly represented in higher latitudes (Irish 1994). These ancient insects are included in the order Zygentoma Börner. Most Lepismatidae are free-living species that can be found in both natural and seminatural habitats (Smith 2017, Mendes 2018). Some species live in ant or termite nests as parasites or commensals (Robla et al. 2023), and several species can be considered synanthropic, living as human commensals in buildings (Molero-Baltanás et al. 1997, Mendes 2002, Hage et al. 2020, Kulma et al. 2021).

Synanthropic silverfish are among the most studied species of Zygentoma. Most of them have traveled passively with the transit of goods and people throughout the entire world from very ancient times (Baeta-Neves et al. 1956). In fact, many of these species can be considered cosmopolitan, although there is limited information about their distribution in natural habitats, and related to this, the geographical origin of most species is uncertain. Several of these synanthropic silverfish species are considered economic and nuisance pests (Smith 2017) because of their ability to feed on paper, cardboard, rubber, and other carbohydrate-rich materials (Lasker and Giese, 1956). They can damage books, book bindings, documents, and photographs (Querner 2015), which has resulted in their being classified as important pests in museums, archives, or libraries (Querner 2015, Wilke et al. 2018). They can also damage cotton and silk (Mallis et al. 1958), among other natural materials. Recently, they have also been identified as carriers of certain allergens (Panzani and Ariano 2001, Barletta et al. 2007, Boquete et al. 2008), opportunistic bacteria, and other parasites with medical human interest (Lindsay 1939, Kulma et al. 2021). Even with such apparent interest, information about the geographical distribution of many synanthropic silverfish, their natural origin, and their morphological diagnosis is still imprecise.

In recent years, several species have been reported for the first time in some countries, suggesting an increasing tendency in the expansion of these silverfish. These are the cases of *Ctenolepisma longicaudatum*  Escherich (1905) (Goddard et al. 2016, Kulma et al. 2018, Thomsen et al. 2019, Aak et al. 2021), *Ctenolepisma lineatum* (Fabricius, 1775) (Hage et al. 2020), and the lesser-known *Ctenolepisma calvum* (Ritter, 1910) (Kulma et al. 2022, Querner et al. 2022, Shimada et al. 2022, Bednár et al. 2023). The list of synanthropic silverfish also includes species already well known and widely distributed, such as *Lepisma saccharinum*  Linnaeus, 1758, *Acrotelsa collaris* (Fabricius, 1793), *Thermobia domestica* (Packard, 1873), *Thermobia aegyptiaca* (Lucas, 1840), *Ctenolepisma rothschildi* Silvestri, 1907, *Ctenolepisma targionii* (Grassi and Rovelli, 1889), and *Ctenolepisma villosum* (Fabricius, 1775). However, many records have been published without enough taxonomic background to ensure correct identification, and the absence of Zygentoma specialists, updated identification keys, and accurate descriptions using current silverfish taxonomy have favored the spread of many identification errors.

Furthermore, some nonspecialists have described species that have turned out to be synonyms of previously described species. This also leads to the incorrect identification of the genomes and DNA data deposited in GenBank, some of which could be based on misidentified silverfish species. For example, an odd relationship of a previously sequenced *Lepisma saccharinum* was detected by Cucini et al. (2021), suggesting the incorrect identification of the specimens used for molecular data. In addition, citizen science platforms are contributing somewhat to the expansion of these errors through the use of photo-based identifications. Insects belonging to the order Zygentoma generally require microscopic analysis for distinguishing species and even genera (Stach, 1946), so photo-identification is usually discouraged. This is important considering that the reliable identification of potential introduced pests and invasive species requires accurate methods, preventing errors in assessing and monitoring their geographic spread.

In an increasingly globalized world, biological invasions are one of the main causes of socioeconomic losses (Born et al. 2005, Jackson 2015, Diagne et al. 2021) as well as environmental damage (Kumschick et al. 2015, Blackburn et al. 2019). In this context, synanthropic silverfish can become significant pests in human environments, and therefore, accurate species identifications are crucial to establish their actual range and to promote control measures. Considering this, the main objectives of this work are as follows: (i) to establish reliable diagnoses for all the common synanthropic silverfish of the world based on appropriate morphological characters, (ii) to redescribe *Ctenolepisma calvum* and *Ctenolepisma (Sceletolepisma) villosum*, species whose descriptions are not up to date, (iii) to provide an updated key to identify all synanthropic species of silverfish, (iv) to establish reliable molecular diagnostic methods for these species and correct errors of identification in the literature and in DNA sequence repositories, and (v) to assess the distribution range and geographic origin of all of these species, considering only reliable records and the information about their habitat.

## Materials and Methods

### Morphological Study

Examined specimens were collected by the authors or were loaned from several scientific collections. Fresh material was obtained in occasional and accidental collections and not in exhaustive sampling, given that enough specimens of all common synanthropic species for a comparative study were available from these collections. Specimens were hand-collected and then fixed and preserved in 70% ethanol. For several specimens, an entomological aspirator was used. A list of all new records (specimens never published before and used for this work) is detailed in Supplementary Material 1, but the material supporting the redescriptions of *Ctenolepisma calvum* and *Ctenolepisma* (*Sceletolepisma*) *villosum* is indicated in the main text of the paper. Representative specimens of each studied species are deposited in the collection of the Department of Zoology of the University of Córdoba, Spain (UCO). For the morphological study, most specimens were examined with a binocular stereomicroscope Nikon SMZ-10 for preliminary identification, and some dissections were made to confirm the identity of some of the specimens. Dissected insects were mounted on slides using Tendeiro medium (Molero-Baltanás et al. 2000) and examined with a light microscope Nikon Labophot. Pencil drawings were prepared using photographs taken by a Nikon DS-Fi1 and a camera lucida attached to the same microscope. Several specimens belonging to most synanthropic species were examined under Scanning Electron Microscopy (SEM). Selected silverfish were first dehydrated with absolute alcohol and then with hexamethyldisilazane (Ubero-Pascal et al. 2005). Finally, they were coated with gold or gold-palladium prior to observation. Most specimens were examined and photographed using a JEOL JSM 6300 SEM in the microscopy service of the Servicio Central de Apoyo a la Investigación (SCAI) from the University of Córdoba (Spain). A specimen of *C. villosum* and another of *T. domestica* were examined and photographed using a Zeiss EVO LS15 SEM in the microscopy service of the Centro de Investigación, Tecnología e Innovación (CITIUS) from the University of Seville (Spain). SEM photographs not included in the main text of this work can be found in Supplementary Material 2.

Species identification was carried out using the most updated descriptions existing in the literature. The characters presented in these descriptions have been checked, and additional morphological characters that are used in the current Zygentoma taxonomy were evaluated to provide complete and updated diagnoses and descriptions. For comparisons, several reference specimens have been studied, mainly available in the University of Córdoba (UCO; Spain) collection, but also in the Museum of Natürkunde of Berlin (MNKB; Germany), the collection of Luis F. Mendes now deposited in the Museu Nacional de História Natural e da Ciência (MUHNAC; Portugal) and the Museo Nacional de Historia Natural (MNCN; Spain).

Not only synanthropic species have been studied, but also free-living species that can be confused with those from houses due to their morphological resemblance, as well as species that can be found occasionally in dwellings or other anthropic environments. An annotated list of all the similar taxa with the morphological differences and habitat preferences coming from literature and/or from studied material is given in Supplementary Material 1. In addition, a taxonomic interpretation guide of the most used diagnostic characters of Zygentoma, together with illustrations and explanations of the terminology, especially for the nonspecialist scientists and the public, is provided in Supplementary Material 2. Although most works cited the genus *Lepisma* and *Ctenolepisma* as feminine, recent nomenclatural changes have assigned them to neuter gender, resulting in corresponding changes to the endings of some species names (Molero-Baltanás et al. 2016, International Commission on Zoological Nomenclature, 2018). Therefore, the nomenclature used in this work will follow these recent changes.

### Molecular Study

*DNA barcoding study*. For the DNA barcoding study, we have considered 3 groups of sequences: (i) synanthropic species (*Ctenolepisma longicaudatum*, *Ctenolepisma lineatum*, *Ctenolepisma calvum*, *Ctenolepisma targionii/villosum*, *Ctenolepisma rothschildi*, *Acrotelsa collaris*, *Lepisma saccharinum*, and *Thermobia domestica*). There is one species for which we did not have DNA sequences accurately identified, but we have provided a sample that probably corresponds to this species (*Thermobia aegyptiaca*). These sequences were obtained from (a) Lepismatidae species that have been previously published and deposited in BOLD or GenBank, some of them probably incorrectly identified, and (b) sequences obtained for this work of new material of synanthropic species of Lepismatidae that have been identified by us, belonging to subfamilies Lepismatinae (genus *Lepisma*), Acrotelsatinae (genus *Acrotelsa*) and Ctenolepismatinae (genera *Ctenolepisma* and *Thermobia*). This is intended to include all or most of the common synanthropic species of Lepismatidae in the molecular studies; (ii) Sequences of some free-living species that are probably related to the synanthropic species and that can help to assess the correct identity of previously published sequences: *Ctenolepisma ciliatum* (Dufour, 1831), *Ctenolepisma nicoletii* (Lucas, 1846), *Thermobia nebulosa*  Irish, 1988, and several taxa of the genus *Neoasterolepisma*  Mendes, 1988. The origin and new data from specimens referred to in (2) and (3) are provided in Supplementary Material 1; (iii) an outgroup, *Tricholepidion gertschi*, belonging to the family Lepidotrichidae. Detailed information on the origin of all sequences used in this study is provided in Table 1. The complete dataset and all specimen data is available at BOLD as a public dataset: "DS-ZYANTH.

**Table 1.** Specimens and DNA sequences used for molecular study. Specimens reliably identified by us and belonging to genera including synanthropic species are marked in bold. Published identifications that this study suggests are incorrect are shown in quotations. The column “Institution producing DNA sequence” indicates the origin of those sequences mined from GenBank

Identification^a^	BOLD sample ID	BOLD process ID	Museum accession Nº.	GenBank accession Nº.	Institution producing DNA sequence
*Tricholepidion gertschi*	NC_005437	GBMTG421-16		NC_005437	University of Siena
***Acrotelsa collaris*** ^a^	gbs005977	ZYII363-21	K541563	OR732107	Australian Museum, Sydney
***Ctenolepisma calvum*** ^a^	gbs006285	ZYII423-23	K541644	OR732096	Australian Museum, Sydney
*Ctenolepisma calvum* ^a^	LC719153	GBMNE89871-22		LC719153	National Research Institute for Cultural Properties, Tokyo
*Ctenolepisma calvum* ^a^	LC719154	GBMNE89872-22		LC719154	National Research Institute for Cultural Properties, Tokyo
*Ctenolepisma calvum* ^a^	LC719155	GBMNE89873-22		LC719155	National Research Institute for Cultural Properties, Tokyo
*Ctenolepisma calvum* ^a^	LC719156	GBMNE89874-22		LC719156	National Research Institute for Cultural Properties, Tokyo
*Ctenolepisma calvum* ^a^	MG_Ctenolepisma_1	PLLEP007-21		OP028702	University of Lodz
*Ctenolepisma calvum* ^a^	MG_Ctenolepisma_2	PLLEP008-21		OP028703	University of Lodz
*Ctenolepisma calvum* ^a^	MG_Ctenolepisma_3	PLLEP009-21		OP028701	University of Lodz
***Ctenolepisma ciliatum***	gbs005751	ZYII349-21	K541561	OR732105	Australian Museum, Sydney
***Ctenolepisma lineatum*** ^a^	gbs006284	ZYII422-23	K541643	OR732093	Australian Museum, Sydney
***Ctenolepisma longicaudatum*** ^a^	gbs001275	ZYI064-18		MK185702	Australian Museum, Sydney
***Ctenolepisma longicaudatum*** ^a^	gbs005759	ZYII351-21	K541555	OR732097	Australian Museum, Sydney
“Ctenolepisma longicaudatum”	gbs001836	ZYI065-18	K377675	MT674899	Australian Museum, Sydney
“Ctenolepisma longicaudatum”	N/A	N/A		NC_073550	Institute of Scientific & Technical Research on Archives, Beijing
***Ctenolepisma nicoletii***	gbs005764	ZYII352-21	K541559	OR732102	Australian Museum, Sydney
***Ctenolepisma nicoletii***	gbs005766	ZYII420-23		OR732091	Australian Museum, Sydney
***Ctenolepisma rothschildi*** ^a^	gbs003964	ZYII320-21	K261295	OR732095	Australian Museum, Sydney
***Ctenolepisma targionii*** ^a^	gbs005769	ZYII353-21	K541573	OR732092	Australian Museum, Sydney
*Ctenolepisma villosa* ^a^	N/A	N/A		NC_046478	Wannan Medical College, Wuhu
*Lepisma saccharinum* ^a^	Ajab_Lepisma_1	PLLEP001-21		OP028710	University of Lodz
*Lepisma saccharinum* ^a^	AJaz_Lepisma_1	PLLEP010-21		OP028707	University of Lodz
*Lepisma saccharinum* ^a^	Ajaz_Lepisma_2	PLLEP011-21		OP028709	University of Lodz
*Lepisma saccharinum* ^a^	BIOUG02887-D01	JSTW008-12		KR141905	Center for Biodiversity Genomics
*Lepisma saccharinum* ^a^	BIOUG27448-E09	JSTW016-16		MG382617	Centre for Biodiversity Genomics
***Lepisma saccharinum*** ^a^	gbs006126	ZYII366-21	K541610	OR732104	Australian Museum, Sydney
***Lepisma saccharinum*** ^a^	gbs006127	ZYII367-21	K541611	OR732108	Australian Museum, Sydney
*Lepisma saccharinum* ^a^	KBS_Lepisma_1	PLLEP003-21		OP028704	University of Lodz
*Lepisma saccharinum* ^a^	KBS_Lepisma_2	PLLEP005-21		OP028706	University of Lodz
*Lepisma saccharinum* ^a^	LC492866	GBMNA33209-19		LC492866	National Research Institute for Cultural Properties, Tokyo
*Lepisma saccharinum* ^a^	MG_mom_Lepisma_1	PLLEP006-21		OP028708	University of Lodz
*Lepisma saccharinum* ^a^	TR_Lepisma_1	PLLEP002-21		OP028711	University of Lodz
*Lepisma saccharinum* ^a^	TR_Lepisma_2	PLLEP004-21		OP028705	University of Lodz
‘Lepisma saccharinum’	N/A	N/A		NC_047445	Guiyang University
*Neoasterolepisma* sp. *DY-191*	MH279719	GBMH18672-19		MH279719	University of Kentucky
*Neoasterolepisma* sp. *DY-192*	MH279720	GBMH18673-19		MH279720	University of Kentucky
*Neoasterolepisma foreli*	N/A	N/A		MT982147.1	University of Siena
***Thermobia* sp.** ^a^	gbs005947	ZYII240-20	K541538	OR732099	Australian Museum, Sydney
“*Thermobia domestica*”	19_BHS_PC_11	SDP819010-19		MN448219	University of New England
“*Thermobia domestica*”	19_UNE_DL_04	SDP864013-19		MN556951	University of New England
“*Thermobia domestica*”	AF370848	GBMNA9236-19		AF370848	Harvard University
“*Thermobia domestica*”	BIOMTWL-INS130	LGEN065-14		OR732100	Gujarat State Biotechnology Mission
“*Thermobia domestica*”	BIOUG14963-D06	GMBUA1578-14		OR732106	Center for Biodiversity Genomics
“*Thermobia domestica*”	BIOUG30669-H11	JSTW017-16		MG375320	Centre for Biodiversity Genomics
“*Thermobia domestica*”	BOLD-1VBF2OZU7	MOBIL1017-15		OR732101	Center for Biodiversity Genomics
***Thermobia domestica*** ^a^	gbs006286	ZYII424-23	K541645	OR732103	Australian Museum, Sydney
*Thermobia domestica* ^a^	JN970940	GBMIN29272-13	MNHN:EA010046	JN970940	Museum National d’Histoire Naturelle
*Thermobia domestica* ^a^	NC_006080	GBMTG482-16		NC_006080	University of Cambridge
*Thermobia domestica* ^a^	N/A	N/A		DQ280136.1	Harvard University
***Thermobia nebulosa***	gbs005973	ZYII361-21	K541577	OR732094	Australian Museum, Sydney
***Thermobia nebulosa***	gbs005974	ZYII421-23		OR732098	Australian Museum, Sydney

^a^Synanthropic species are marked with a superscript letter, but this letter is not used for incorrectly identified singletons.

*DNA extraction, PCR, and DNA sequencing*. One leg was removed from each specimen and stored in 100% ethanol. DNA was extracted from the leg using the Bioline Isolate II Genomic DNA Kit (Bioline, Eveleigh, NSW, Australia) following the manufacturers’ protocols, except that DNA was eluted in 70 µl of extraction buffer (https://dx.doi.org/10.5883/DS-ZYANTH). Polymerase chain reaction (PCR) amplification of the DNA barcode region of the mitochondrial COI gene used the primers and followed the method of Mitchell (2015). PCR products were purified using ExoSAP and sequenced in both directions using ABI Big Dye Terminator v.3.1 chemistry by Macrogen Inc. (Seoul, South Korea).

*DNA sequence assembly and phylogenetic analysis*. Forward and reverse direction sequence trace files were assembled using Geneious Prime v.2022.2.1 (Kearse et al. 2012). DNA sequences, sequence trace files, and specimen collection data were submitted to BOLD and GenBank. The 15 new sequences were aligned with the 37 public domain sequences using Muscle (Edgar 2004) and adjusted by eye. MEGA v.11 (Tamura et al. 2021) was used to select the most appropriate model of DNA evolution, which proved to be the General Time Reversible model with Gamma-distributed rates and invariant sites (GTR + G + I).

Phylogenetic analyses were performed using maximum likelihood (ML) as implemented in RAxML 7.2.8 (Stamatakis 2006) and Bayesian inference in MrBayes v.3.2.6 (Ronquist et al. 2012) using the plugins available in Geneious. RAxML analyses used the ML search convergence criterion and performed 1,000 fast bootstrap replicates. MrBayes analyses used 4 heated chains with chain temperature = 0.2, chain length initially set to 25,000,000 generations, subsampling every 1,000 generations, but with the stopping rule used to end the analysis when the average standard deviation of splits frequencies reached 0.01, with the burn-in fraction set to 0.25.

### Distributional Study

Regarding the “synanthropic” definition provided by Gullan and Cranston (2014) about species associated with humans or their dwellings, 10 silverfish species are classified as synanthropic (but see Results section about *C. targionii* and *C. villosum* reducing the number to 9). These species (mentioned in the introduction of this work) can be located indoors (domestic habitat) or outside, around buildings and other structures (peridomestic habitat), following the criterion of Robinson (2005). Additional species occasionally found in these habitats but whose records represent less than 10% of their total findings are not considered in this work. However, several of these species are mentioned at the end of the *Results* section.

An exhaustive bibliographic search was also carried out to locate all the available information on the occurrence and habitats of these synanthropic silverfish species. In assessing the geographic distribution of each species, we have considered only those records where Zygentoma species have been identified by Zygentoma specialists or by nonspecialists, but they provide enough morphological evidence to corroborate their identification. Only those records that presented concrete information about their locality were considered. To assess the biogeographic origin of the studied silverfish, we have only considered works where the studied specimens are referenced with the habitat where they were found. To contribute to this objective, the unpublished habitat of several previous Spanish records has been included. For this search, we used as keywords the genus or the scientific names (and their synonyms; a list of all synonyms for each species can be found in Supplementary Material 1) in bibliographic databases such as Google Scholar or Web of Science. A complete and detailed list of considered records based on reliable identifications is given in Supplementary Material 3. We also examined several informative works (as magazines, encyclopedias, or websites) and available records based on photographs at citizen science platforms. Considering that most of these photographs do not show enough diagnostic characters to ensure their specific identity, the information from all these databases was not deeply analyzed or considered. Only in some cases (for example, in species with a low number of reliable records, such as *C. calvum*) are reasonable records included in maps with a question mark. Several examples of misidentification errors or confusion coming from scientific literature and some informative works are commented on in Supplementary Material 4. Maps were generated with ArcGis Desktop 10.8.1.

## Results

### Molecular Study

**Fig. 1.**
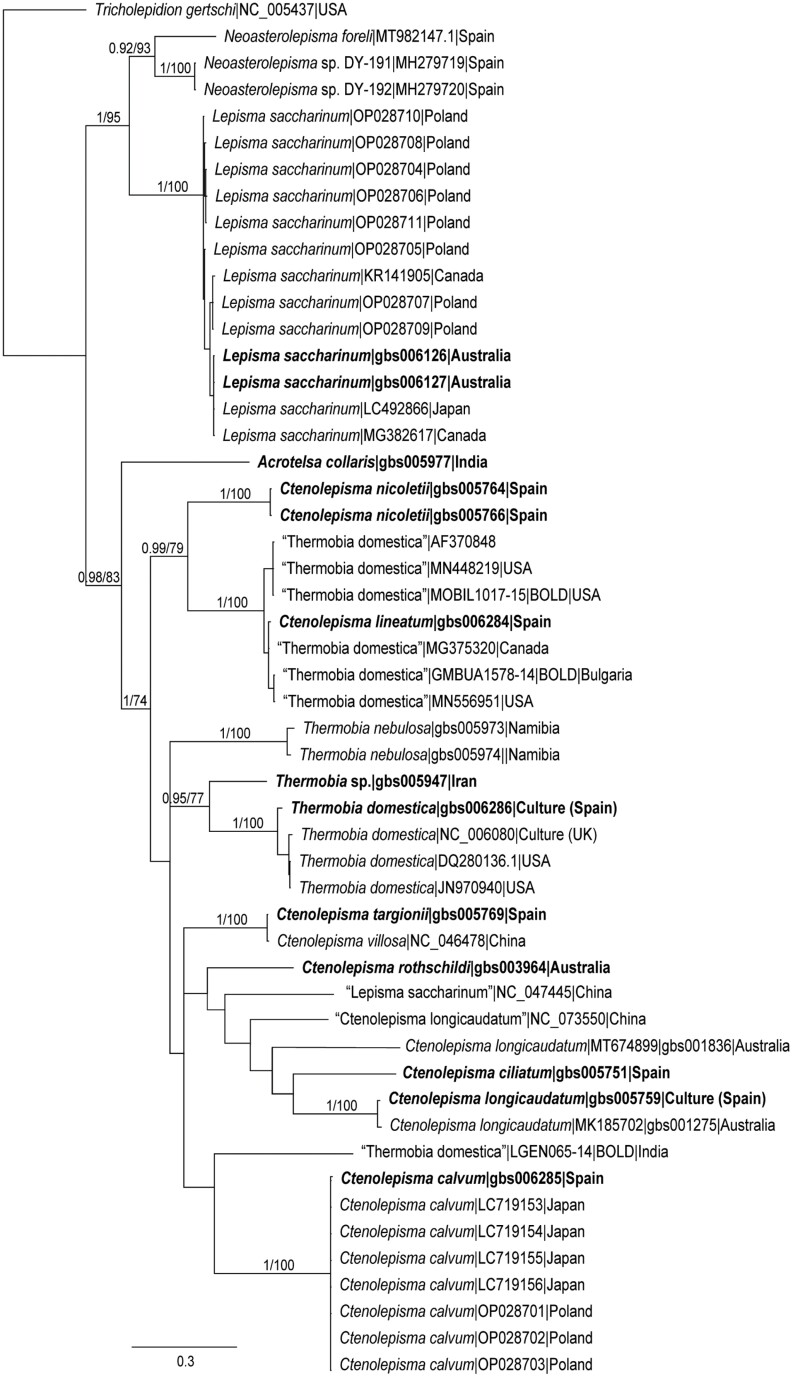
Bayesian tree for known COI sequences of silverfish belonging to synanthropic species together with related species and *Tricholepidion gertschi* as outgroup only. Taxon names comprise species names as recorded in online databases, GenBank accession number (or, in 2 instances BOLD Process ID if the sequences are only in BOLD), and the specimen’s country of origin if known. The numbers above branches are Bayesian posterior probability and RAxML bootstrap percentage, shown only if both numbers are greater than or equal to 0.9 and 70, respectively. Reliable identifications of specimens studied by us and belonging to genera, including synanthropic species, are highlighted in bold italics font, and names corresponding to incorrectly identified specimens are written between quotation marks. The name *Ctenolepisma targionii* is maintained to justify the synonymy of this species with *C. villosum* (named as *C. villosa* by the authors of the COI sequence).

Bayesian and ML analyses yielded almost identical trees, differing only in their degree of resolution. Figure 1 shows the lesser resolved Bayesian tree, with posterior probabilities and ML bootstrap values shown above nodes only if values were ≥0.90% and ≥70%, respectively (strong support). The focus of these analyses was on species identification, not on higher-level relationships; nevertheless, Lepismatinae and Ctenolepismatinae both recovered as monophyletic. Each of the synanthropic species recognized in this study is represented in this tree by at least one reliably identified specimen (in bold font in Fig. 1). The specimen indicated as *Thermobia* sp. was not in a very good condition and its specific identity was not sure but considering that it does not match with *T. domestica* and that *T. aegyptiaca* occurs in Iran (where the species was collected), it is very likely that it corresponds to this latter species. There are striking discrepancies between some of our sequences from reliably identified specimens and the names assigned to matching GenBank and BOLD sequences. Where we have inferred that the latter sequences are misidentified, we put their published incorrect identities in quotes.

For *Lepisma saccharinum* almost all sequences cluster together with our new sequences (OR732104 and OR732108). However, the notable exception is a sequence from China that falls within the *Ctenolepisma* clade. Significantly, this sequence was extracted from a complete mitochondrial genome sequence (Bai et al. 2020), reference genome NC_047445. Despite this complete mitochondrial sequence being afforded reference sequence status on GenBank, the species name assigned to it is clearly incorrect. The name *Thermobia domestica* is applied to 3 unrelated clusters of sequences, one of which contains our reliably identified specimen (OR732103). The correctly identified cluster contains samples from the United Kingdom (including a complete mitochondrial genome, accession number NC_006080) and 2 samples from the United States. Of the other 2 clusters, one clearly is *Ctenolepisma lineatum*, as it contains our reliably identified sample of that species (OR732093). Six incorrectly identified samples in this cluster were from the United States, Canada, and Bulgaria. The third cluster is a singleton sample from India, also placed in the *Ctenolepisma* clade. *Ctenolepisma longicaudatum* is represented by 4 samples, which are placed in 3 clusters. Our reliably identified sample (OR732097) from a lab culture in Spain is almost identical to a sample from Australia (MK185702), but this cluster is separated from the other 2 singleton clusters by a separate cluster containing our reliably identified sample of *C. ciliatum* (OR732105). Further research is required to establish whether this molecular variation is representative of cryptic species diversity or incorrect identifications (see the section on morphological remarks on *C. longicaudatum*).

Furthermore, our sequence of *Ctenolepisma targionii* and the available one of *Ctenolepisma villosum* are clustered together, congruent with them belonging to the same species. This supports the synonymy presented below in the morphological section of *Results*. For *Ctenolepisma calvum*, our reliably identified sample (OR732096) clusters with published sequences of the same species. Finally, we have provided the first available COI sequences for the synanthropic species *Acrotelsa collaris* and *Ctenolepisma rothschildi* and the free-living species *Ctenolepisma ciliatum*, *Ctenolepisma nicoletii*, and *Thermobia nebulosa*.

### Morphological Study and Geographic Range

We have provided for each species of synanthropic silverfish: (i) a brief diagnosis, (ii) a commentary on the most relevant morphological characters for separating the species from related synanthropic or free-living species (although details are presented in Supplementary Material 1), and (iii) an approximation of its geographic distribution and its region of origin. For *Ctenolepisma calvum* and *Ctenolepisma villosum* the diagnosis is replaced by an entire redescription and a specific discussion of taxonomic characters. For a detailed overview of the material examined and a list of synonyms for each species, see Supplementary Material 1. Here, we also provide a new identification key for the most common synanthropic silverfish (most characters used in the key are thoroughly explained in Supplementary Material 2).

### Identification Key

Figs. 2–16

**Fig. 2.**
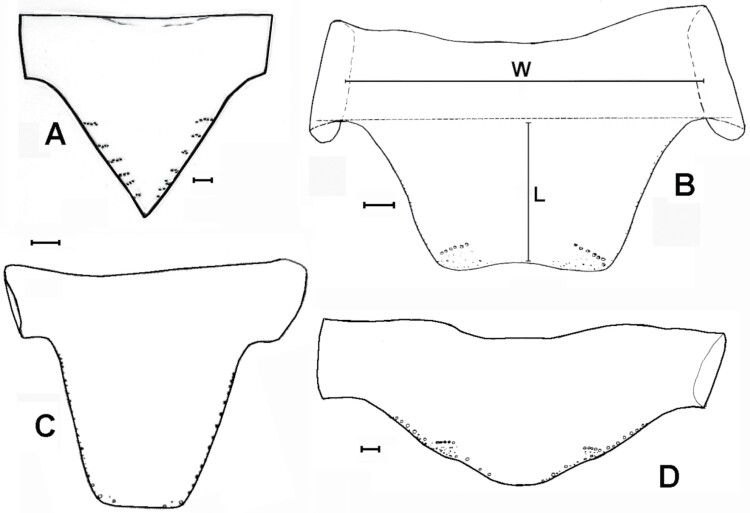
Shape and chaetotaxy of urotergites X of some representative species of synanthropic Lepismatidae. A) *Acrotelsa collaris*. B) *Ctenolepisma longicaudatum* (W: width, L: length of the trapezoidal posterior expansion of the tergite). C) *Lepisma saccharinum*. D) *Ctenolepisma lineatum*. Drawing B) is based on Mendes (1982). Scales: 0.1 mm.

**Fig. 3.**
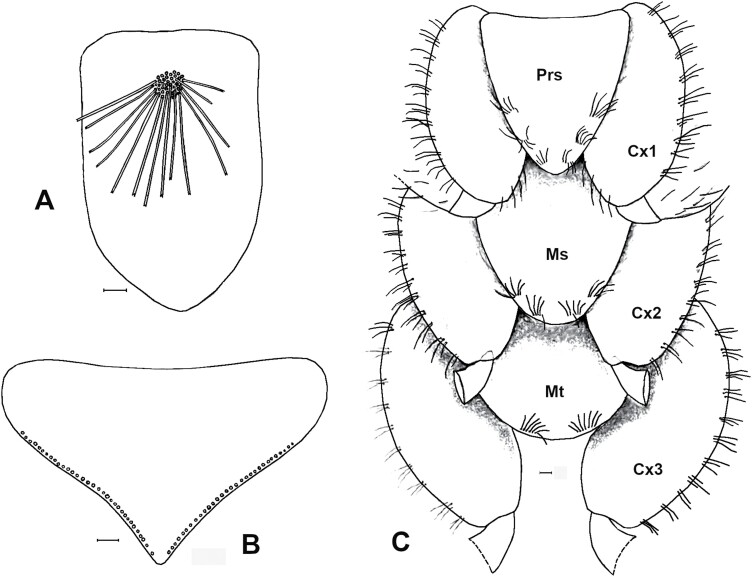
A) Prosternum of *Acrotelsa collaris*, with a median tuft of macrochaetae. B) Metasternum of the same species, only with marginal setae. C) Ventral view of the thorax of *Ctenolepisma* sp., showing sternites covering the base of coxae; adapted from a drawing of Escherich (1905). Prs: prosternum. Ms: mesosternum. Mt: metasternum. Cx1, Cx2, and Cx3: coxae of the 1st, 2nd, and 3rd legs. Scales: 0.1 mm.

**Fig. 4.**
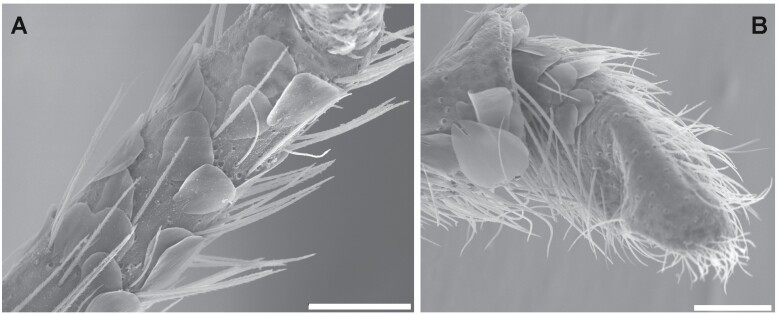
A) *Acrotelsa collaris* (Cape Verde). Penultimate article of the maxillary palp with orbicular scales (rounded to subrectangular) and some setae. B) Penultimate and last articles of the labial palp of the same species with orbicular scales that are only present in the last article. Photographs taken by N. López. Scales: 0.1 mm.

**Fig. 5.**
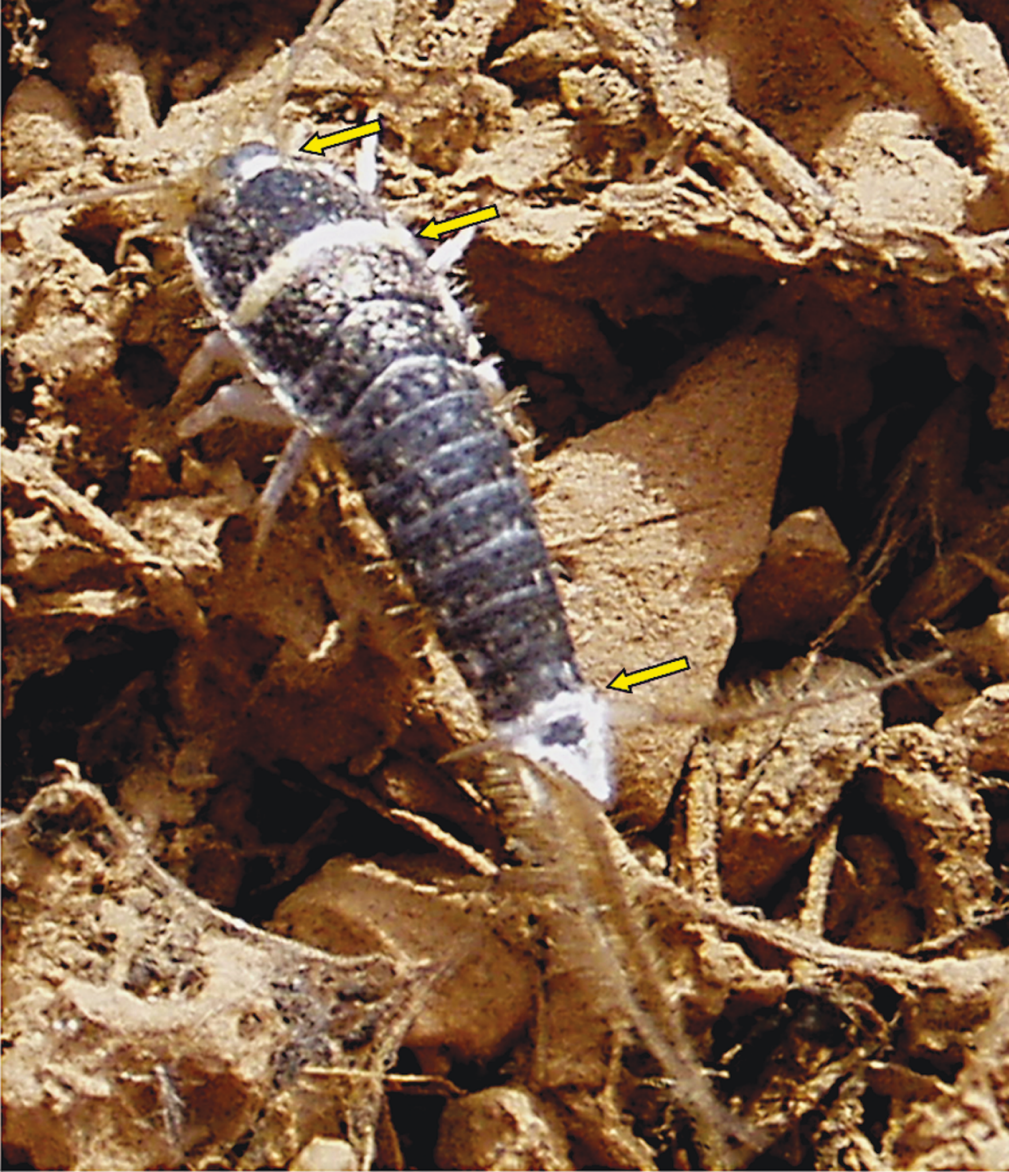
*Acrotelsa collaris* (Cape Verde. Author: Néstor López). Living specimen. Transverse bands of white scales are marked with arrows. The body length of the specimen is about 11 mm.

**Fig. 6.**
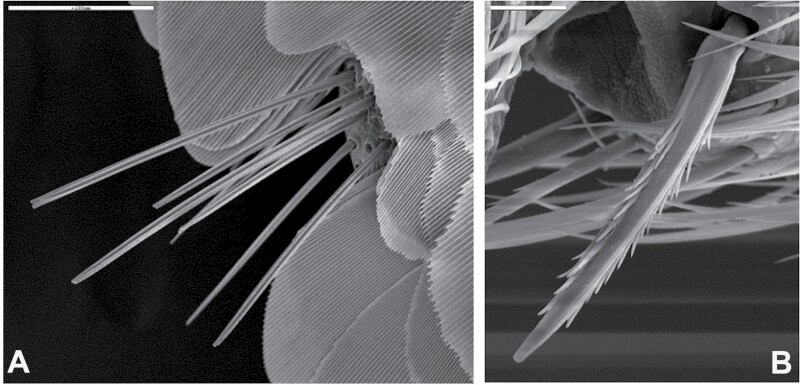
A) A bristle-comb of smooth apically bifid macrochaetae (*Allacrotelsa kraepelini*, Spain). B) A feathered macrochaetae (*Ctenolepisma nicoletii*, Spain). Scales: 0.1 mm A) and 25 µm B).

**Fig. 7.**
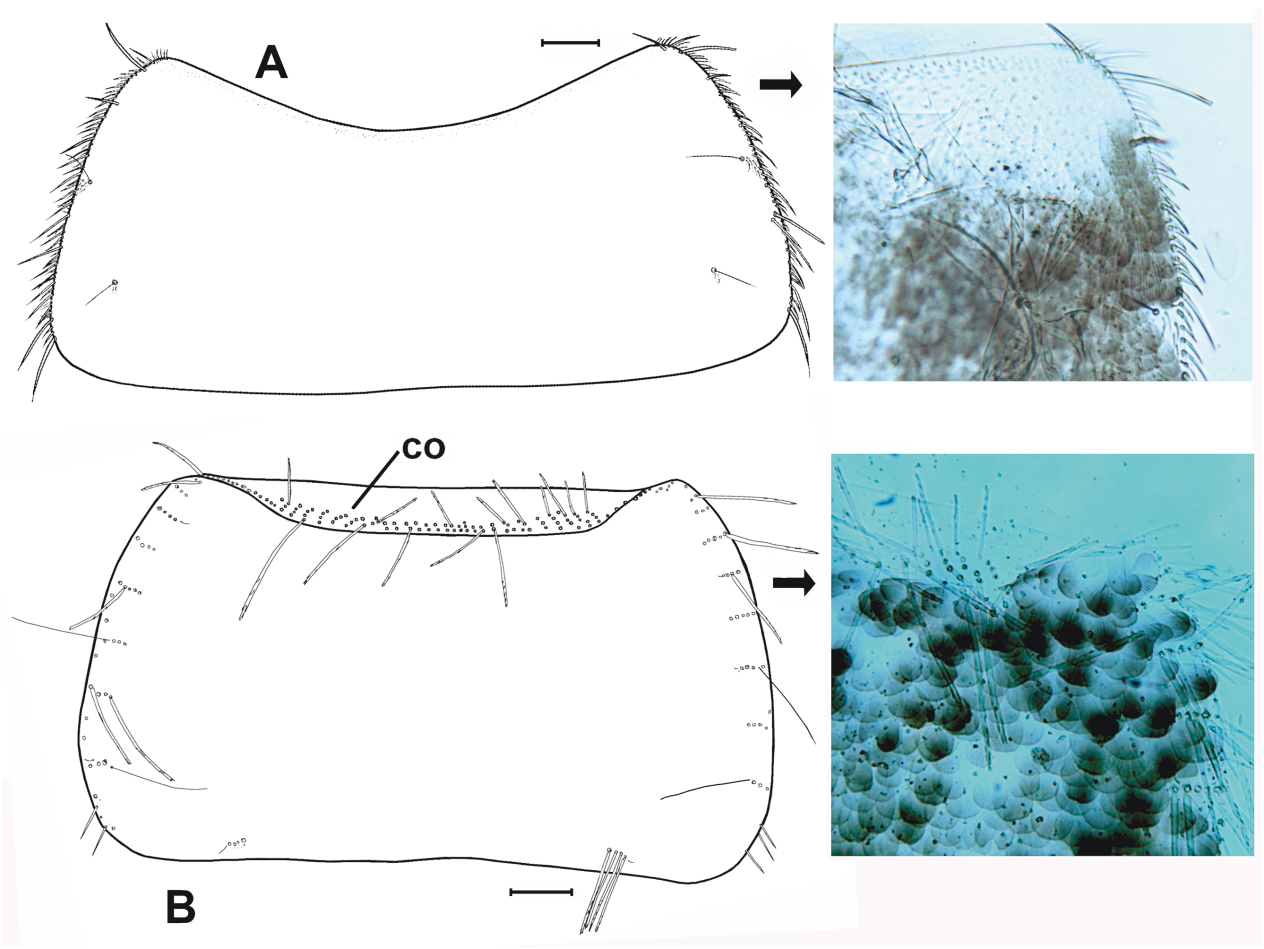
A) Pronotum of *Lepisma saccharinum*, showing the absence of setal collar, with a detail of the anterolateral corner in a micrograph. B) Pronotum of *Ctenolepisma* sp. (probably *C. nicoletii*), showing a setal collar in its anterior margin. Scales: 0.2 mm.

**Fig. 8.**
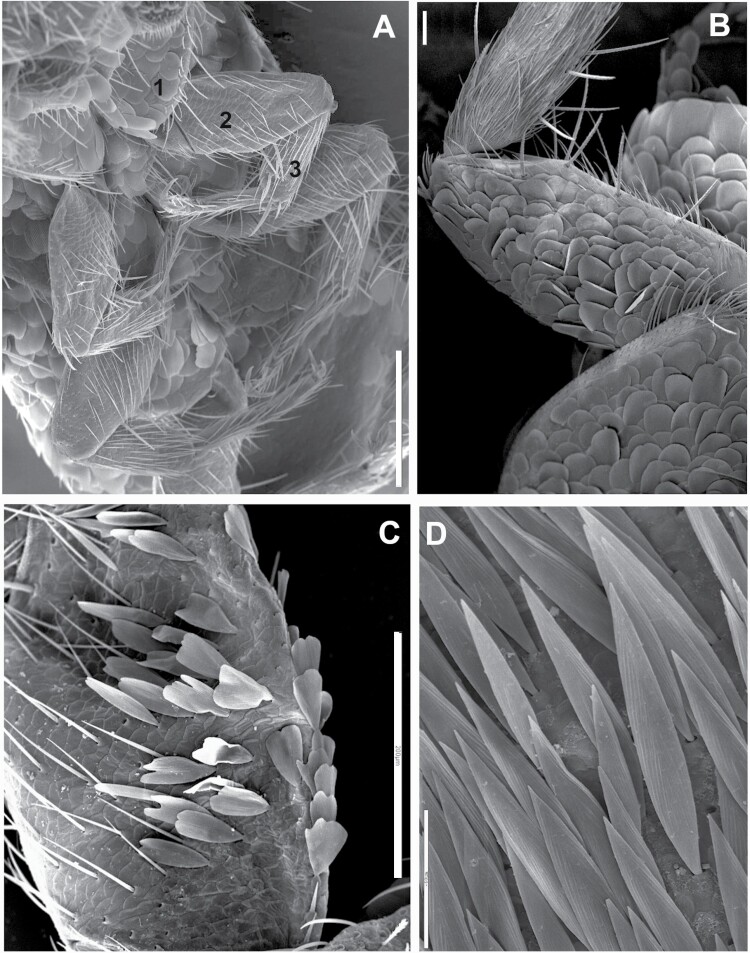
A) Ventral view of the thorax of *Lepisma saccharinum* (Spain), where coxae (1) can be seen covered by rounded scales and femora (2) and tibiae (3) of the first and second pair of legs are devoid of scales and covered only with setae. Scale: 0.25 mm. B) Ventral side of the second leg of *Ctenolepisma villosum* (Spain), showing coxa and femur covered with rounded scales and tibia devoid of scales, only covered with setae. Scale: 0.1 mm. C) Ventral side of the femur of *Ctenolepisma rothschildi* from Cape Verde, with scales with modified shape (subtriangular, truncated distally, and variable). Scale: 0.2 mm. D) Detail of the lanceolate scales of the ventral side of the femur of *Ctenolepisma lineatum* (Spain). Scale: 50 μm.

**Fig. 9.**
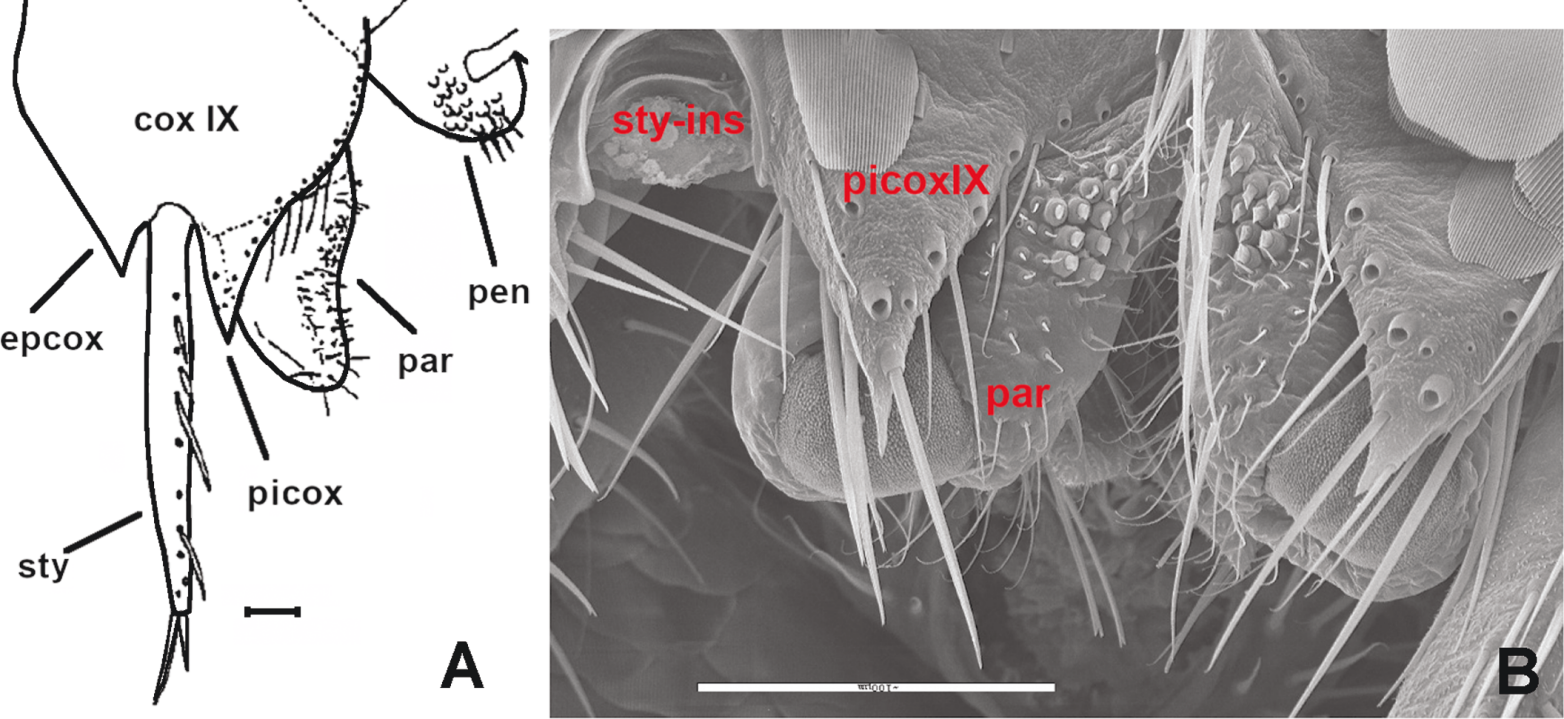
Paramera of *Lepisma saccharinum*. A) Right side of the ninth segment of a male, in ventral view, showing a hyperdeveloped paramere (par) that surpasses the apex of the inner process of the coxite IX (picox). coxIX: coxite IX (half part of the ventral plate of the ninth abdominal sternite); epcox: the outer process of the coxite IX; sty: stylus IX; pen: penis. B) SEM photograph of the paramera (par) associated with the inner process of the coxites IX (picoxIX). The insertion of a ninth stylus that has come off is visible (sty-ins).

**Fig. 10.**
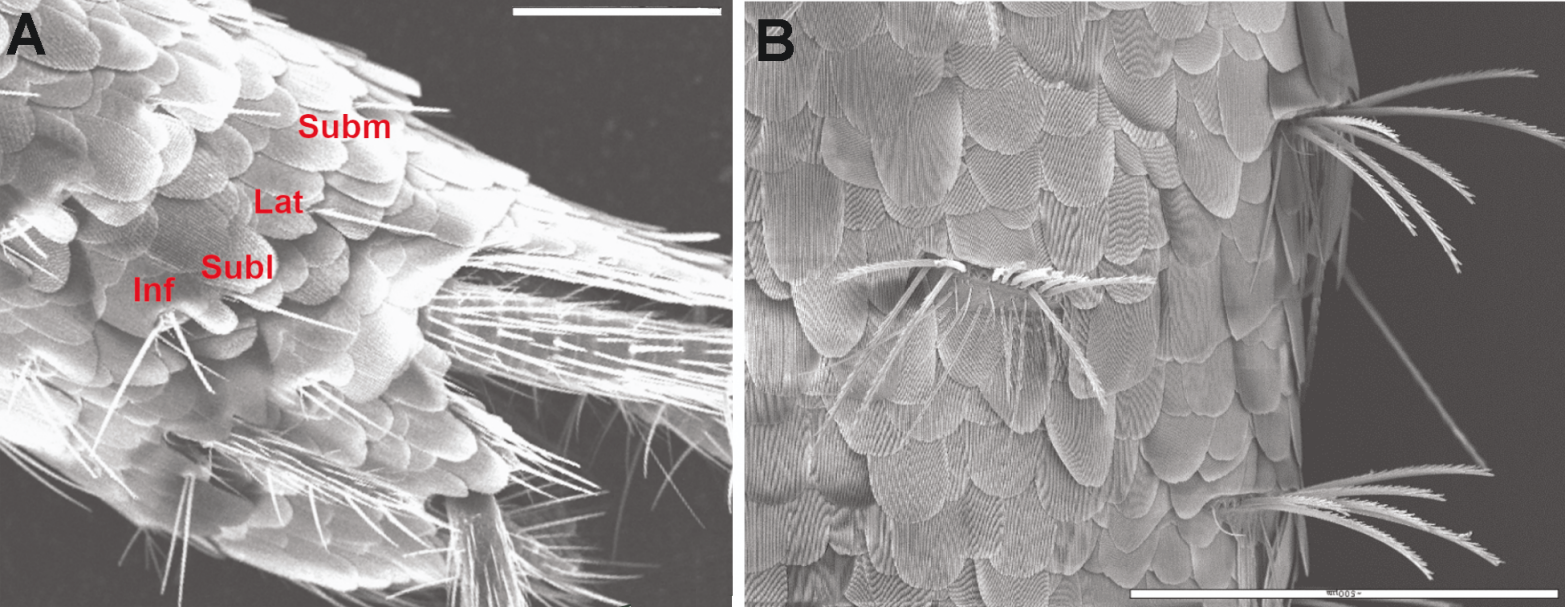
Chaetotaxy of abdominal tergites. A) Lateral view of the abdomen of *Lepisma saccharinum*, SEM photograph showing in the posterior margin of urotergite VIII the infralateral group of macrochaetae (Inf, with 2 macrochaetae and 1 outer thin seta), and isolated sublateral (Subl), lateral (Lat) and submedian (Subm) macrochaetae. B) Urotergal combs of *Ctenolepisma ciliatum* on lateral (right) and submedian position (left). As this SEM photo is taken in the dorsal view, infralateral combs (inserted in the most lateral part of the tergite that is bent toward the ventral side) are not visible. Scales: 0.5 mm.

**Fig. 11.**
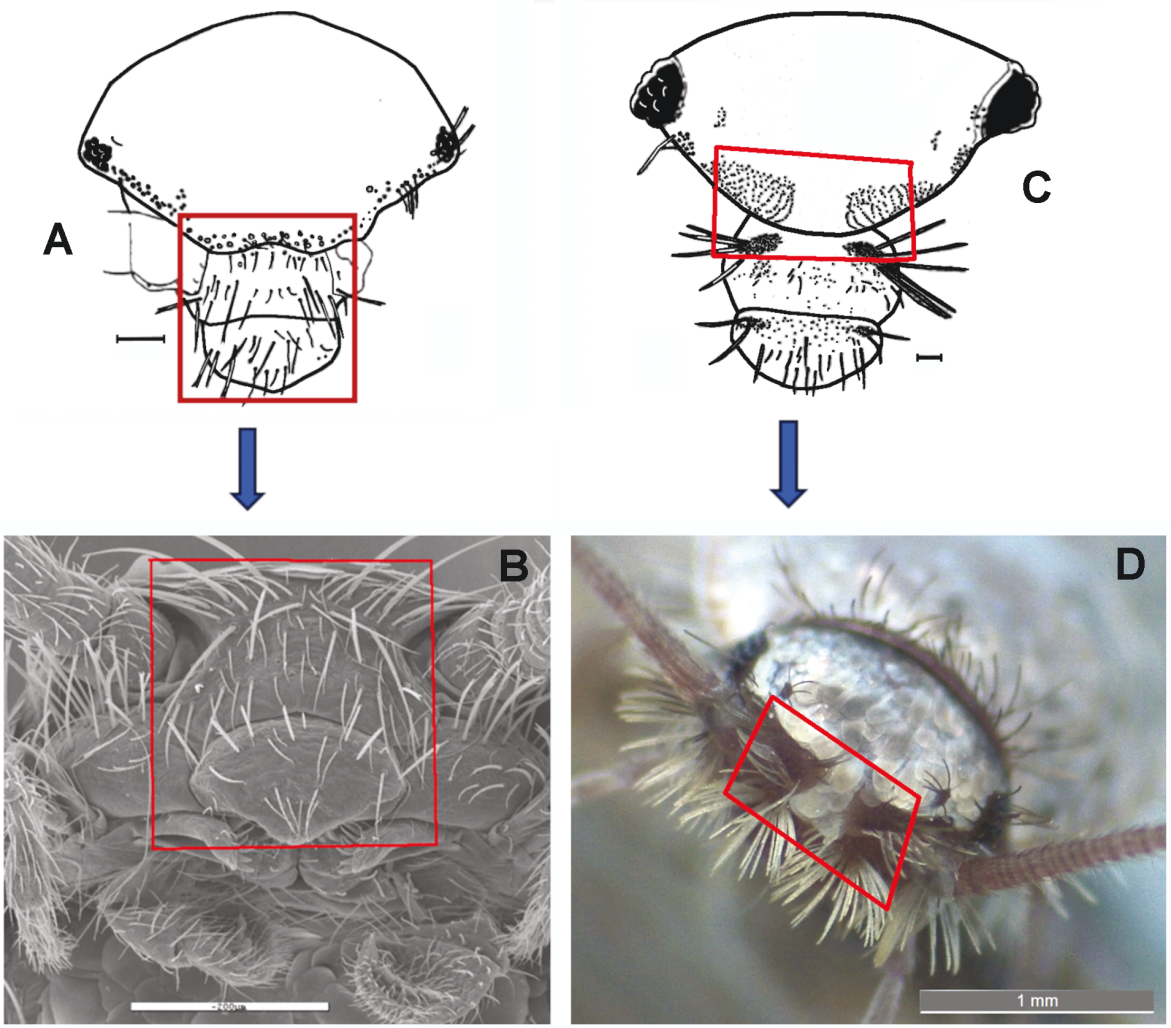
Frontal chaetotaxy of *Lepisma* and *Ctenolepisma*. A) Cephalic chaetotaxy of *Lepisma* sp*.* The region surrounded by the square (frontal margin, clypeus, and labrum) is visible in *Lepisma saccharinum* in B) (SEM photograph). There is no median gap in the row of frontal setae. C) Cephalic chaetotaxy of *Ctenolepisma ciliatum* (that of other *Ctenolepisma* species is similar). Frontal macrochaetae are represented only by their insertions to see their arrangement. The region surrounded by the red square (frontal and clypeal tufts with a median gap) is visible in *Ctenolepisma* (*Sceletolepisma*) *guadianicum* Mendes, 1992, with similar cephalic chaetotaxy, in D) (macrophotograph). Scales: 0.2 mm, except for D): 1 mm.

**Fig. 12.**
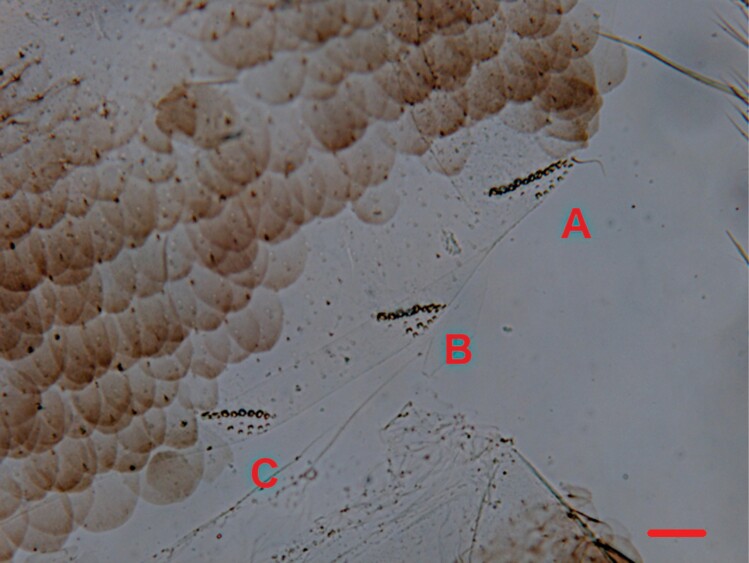
Micrograph of the hind margin of the right side of the urotergite V of *Ctenolepisma iranicum*, where the insertions of A) infralateral, B) lateral, and C) submedian combs of macrochaetae are visible. Scale: 0.1 mm.

**Fig. 13.**
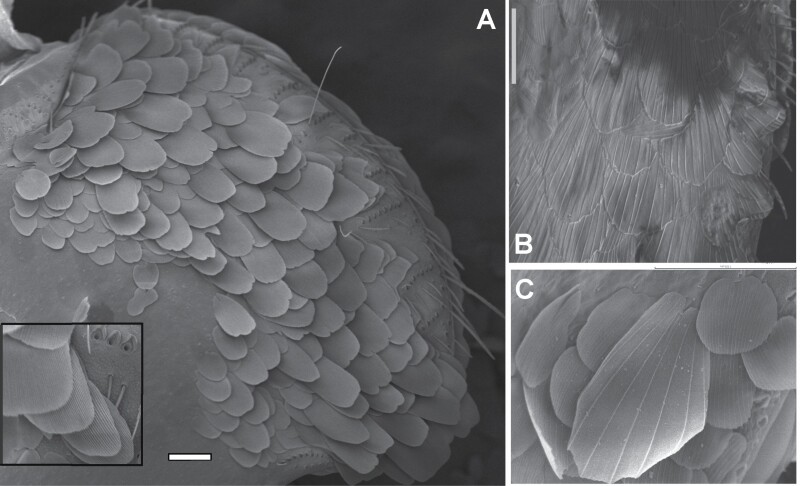
Dorsal scales, SEM photographs. A) The right half of the pronotum of *Thermobia domestica* shows uniform scales with numerous parallel ribs that are only distinguishable with high magnification (detailed photograph in the box at the bottom left of the picture). B) Uniform pauci-radiate scales (with a low number of speciated ribs) of the thoracic nota of *Ctenolepisma calvum*. C) Heterogeneous dorsal scales on the pronotum of *Ctenolepisma rothschildi*, showing a big pauci-radiate scale and a smaller multiratiate one. Scales: 0.1 mm.

**Fig. 14.**
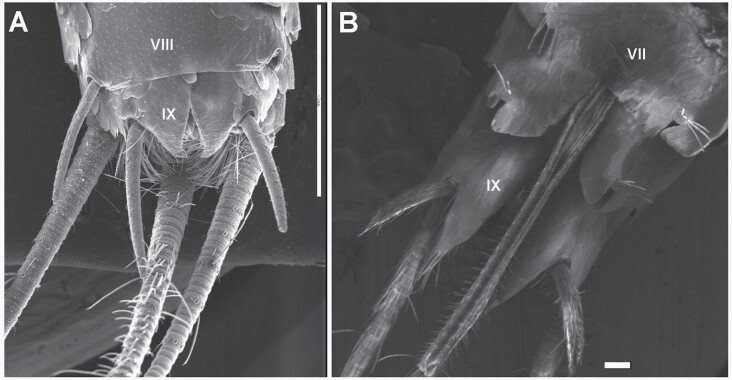
A) *Ctenolepisma nicoletii*, male. The ventral side of the last abdominal segments (VIII and IX) in the ventral view shows 2 pairs of styli (the left stylus on the eighth segment is come off, and only the insertion is visible on the right part of the photograph). The ninth sternite is divided into 2 lateral plates (coxites). B) *Ctenolepisma calvum*, female. The ventral side of the last abdominal segments in the ventral view (VII, VIII, and IX), showing 1 pair of styli in the ninth segment. The eighth and the ninth sternites are divided into lateral coxites, surrounding the ovipositor in the middle. The eighth coxites have bristle combs but lack styli. Scales: A) 1 mm and B) 0.1 mm.

**Fig. 15.**
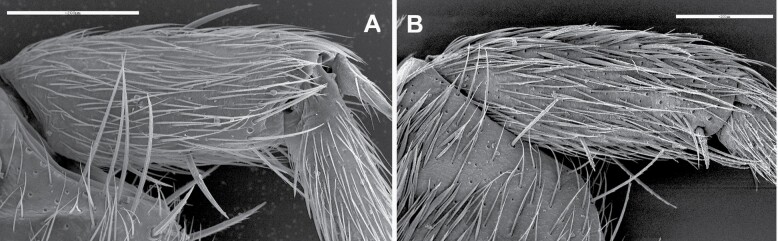
A) Protibia of *Ctenolepisma ciliatum*, covered with setae, lacking scales. B) Protibia of *Ctenolepisma lineatum*, with narrow lanceolate scales interspersed among setae. Scales: 0.2 mm.

**Fig. 16.**
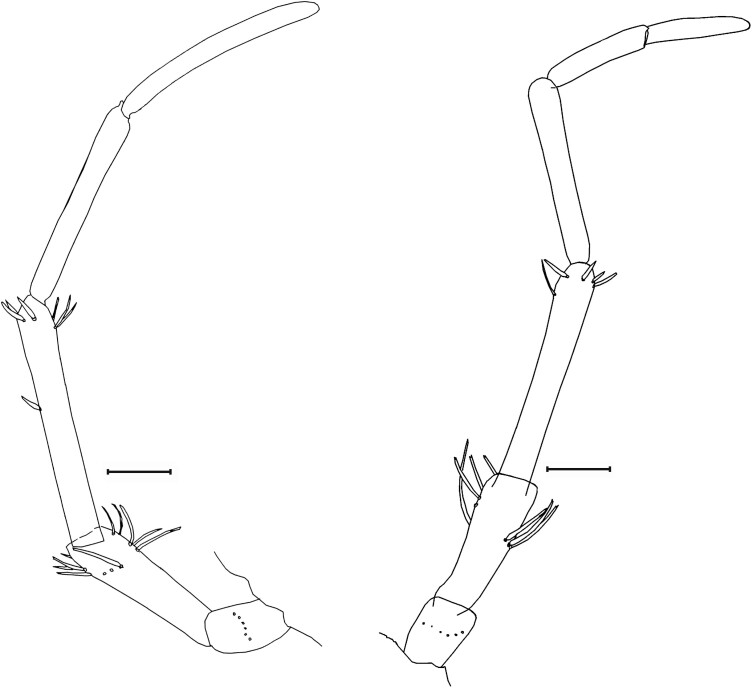
Maxillary palps of A) *Thermobia aegyptiaca* and B) *T. domestica*. Scales: 0.2 mm.

1. Urotergite X long, triangular, posteriorly acute, with more than 1 + 1 lateral combs of macrochaetae (Fig. 2A). Thoracic sternites covered by the basal part of coxae; prosternum very reduced and with a medial tuft of setae (Fig. 3A) and meso- and metasternum only with marginal setae (Fig. 3B). Maxillary and labial palps with scales (Fig. 4). Usually with some transverse bands of white scales dorsally (Fig. 5). ***Acrotelsa collaris***.
— Urotergite X trapezoidal, frequently short, with 1 + 1 lateral combs of macrochaetae close to its apex (Fig. 2B) or alternatively, longer but combs not clearly defined (Fig. 2C). If subtriangular, it is short and rounded posteriorly (Fig. 2D). Thoracic sternites developed, covering the basal part of coxae and with setae inserted on the margins; usually with macrochaetae arranged in combs close to the lateral margin or subapically (Fig. 3C). Maxillary and labial palps without scales. The dorsal pattern of scales is different. **2**.
2. Macrochaetae smooth (Fig. 6A). Pronotum without setal collar (Fig. 7A). Femora and tibiae without scales (Fig. 8A). Paramera large (Fig. 9). Urotergite X long trapezoidal (the trapezoid area clearly longer than 0.75 times its basal width in adults, Fig. 2C). Dorsal macrochaetae isolated, with the exception of a small infralateral group (Fig. 10A). Cephalic macrochaetae not arranged in tufts and without a median gap lacking setae on the middle of the frons (Fig. 11A). ***Lepisma saccharinum***.
— Macrochaetae feathered (Fig. 6B, 10B). Pronotum with setal collar (Fig. 7B). At least the inner side of femora with scales, sometimes smaller and with modified shape relative to those of coxae of the body (Fig. 8B). Paramera absent. Urotergite X is shorter, trapezoidal or not (when there is a trapezoid area, it is shorter than 0.6 times its basal width). Dorsal macrochaetae are arranged in combs (Figs. 10B and 12); in most urotergites 2 + 2 or 3 + 3 combs (i.e., 2 or 3 combs on each side of the hind margin of the abdominal tergite). Cephalic macrochaetae are arranged in tufts that leave a median gap in the middle of the frons (Fig. 11B). **3.**
3. Urotergite X trapezoidal, with straight or slightly convex or concave hind margin (Fig. 2B). 3 + 3 combs of macrochaetae on urotergites II–V or on urotergites II–VI. Urotergites VII and VIII with 2 + 2 combs of macrochaetae. **4.**
— Urotergite X is short, subtriangular, with a convex hind margin (Fig. 2D). Urotergites II–VII with 3 + 3 combs of macrochaetae and urotergite VIII with 2 + 2 combs of macrochaetae; alternatively, none of the urotergites has 3 + 3 combs of macrochaetae **7.**
4. Urotergite VI with 3 + 3 combs of macrochaetae. Urosternal combs usually have 15–25 macrochaetae each. ***Ctenolepisma longicaudatum***.
— Urotergite VI with 2 + 2 combs of macrochaetae. Urosternal combs with a lower number of macrochaetae, usually less than 10 (at most, 12 in some cases). **5.**
5. With median combs on urosternites. Dorsal scales are relatively homogeneous, with many closely spaced ribs (Fig. 13A). Femora with rounded scales (Fig. 8B). Males and females with different numbers of abdominal styli (males with 1 pair, females with 2 pairs). ***Ctenolepisma* (*Sceletolepisma) villosum.***
— Without median combs on urosternites. With abundant dorsal scales with few widely spaced ribs (Fig. 13B and C). Femora with modified scales (Fig. 8C). Both sexes have the same number of abdominal styli (1 or 2 pairs). **6.**
6. Both sexes have 2 pairs of abdominal styli when adults (Fig. 14A). Dorsal scales are heterogeneous, some bigger with widely spaced ribs and others smaller with more closely spaced ribs (Fig. 13C). ***Ctenolepisma rothschildi***.
— Both sexes with 1 pair of abdominal styli (Fig. 14B). Dorsal scales more homogeneous in size and density of ribs (Fig. 13B). ***Ctenolepisma calvum***.
7. Scales of femora broad and rounded, similar to those of coxae and body (as in Fig. 8B). Tibiae without scales (as in Fig. 15A). Urotergites II–VII with 2 + 2 combs of macrochaetae. Urosternites III–VI with a median comb. genus ***Thermobia*** … **8**.
— Scales of femora are narrow and lanceolate (Fig. 8D), very different from those of coxae and body. Tibiae has small lanceolate scales, is narrow, and has an acute apex (Fig. 15B). Urotergites II–VII have 3 + 3 combs of macrochaetae. Urosternites without median combs. ***Ctenolepisma lineatum***.
8. Maxillary palp with 5 articles (Fig. 16A). ***Thermobia aegyptia*ca**.
— Maxillary palp with 6 articles, the 2 apical ones resulting from a division of the last article by a suture into 2 pieces (Fig. 16B). ***Thermobia domesti*ca**.

### Subfamily Acrotelsatinae Mendes, 1991 *Acrotelsa collaris* (Fabricius, 1793)

**Diagnosis.** Body length up to 18 mm. Antennae are usually about as long as the body length when intact, and terminal filaments are slightly shorter. Body fusiform; thorax wider than the abdomen. The epidermic pigment is whitish or yellowish. Dorsal scales are dark grayish or blackish, except for some transverse bands of whitish scales on the hind margin of some segments usually one broad band on the pronotum and one on the urotergite IX that continues on the anterior part of the urotergite X; the lateral margins of this tergite also bear whitish scales. Macrochaetae are feathered, and the larger ones have pectinations surrounding all the contours of the chaeta. Scales on the head lobulate. Scales on the body surface orbicular, with thin rays. Antennae and terminal filaments lack scales. Maxillary and labial palps are covered with scales, except for their apical article. All articles of legs and styli are also covered with scales. The shape of scales covering appendages is similar to those of the body but usually smaller. Frons with 2 + 2 longitudinally elongate groups (tufts) of macrochaetae and 1 + 1 additional ones close to eyes; clypeus with isolated 1 + 1 large tufts placed next to frons and labrum with setae that are not arranged in tufts. Apical article of labial palp with 3 + 2 sensory papillae. Anterior margin of the pronotum with 1 + 1 tufts of macrochaetae. Lateral margins of the thoracic nota with small combs of macrochaetae. Hind margin of thoracic nota without setae. Trichobothrial areas are open and associated with lateral combs. Thoracic sternites are covered by the basal part of coxae; the prosternum is very reduced, with a medial tuft of setae, mesosternum, and metasternum only with marginal setae. All articles of legs have several transverse combs in their dorsal (anterior) and ventral (posterior) margins: on tarsi, dorsal and ventral combs are connected to form transverse rows of macrochaetae. Urotergite I with 1 + 1 sublateral combs of macrochaetae, urotergites II–VII with 3 + 3 combs, urotergite VIII with 2 + 2 combs, urotergite IX bare. The last abdominal tergite is triangular, with an acute apex and several combs (7–9 in adults) on each lateral side. Urosternites II–VII with 2 + 2 lateral combs of macrochaetae. Two pairs of abdominal styli, on segments VIII and IX, bearing several transverse combs of macrochaetae. Males with small paramera, thin, and tubuliform. Ovipositor short, with about 20 divisions, not exceeding the apex of the inner process of the ninth coxite; gonapophyses IX with strongly sclerotized teeth apically.

**Morphological remarks.** A reasonably good description of this species was provided by Stach (1935) and, more recently, by Hazra et al. (2022a), although some characters (mainly related to scale shape and distribution) have been added in the present diagnosis. The dorsal pattern of scales of this species is characteristic, as well as the shape of the 10th urotergite. No other silverfish with this combination of scale color pattern and shape of the last abdominal tergite is synanthropic in tropical and subtropical areas, although it is likely that these characters could be shared with some free-living species (the scale color pattern is probably variable, and some genera of Lepismatidae show a 10th urotergite with a similar shape). Therefore, the generic and specific identity should be confirmed with a study of other diagnostic characters, such as its chaetotaxy, the small parameres of males, or the secondary ovipositor of females (Supplementary material 2; Supplementary Fig. S23). In this work, the shape of cephalic scales (Supplementary material 2; Supplementary Fig. S5) and the presence of scales on its appendages (only absent in antennae and caudal filaments) are described for the first time. Those of palps are illustrated in Fig. 4 and those of tarsi in Supplementary Fig. S6 (Supplementary material 2). Other genera of Lepismatidae with triangular acute 10th urotergite are *Acrotelsella*, *Stylifera*, *Qantelsella*, and *Hemitelsella*, all of them included in the subfamily Ctenolepismatinae; they lack paramera and do not have the special type of feathered macrochaetae observed in *Acrotelsa* (Supplementary material 2; Supplementary Fig. S2). Currently, the genus *Acrotelsella* is under revision and will most likely be split into several genera. Most of the species belonging to these genera occur in tropical areas and/or in the southern hemisphere, and their presence in peridomestic habitats is not frequent, but confusion with *Acrotelsa collaris* could happen in some cases if not carefully examined.

**Fig. 17.**
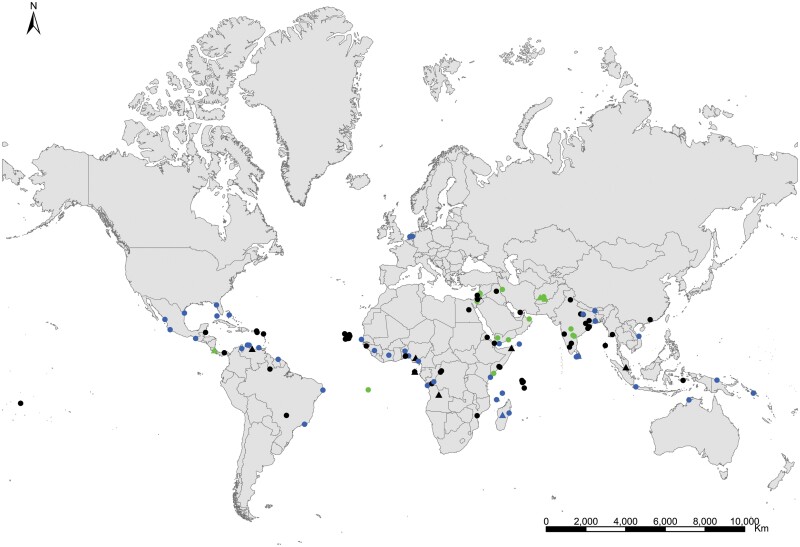
Distribution map of *Acrotelsa collaris*. Triangles (▲) indicate records with nonaccurate locations, and circles (⚫) correspond to records with accurate location coordinates. Records are classified as without habitat information (black), synanthropic habitat (blue), and natural habitat (green). We have designated with a those records where the specific identification of the silverfish cannot be assured based on the available information.

**Geographic distribution and origin** (Fig. 17). This species seems to be pantropical. It has been recorded as synanthropic over tropical and subtropical areas of several continents. Nevertheless, its geographic origin is not clear since some records do not indicate if these insects are collected in natural or domestic habitats. It could have been introduced (and naturalized subsequently) in some Atlantic islands where this species has been found in natural habitats, such as the lava fields of Ascension Island. Several records in natural habitat correspond to Syria, Palestine, Afghanistan, Yemen, Somalia, and India (Supplementary Material 3). This allows us to have an idea about its distribution as a free-living form: *Acrotelsa collaris* could be indigenous in the areas surrounding the Indian Ocean to the west and north, i.e., the Middle East, southern central Asia, Eastern Africa close to the Red Sea, and the Arabian Peninsula.

Subfamily **Lepismatinae** Mendes, 1991

### *Lepisma saccharinum* Linnaeus, 1758

**Diagnosis.** Body length up to 11 mm in adults, usually 7–10 mm. Antennae are shorter than body length but usually longer than half the body length. Terminal filaments are about as long as half the body length. Body fusiform, thorax a bit wider than abdomen base; abdomen narrowing posteriorly. The dorsal surface is covered with silvery gray scales that can be grayish brown or blackish brown in some specimens. Ventral scales are hyaline to light yellowish or grayish, always with a less intense pigment than dorsally. Epidermic pigment is absent or diffuse white to light yellowish. Macrochaetae are smooth, apically bifid. Most scales covering the body are rounded orbicular. Rounded scales also cover the scapus and coxae, which are absent from the remaining articles of the legs and from the remaining appendages. Frons and clypeus with irregular rows of setae that are not concentrated in tufts. The frontal margin is continually setose, without interruption in the middle. Antennae with basiconic sensilla type E. Labial palp with the apical article widened distally, bearing 5 papillae arranged in 2 rows (3 + 2). Anterior margin of pronotum without a collar of macrochaetae. Macrochaetae of the lateral margins of nota isolated, not arranged in combs. Anterior trichobothrial areas of nota open, posterior ones closed. Posterior margins of nota without macrochaetae, only with 1 + 1 minute setae. Prosternum and mesosternum subtriangular; the prosternum with 2 + 2 combs of macrochaetae. Mesosternum and metasternum with 1 + 1 bristle-combs; the mesosternum is bigger and has a more rounded apex than the other 2 thoracic sternites. Urotergites I and IX with only 1 + 1 small groups of macrochaetae in the infralateral position, each one consisting of 1 or 2 strong macrochaetae and another one thinner and shorter that sometimes is absent. Urotergites II–VIII with 1 + 1 infralateral groups and 3 + 3 isolated macrochaetae (1 + 1 on lateral, 1 + 1 on sublateral, and 1 + 1 on sublateral position); the infralateral groups consist of 2 macrochaetae and one thinner outer seta. Urotergite X is almost twice as long as urotergite IX, trapezoidal (cut off straight at the hind margin, or slightly convex or concave), with the posterior corners slightly rounded and bearing 1 + 1 small combs or groups of macrochaetae; its length usually 0.7–0.9 times than its width at the base. Urosternites I–VII (females) or I–VIII (males) with one median comb of about 5–10 macrochaetae; the comb on urosternite I is very small, with 2–5 macrochaetae, inserted on a small projection of the hind margin of the sternite. Urosternites III–VIII with lateral combs of 4 or more macrochaetae (in young specimens and in coxites VIII of females, there can be 2 or 3 only). Parameres of adult males are hyperdeveloped, with a length similar to or greater than the inner process of the coxite IX and with an inner glandular field with 20–50 glandular vesicles. Two pairs of abdominal styli on abdominal segments VIII and IX. Ovipositor short, with 20–25 divisions, its apex surpasses the apex of coxites IX by a length that usually is not higher than the length of the inner process of coxites IX.

**Morphological remarks.** The most appropriate description of this species in the literature was made by Wygodzinsky (1941), and Mendes (1988) gave an update including several microscopic characters. Only by combining the details presented in these 2 descriptions, we can obtain a conclusive identification of this insect. The diagnosis presented in this work includes for the first-time additional details on the scale cover of appendages and an SEM photograph of this character (Fig. 8A), together with another photograph of a male paramera (Fig. 9). Linnaeus originally described this species with few words that can fit with any grayish Lepismatidae (Linnaeus, 1758). However, the drawings presented by Guérin-Méneville (1844) and Lubbock (1873) under the name *Lepisma saccharina* show a long trapezoidal last abdominal tergite (Fig. 2C). Since then, this character has been the reference to identify this species, because it is not shared with any other synanthropic silverfish. Escherich (1905) included an illustration of this species in a dorsal view that can fit with the original description of Linnaeus but especially matches with the aforementioned drawings of Guérin-Méneville and Lubbock. However, *Lepisma saccharinum* has been confused with other common synanthropic silverfish, especially with *Ctenolepisma longicaudatum* (Supplementary Material 4). However, this confusion reveals inaccurate examination of specimens since there are a lot of morphological differences between *L. saccharinum* and the remaining synanthropic species of Lepismatidae. In fact, the genus *Lepisma* is the only domestic silverfish belonging to the subfamily Lepismatinae. Molero-Baltanás et al. (2014) illustrated in vivo differences (dispersed macrochaetae of the frons and long trapezoidal last urotergite) between *L. saccharinum* and *C. longicaudatum*. Thomsen et al. (2019) also illustrated several differences between both species. Additional traits that can be used to distinguish between *Lepisma* and Ctenolepismatinae are (i) the absence of pronotal collar (a fringe of macrochaetae on the anterior margin of the pronotum) in *L. saccharinum* (Fig. 7A); (ii) the hyperdeveloped paramera of males of *L. saccharinum* (Fig. 9) absent in the genus *Ctenolepisma* and related genera; (iii) femora of *Lepisma* only covered by setae, lacking scales on this article of legs (a character never mentioned before; see Fig. 8A), while *Ctenolepisma* and *Thermobia* have scales covering all the femur or a part of its surface (Fig. 8B–D). Several of these characters can be detected in most photographs in dorsal view. Therefore, *Lepisma saccharinum* can be distinguished from other synanthropic silverfish more easily than the remaining ones. Nevertheless, confusion is possible (especially in dorsal view) with some free-living and frequently ant-associated silverfish from the Western Mediterranean and Macaronesian regions belonging to the genus *Neoasterolepisma*  Mendes, 1988. This genus is under revision, and it is likely that it could be joined to the previously established genus *Tricholepisma* or could be split into several genera. If information on habitat is not available, a more detailed microscopic examination is advisable for reliable identification. *Neoasterolepisma* and *Tricholepisma* species have smaller paramera, a special type of sensillum in antennae (asteriform), and a different pattern of trichobothrial areas. The literature on *Lepisma saccharinum* is very extensive, although several incorrect identifications and descriptive errors should be assessed, especially those that are more recent (Bai et al. 2020, Querner et al. 2022; see Supplementary Material 4). Some morphological studies have been published to describe its types of antennal sensilla (Adel 1984, Hädicke et al. 2016), its biology and its internal anatomy: for example, Sahrhage (1954), Pohl (1957), etc. The name “common silverfish” has been used for *L. saccharinum* in some papers, but in most parts of its geographical range, it seems that *C. longicaudatum* is becoming more frequent. In fact, some publications use the name “common silverfish” for *C. longicaudatum*; for example, Swan (1941). Since common names could lead to misinterpretations, the scientific name should be used to refer to this (and all) species of Lepismatidae.

**Fig. 18.**
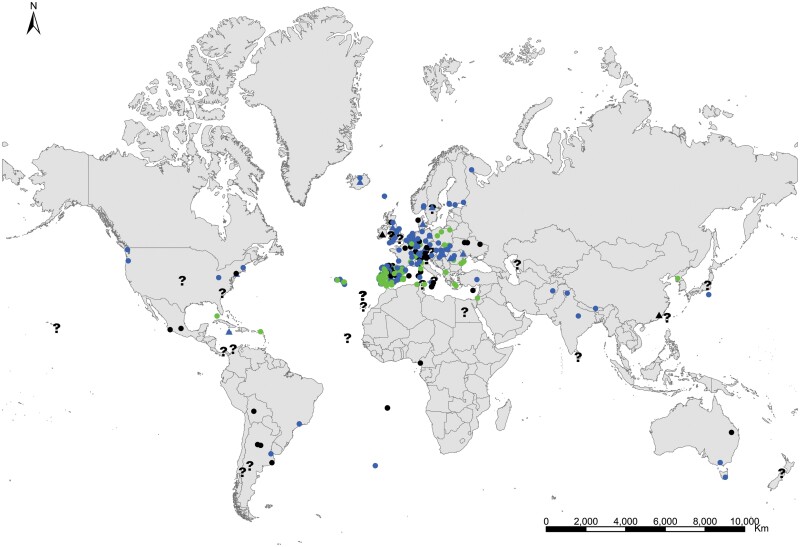
Distribution map of *Lepisma saccharinum*. Triangles (▲) indicate records with nonaccurate locations, and circles (⚫) correspond to records with accurate location coordinates. Records are classified as without habitat information (black), synanthropic habitat (blue), and natural habitat (green). We have designated with a (**?**) those records where the specific identification of the silverfish cannot be assured based on the available information.

**Geographic distribution and origin** (Fig. 18). Linnaeus (1758) described this species as indicating «*habitat in America inter saccharum et utensilia domestica, inde per Europam vulgaris*» (i.e., living in America in sugar and household utensils, becoming common throughout Europe). He assumed that *Lepisma saccharinum* was an American species and then transported to Europe. Actually, neither *Lepisma saccharinum* nor other *Lepisma* species have been found in natural habitats in America, but they do occur in southern and central Europe. Robla et al. (2023) show a map with records of the natural habitats of this species in the Mediterranean region. This suggests that *Lepisma saccharinum* is native to this area. For example, records from several European countries (from Belgium and Portugal to Poland and Turkey; see Supplementary Material 3) indicate that this species is a facultative synanthropic species that can be found both inside and outside houses in southern and central Europe. Free-living forms have been found in chestnut, pine or oak forests and *Eucalyptus* plantations as well as shrublands, from coastal areas to mountainous environments, under stones, rocks, bark of different trees, leaf litter, and pine cones, and sometimes they are found living with ants (e.g., inside *Aphaenogaster*, *Messor*, *Lasius*, *Tapinoma*, and *Tetramorium* nests); see, for example, Molero-Baltanás et al. (2014) and Claus et al. (2022). In the rest of the world where *L. saccharinum* has been reported, this species occurs as an obligate synanthropic (Supplementary Material 3). Its geographic expansion has been very ancient, and its current status is difficult to assess considering the high number of misidentification records. It is possible that some ancient records of this species in several regions, including Europe and North America, do not correspond to the genus *Lepisma* but to some species of *Ctenolepisma*, such as *C. longicaudatum*, because, before the monograph of Escherich (1905), entomologists did not have appropriate reference work for distinguishing species of Zygentoma. See additional details in Supplementary Material 4.

### Subfamily Ctenolepismatinae Mendes, 1991 *Ctenolepisma calvum* (Ritter, 1910)

Figs. 19–23

**Fig. 19.**
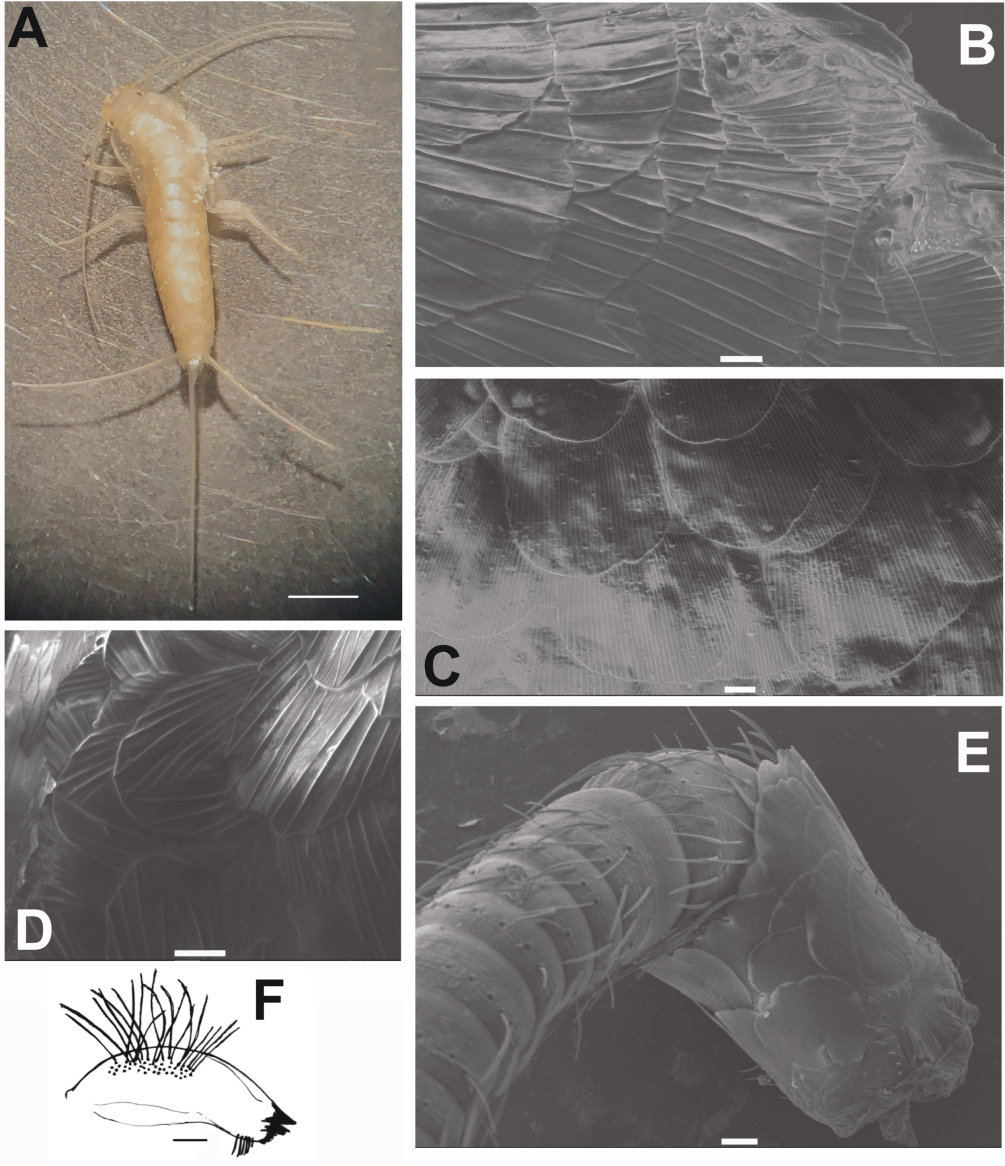
*Ctenolepisma calvum*, redescription based on specimens from Córdoba (Spain) and Prague (Czechia). A) Specimen preserved in alcohol. B) Dorsal scales of the side of the mesonotum. C) Ventral scales on an abdominal sternite. D) Dorsal scales with convergent rays on the anterolateral corner of the pronotum. E) Basal part of the antenna, showing the scapus covered by scales and the pedicel lacking scales. F) Mandible. Scales: A) 2 mm. B–E) 20 µm. F) 0.1 mm.

**Fig. 20.**
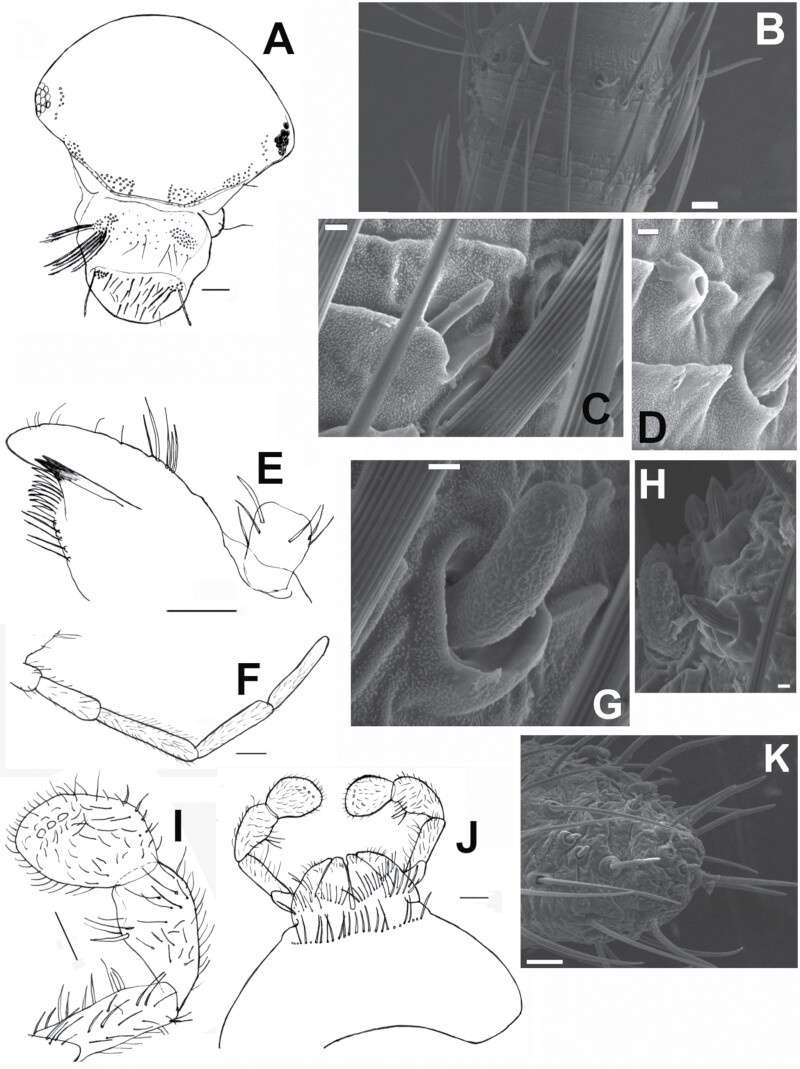
*Ctenolepisma calvum*, redescription based on specimens from Córdoba (Spain) and Prague (Czechia). A) Head showing cephalic chaetotaxy, including clypeus and labrum. B) T-flagellomere of the antenna. C) Sensillum basiconic A. D) Sensillum coeloconic. E) Maxilla, showing galea and lacinia. F). Maxillary palp. G). Sensillum basiconic C. H) Sensillum basiconic B. I) Labial palp showing 3 papillae in the apical article. J) Labium. K) Apex of the apical article of the maxillary palp showing sensilla basiconic B and C. Scales: A, E, F, I, and J) 0.1 mm. B and K) 10 µm. C, D, G, and H) 1 µm.

**Fig. 21.**
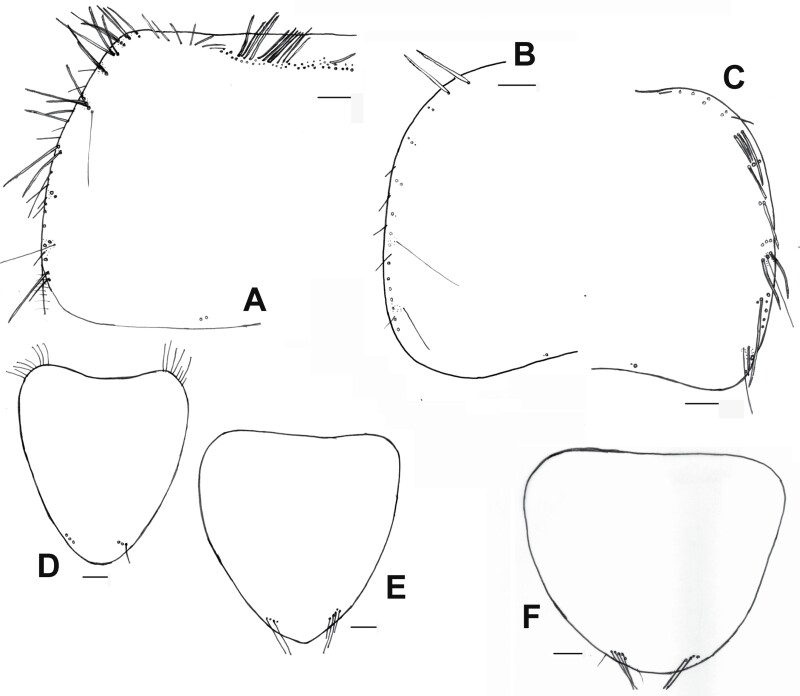
*Ctenolepisma calvum*, redescription based on specimens from Córdoba (Spain) and Prague (Czechia). A) Pronotum. B) Mesonotum. C) Metanotum. D) Prosternum. E) Mesosternum. F). Metasternum. Scales: 0.1 mm.

**Fig. 22.**
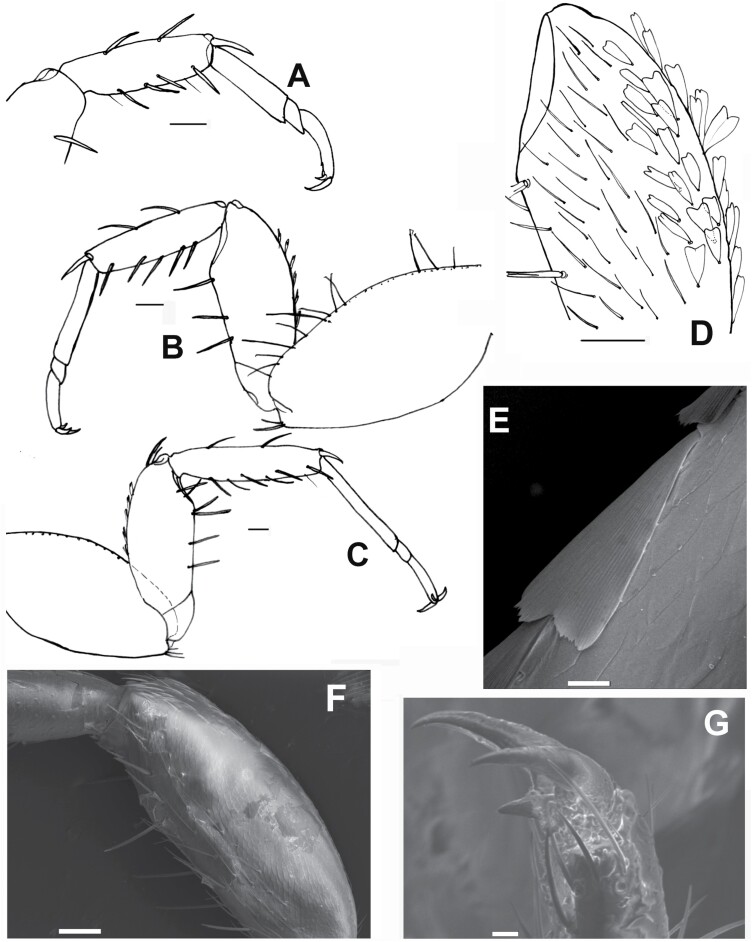
*Ctenolepisma calvum*, redescription based on specimens from Córdoba (Spain) and Prague (Czechia). A) Protibia. B) Second leg. C) Hind leg. D) Cover of scales and setae of the inner (ventral) side of a femur. E) Detail of a femoral scale, SEM photograph. F) Outer (dorsal) side of a leg showing the femur devoid of scales, SEM photograph. G) Pretarsus, SEM photograph. Scales: A–D, and F) 0.1 mm. E and G) 10 µm.

**Fig. 23.**
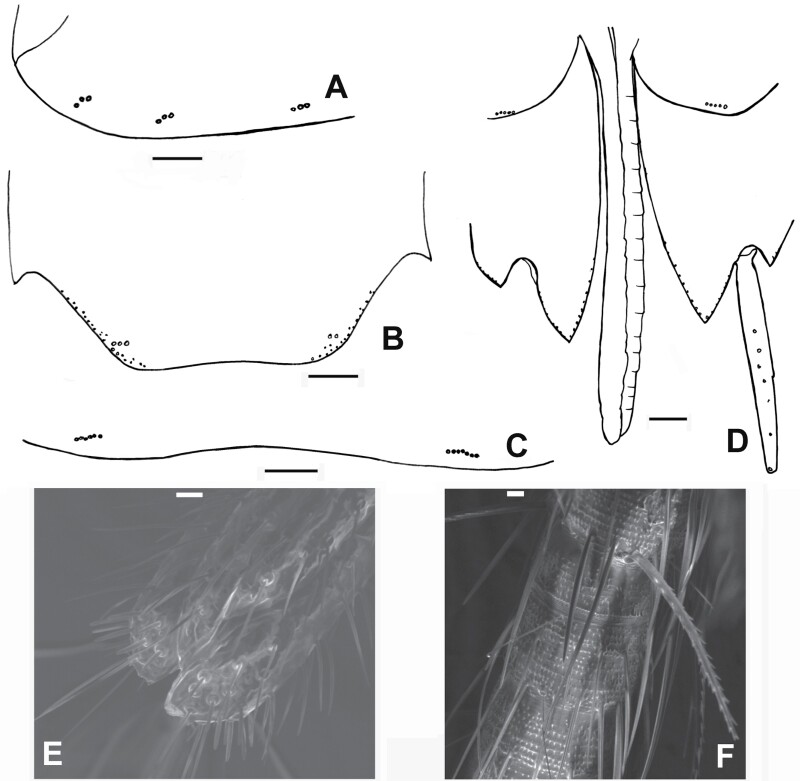
*Ctenolepisma calvum*, redescription based on specimens from Córdoba (Spain) and Prague (Czechia). A) Chaetotaxy of one side of the urotergite III, showing the combs of macrochaetae (only insertions are represented). B) Urotergite X. C) Urosternite IV, hing margin showing the lateral combs (only insertions are represented). D) Ovipositor with coxites IX and styli IX; the hind margin of coxites VIII is also shown. E) Apex of gonapophyses, SEM photograph. F) Some basal divisions of the cercus were photographed with SEM. Scales: A–D) 0.1 mm. E and F) 10 µm.

**Diagnosis.** Body length up to 12 mm, usually 8–9 mm. Macrochaetae plumose. The epidermic pigment is whitish to light yellowish. Dorsal scales are transparent to light grayish, heterogeneous in shape and rib spacing, frequently with widely spaced ribs. Coxae has scales similar to those of the body, and the inner side of the femora is partially covered with subtriangular truncate scales. Tibiae, tarsi, and remaining appendages lack scales. Antennae and terminal filaments are usually not longer than body length. Cephalic chaetotaxy typical of *Ctenolepisma*, with 1 + 1 tufts of macrochaetae on clypeus, 1 + 1 tufts on labrum, and the frontal margin with tufts but not continuous, interrupted in the middle. Apical article of the labial palp with only 3 sensory papillae. Pronotum with setal collar. Thoracic nota with 5–8 pairs of lateral combs and with 1 + 1 posterior combs that can be reduced to one isolated macrochaeta but sometimes bear 2–3 macrochaetae each. Anterior trichobothrial areas of the pronotum located next to the comb N-3 (the anterior to the antepenultimate lateral comb); anterior trichobothrial areas of the mesonotum located next to the comb N-2 (antepenultimate lateral). Each thoracic sternite with 1 + 1 subapical combs of macrochaetae. Abdominal tergite I with 1 + 1 combs, II–V with 3 + 3 combs, VI–VIII with 2 + 2 combs, urotergite IX bare; abdominal sternites lacking median combs and with 1 + 1 lateral combs on segments III–VIII. Abdominal combs with very few macrochaetae, the infralateral ones usually with 3–4 macrochaetae and the others with 2–3 each or even reduced to 1–3 macrochaetae; this reduction is frequent in sublateral combs. Urotergite X trapezoidal, short (about 0.25–0.3 times longer than wide at its base), with a straight or slightly concave hind margin. Only one pair of abdominal styli on segment IX. The ovipositor is short, with about 20 divisions; its apex does not clearly surpass the tip of the styli.

**Examined material. SPAIN:** Córdoba: 4 ♀, basement of a house in the city of Córdoba, 7-XI-2021 and 31-X-2023 (M. Gaju *leg*. and. UCO, Ref 2764 *dep.*). This is the first reliable record for Spain.

**Additional examined material. CZECHIA:** Prage. 6 females, 2021, (M. Kulma *leg.* and UCO, Ref. 2868 *dep.*). Specimens provided by Martin Kulma (National Institute of Public Health, Prague, Czechia). **SRI LANKA**: Radanaputra: 1 female, former “Ceylon,” V-1907 (no *leg.* information; offered to Luis F. Mendes by Professor S.L. Tuxen (Copenhagen Museum) for study, firstly mounted on a slide and deposited in the collection of Centro de Zoologia (IICT, Lisbon), now transferred to the collection of the Museu Nacional de História Natural, Entomology, Lisbon, Portugal). One specimen from Córdoba and one from Prague will be transferred to MNCN collection. This represents the first reliable record of this species for Spain.

**Redescription.** Medium-size silverfish. The thorax is slightly wider than the abdomen, which tapers slightly posteriorly from the fifth abdominal segment. Live specimens are very light colored, dorsally grayish white or yellowish-white, ventrally whitish or light yellowish; (photographs in Kulma et al. 2022, Querner et al. 2022, Shimada et al. 2022), but some specimens are slightly more grayish. Antennae and terminal filaments are almost hyaline, with a very faint yellowish-white tonality. In alcohol, specimens become light brownish or yellowish (Fig. 19A).

**Body size.** Spanish specimens can grow up to 10 mm, but specimens from Japan maintained in the laboratory can reach 12 mm in length (Shimada et al. 2022); head width of up to 1.2 mm; thorax length of up to 3 mm; thorax width up to 2.1 mm (at mesothorax level). Antennae shorter than body length, when intact up to 7.5 mm. Terminal filaments are shorter than the antennae; cerci are up to 5.5 mm, and paracercus are up to 7 mm, but they are frequently broken.

**Pigmentation and scales.** The epidermic pigment is light yellowish or yellowish-white, almost absent in some regions of the body. Appendages are almost hyaline; the most intensely pigmented areas with yellowish color are the head and the 4–5 last abdominal segments. The gut content is frequently visible by transparency. Macrochaetae are plumose as in other *Ctenolepisma* species, with abundant pectinations, hyaline to yellowish, those of the frons and clypeus more intensely pigmented.

Scales cover the body dorsally and ventrally; they are hyaline or with faint grayish-white pigment, almost transparent, and hardly visible with a stereomicroscope or light microscope unless they shine slightly due to the lateral incidence of light. Most of them are rounded or subrectangular and show some subparallel ribs that do not surpass the posterior margin of the scale; this is frequently irregular or denticulated. The ribs of dorsal scales, especially those on the thorax, are very widely spaced and not very numerous compared with other synanthropic species of *Ctenolepisma*: a typical scale on the thoracic nota of *C. calvum* has only 12–15 ribs, which contrast with the 30–40 ribs of a ventral scale. When examined by SEM, these scales have a greasy appearance, with a waxy sheen that is not observed in other *Ctenolepisma* species (Figs. 13B and 19B; see also Fig. 3A and B in Shimada et al. 2022). Ventral scales have more numerous and dense ribs (Fig. 19C). Some smaller submarginal scales on the pronotum have ribs that converge in the distal margin (Fig. 19D). The appendages are devoid of scales, with the exception of the scapus of the antennae and all coxae; on femora, only a few rows of modified scales are present in the outer margin of the article (see below). Scales of the scapus are slightly different from those of the body because they have more dense but less pronounced ribs (about 50–60, see Fig. 19E).

**Head.** Wider than long (Fig. 20A) covered with scales except for clypeus and labrum. Chaetotaxy is similar to that observed in other *Ctenolepisma* species, but macrochaetae are reduced in number. The 1 + 1 frontal tufts have about 40 macrochaetae arranged in irregular rows (the row closer to the median gap has 6 macrochaetae). The median gap between these frontal brushes is about as wide as the width of the brushes. There are 1 + 1 rows of about 8–10 periocular macrochaetae surrounding the inner margin of the eyes (one of them isolated at the upper part of the eye) and 1 + 1 small frontolateral tufts of about 20 macrochaetae between the frontal tufts and the periocular ones, separated from both by a narrow gap. Some of the macrochaetae of these frontolateral tufts are arranged in a single row in part close to the eyes. An isolated group of 2–3 macrochaetae is placed above each frontolateral tuft. Clypeus with 1 + 1 tufts of about 35 macrochaetae; there are some thin and long acute setae dispersed on the median area. Labrum with 1 + 1 small tufts of macrochaetae connected by a transverse and irregular fringe of thin acute setae.

**Antenna.** The scape of the antenna is about twice longer than the pedicel, covered with rounded orbicular scales that bear dense parallel ribs; only a ring of smooth setae is visible subapically. Pedicel also with a subapical ring of setae and some additional setae inserted in 2 groups on the inner and outer sides of this segment of the antenna (Fig. 19E). The flagellum has a lot of annuli. All of them bear 1 or 2 rows of setae (chaetic sensilla), and some of them (T-annuli) bear trichobothria. Trichoid, coeloconic, campaniform, and basiconic sensilla are also present (Fig. 20B–D, G, and H); 3 types of basiconic sensilla can be distinguished, similar to types A, B, and C described by Adel (1984) for *Thermobia domestica*.

**Mouthparts**. Mandibles (Fig. 19F) as in other *Ctenolepisma*, with a group of about 10 strong and short, pigmented, and apically bifurcated setae and a large bush of about 55–60 macrochaetae externally. Maxilla (Fig. 20E) with 6–7 smooth short setae externally proximal to the palp, the galea with 5–6 smooth and pointed setae in its basal half and a few cilia distally; lacinia with 2 strong teeth, followed by about 7 lamellate processes and a row of 5 thin setae. The apical article of the maxillary palp is slender (Fig. 20F), about 7 times longer than wide, as long as the penultimate article, and slightly shorter than the antepenultimate. The apex of the apical article has several basiconic sensilla of types B and C, similar to those present in the antennal flagellum (Fig. 20K). Labium wider than long, with a transverse row of setae in the distal part of the prementum and some groups of setae on glossae and paraglossae, mainly forming an oblique fringe; some of these setae are apically bifurcate (Fig. 20J). Labial palp with oval apical article, slightly widened medially, 1.15 times longer than wide and with a row of 3 papillae arranged in a row (Fig. 20I). Penultimate article as long or 1.1 times longer than the apical article.

**Thorax.** Thoracic nota with an irregular row of macrochaetae inserted on their lateral margin. The number of lateral combs is difficult to count because some of them are reduced to one macrochaetae or even none, and only the marginal macrochaeta remains. There are 1–4 isolated macrochaetae inserted on the anterolateral corner that can be interpreted as reduced combs. Pronotum (Fig. 21A) with a setal collar composed of 2–3 irregular rows of strongly pectinate macrochaetae. Anterolateral row with several dispersed smooth and bifid setae. Lateral margins with about 8 combs of 1–4 macrochaetae, the 4 anterior ones very close to the anterior angle of the pronotum. The anterior trichobothrial areas are associated with the N-3 comb (anterior to antepenultimate), and the posterior trichobothrial areas are associated with the N comb (last lateral comb). When these combs are reduced and not distinguishable, the anterior trichobothria is located at about 0.28, the total length of the lateral margin, and the posterior one is about 0.72 along this margin. Both trichobothria are located on the inner part of the trichobothrial area. Posterior margin with 1 + 1 combs of 1–3 pectinate macrochaetae. Mesonotum (Fig. 21B) with 7–8 + 7–8 lateral combs and 2 isolated additional setae on the anterolateral corner. Each lateral comb has 1–3 macrochaetae, and the trichobothrial areas are inserted in the antepenultimate (N-2) and last (N) comb of the notum. When these combs are reduced and not clearly distinguishable, the anterior trichobothria are located at about 0.52 along the length of the lateral margin, and the posterior ones are at about 0.77 this margin. The 1 + 1 combs of the posterior margin have 1–2 macrochaetae. Metanotum (Fig. 21C) with 5–8  + 5–8 lateral combs of 1–3 macrochaetae each and 1–3 isolated additional setae on the anterolateral corners. In most specimens examined, the 2 posterior lateral combs on each side (N and N-1) are reduced, and no macrochaetae are visible apart from the marginal one. When they consist of 1–2 combs, the anterior and posterior trichobothrial areas are associated with them. If lateral combs are not clearly distinguishable, the anterior trichobothria are located about 0.65 along the length of the lateral margin, and the posterior ones are about 0.84 this margin. Posterior margin with 1 + 1 combs of with 1–2 macrochaetae; if there is a pair of macrochaetae, one of them is larger than the other (even its insertion). Presternum wide, with a transverse row of setae. Prosternum (Fig. 21D) subtriangular, with rounded-elliptical posterior margin, slightly longer than wide (ratio length/width about 1.12); anterolateral corners with several thin setae and marginal setae only in the apical part; with 1 + 1 subapical groups of 3–4 macrochaetae that are not arranged in a straight row. Mesosternum (Fig. 21E) with a similar shape and ratio length/width, but a little larger (width and length about 15% higher than those of the pronotum), with 1 + 1 subapical oblique combs with 4–5 macrochaetae. Metasternum (Fig. 21F) is wider than long, its ratio length/width about 0.87, heart-shaped with the posterior margin broadly convex, slightly truncate or straight in the apical area. It has 1 + 1 subapical oblique combs of 4–6 macrochaetae; the distance between these combs is about 4 times the width of a comb.

**Legs.** As illustrated in Fig. 22A–C. PIII is larger than PII and PII larger than PI; metatibiae is about 1.3–1.4 times longer than mesotibiae and 1.6 times longer than protibiae. Coxae with a row of macrochaetae in the external margin and some thin spaced setae on the inner margin, and about 4 strong macrochaetae over the articulation; this article is covered with orbicular, rounded scales similar to those of the scapus and the ventral part of the insect, i.e., with a lot of dense and only slightly pronounced parallel ribs. Trochanter with some scattered thin setae. The femur is covered with setae on the inner side, except from 3 to 4 rows of modified scales on the dorsal/external side of the article (Fig. 22D). These scales are subtriangular, of variable width but always longer than wide, with the distal part truncate or concave and some ribs slightly marked (Fig. 22E). On the outer side, the ventral/internal part is covered with thin setae, and the rest of the surface is devoid of setae, where the tegument relief is scaly when observed with SEM (Fig. 22F). Four strong macrochaetae are inserted on the ventral/internal side. Protibia is about 3.3–3.4 times longer than its width. This ratio length/width is about 3.8–3.9 in the mesotibia and about 4.4–4.5 in the metatibia. Tibiae are covered only with setae; scales are absent. There are 2 robust macrochaetae on the dorsal/external margin and 3–5 on the ventral/internal margin (usually 3 on the protibia, 4 on the mesotibiae, and 5 on the metatibia). The length of these macrochaetae is similar to the width of the article on the mesotibiae and slightly shorter on the metatibiae. The apical part of the tibiae has a strong macrochaeta and 2 additional thin macrochaetae ventrally. The tibial spur is more developed in PI and PII, and the protibia is 3.7 times longer than its apical sur, the mesotibia about 4.1 times, and the metatibia about 6.7 times. Tarsi 1.2 times longer than the corresponding tibia, consisting of 4 articles. The basal tarsal article (tarsomere 1) is about 0.7, 0.75, and 0.8 times longer than the tibia and about 57%, 62%, and 66% of the length of the tarsus (these metrics correspond respectively to PI, PII, and PIII). The second tarsomere is similar in length to the third tarsomere, and the fourth tarsomere is longer than the third. The joint between the first and second tarsomere is almost straight, but the one between the second and the third is very oblique. Tarsomere 1 of PI and PII with 2 ventral rows of slender spines that are absent in the PIII. Pretarsus with 2 lateral claws and a smaller median empodium (Fig. 22G).

**Abdomen.** In some dissected specimens, the abdominal chaetotaxy is difficult to discern because of the presence in the slides of numerous drops of fat coming from the gut contents, probably related to the diet of the silverfish. Urotergite I with 1 + 1 lateral combs of 2 macrochaetae each, urotergites II–V with 3 + 3 combs of macrochaetae as in Table 2 (see also Fig. 23A and Fig. 5 in Kulma et al. 2022), urotergites VI–VIII with 2 + 2 combs, and urotergite IX lacks setae. The infralateral combs usually consist of 3–4 macrochaetae, the lateral combs consist of 2–3 macrochaetae, and the submedian combs usually consist of 1 or 2 macrochaetae. Urotergite X short trapezoidal (ratio length/width of the trapezoid about 0.25–0.3), with the posterior margin straight or slightly concave, lateral margins with several setae in their posterior part and 1 + 1 subapical combs of 2 macrochaetae (Fig. 23B). Urosternites I and II without setae. Urosternites III–VII with 1 + 1 lateral combs composed of 4–8 macrochaetae each (usually 5 or 6, see Fig. 23C). The distance between these combs ranges from 8 to 18 times the width of a comb. Dorsal and ventral views of the abdomen were shown by Shimada et al. (2022) in Fig. 3D and E of that paper. Each coxite VIII with a comb of 4–5 macrochaetae. The inner process of the coxite IX is about 1.2 times longer than wide at the base and about 4 times longer than the outer process (Fig. 23D). Only one pair of styli. The length of the styli (without the apical spines) is about 2.5, greater than the length of the inner process of the coxite IX (Figs. 14B and 23D). Ovipositor short, with 19–21 divisions, clearly surpassing the apex of the internal processes of coxites IX but barely exceeding the apex of the styli (Figs. 14B and 23D). The apex of the gonapophyses is shown in Fig. 23E. This ventral part is also shown in Fig. 3C of Shimada et al. (2022).

**Table 2.** Abdominal chaetotaxy of *Ctenolepisma calvum*. The variability in the number of macrochaetae per comb is based on Spanish, Sri Lankan, and Czech specimens

Abdominal segment	Urotergal combs			Urosternal combs
	Infralateral (A)	Lateral (B)	Sublateral (C)	Lateral (L)
I	2	–	–	–
II	3–4	2–3	1–2	–
III	3–4	2–3	1–2	4–8
IV	3–4	2–3	1–2	4–8
V	3–4	2–3	1–2	4–8
VI	3–4	1–3	–	4–8
VII	3–4	1–3	–	4–8
VIII	3–4	1–3	–	4
X	2			

**Terminal filaments.** Similar to those of other Ctenolepismatinae, with similar types of setae than described for *Thermobia domestica* by Krantzler and Larink (1980), i.e., large feathered macrochaetae, smooth chaetic sensilla with variable length, trichoid sensilla and trichobothria (Fig. 21F).

**Males**. Unknown.

### Taxonomic Discussion

*Ctenolepisma calvum* was described by Ritter (1910) as *Peliolepisma calva* on the basis of specimens from Colombo (Sri Lanka). This author established a new genus for this species due to the (apparent) rudimentary or absent chaetotaxy in most parts of the thorax and abdomen. According to its description, the abdomen lacks hairs ventrally. As the present redescription indicates, this was incorrect since this species has several dorsal and ventral macrochaetae on the thorax and abdomen. Probably, Ritter was not able to see them with the available optic devices he used because these big setae frequently come off, and only their insertions are visible with a light microscope (Kulma et al. 2022). The specific name “calvum” (= without hairs) can be only justified because the setae of these parts of the body are reduced (but not absent) compared with related species. The statement of the keys of Aak (2019) and that given by Querner et al. (2022) in their Supplementary Table S3, indicating “abdominal, dorsal plates without visible bristle combs” and “abdomen without hairs,” respectively, are incorrect and should be replaced by “dorsal plates of the abdomen with combs consisting of few dorsal macrochaetae, often not clearly visible.” Moreover, the aforementioned key of Querner et al. (op. cit.) is in contradiction with the character shown in their Supplementary Table S4, where they indicate the presence of 3 + 3 combs in urotergites II–V and 2 + 2 combs in urotergites VI–VIII (in this case, correctly). The problem with the characterization of this species lies in the fact that, after Ritter’s original description, no silverfish specialist redescribed the species. Wygodzinsky (1957) examined some specimens of *C. calvum* from Sri Lanka and had the opportunity of writing a redescription, but he only included the genus *Peliolepisma* in a key to genera of Lepismatidae and commented that “this genus seems to differ from *Ctenolepisma* only in character mentioned in the key; all other features agree so closely that it seems difficult to maintain a generic distinction.” After this comment, experts in Lepismatidae assumed that *Peliolepisma* has the typical chaetotaxy of *Ctenolepisma* with only one exception. This implies that *Peliolepisma calva* has dorsal and ventral macrochaetae in their thorax and abdomen, against the original description of Ritter (*Ctenolepisma* is defined as having at least 3 + 3 bristle-combs in some abdominal tergites and at least 1 + 1 bristle-combs in some abdominal sternites). But Wygodzinsky maintained the genus *Peliolepisma*.

Ten years later, Paclt (1967) made a relevant revision of Lepismatidae and included this species in the genus *Ctenolepisma*, but he gave no reasons to justify this synonymy. Although the reasons were given by Wygodzinsky (1957), this author criticized the synonymy of Paclt (Wygodzinsky 1972) and included in this latter work the species as *Peliolepisma calvum* in a key of Lepismatidae of the Caribbean that incorporated some other synanthropic silverfish found in that area. Wygodzinsky also included a small illustration (Fig. 7B and C, page 13 of that article) of the only character that separates *Peliolepisma* from *Ctenolepisma*: the reduction of the posterior bristle-combs of thoracic nota to only one macrochaetae, while in the remaining *Ctenolepisma* species, these combs are composed by at least 2 (usually more) macrochaetae. In fact, this is not a very important difference, and subsequent works did not consider the genus *Peliolepisma* (e.g., those made by John Irish or Luis F. Mendes). In the present article, we have detected that the number of macrochaetae in this position is variable from 1 to 3 in both the Czech and Spanish populations, so this variability should be included in the keys to identify this species and not indicate only that there are only 1 + 1 “individual bristles” (Querner et al. 2022; Supplementary Table S3). Shimada et al. (2022) and Kulma et al. (2022) show photographs (Figs. 3B and 6, respectively) where 2 macrochaetae are visible in each position of the posterolateral margin of thoracic nota, and not only one. Furthermore, the first author of this work had the opportunity of examining in Lisbon one specimen of *Ctenolepisma calvum* collected in Sri Lanka and mounted on slide by Luis F. Mendes and concluded that this species could be included inside *Ctenolepisma* without problems. The aforementioned specimen is the most reliable reference for Sri Lankan specimens since the status and whereabouts of the types of this species are unknown. The posterior combs of setae of the nota of this specimen were composed of only one macrochaeta, but all the macrochaetae in this position were detached, and only their insertions (sockets) were visible. A second insertion corresponding to a smaller seta was also visible beside the bigger insertion, as designed by Wygodzinsky (1972). The combs of other parts of the body were reduced compared with those of other species of *Ctenolepisma*, but not absent, so this can be considered to deserve a specific but not generic differentiation. In our opinion, *Ctenolepisma calvum* is related to *C. rothschildi*, but with a higher degree of reduction of the number of macrochaetae in bristle-combs.

Differences between *C. calvum* and several related species are presented in Supplementary Material 1. Moreover, some comments on several diagnostic characters of *C. calvum* are given below to avoid misidentifications that are probably widespread in citizen science platforms. Querner et al. (2022) commented that these photographs were identified “by experts,” but in our opinion, these identifications are only tentative at the specific level, and anyone defending that silverfish can be identified at this level only with usual photographs cannot be considered as an expert in Zygentoma.

**General color**. Ritter indicated in his original description of “*Peliolepisma calva*” that this species has a yellowish-white color with light brownish scales. Aak et al. (2019) indicate that this species is shimmering white and one-colored, and its scales are white. Both authors coincide in a light whitish color, but there is some contradiction if we compare both descriptions. The specimens coming from Prague are yellowish white or light grayish white, and when fixed in alcohol, they become light brownish (see Fig. 19A). In some cases, the dorsal color of silverfish comes from their scales. In *C. calvum*, scales are nearly transparent or light grayish. When scales are lost, totally or partially, the epidermic pigmentations become more apparent, and, in this species, it is light yellowish-white. The general appearance of the insect is almost white but not uniform when some scales are lost. This could be a characteristic and diagnostic character of this species, but unfortunately, some other synanthropic species can be variable in their color. *C. longicaudatum* and *C. villosum* (and probably *C. rothschildi* and some other species), although usually bearing darker grayish dorsal scales, change this tonality when molting and/or some specimens have lighter, almost transparent scales. For example, Womersley (1937) describes *C. longicaudatum* specimens observed in Australia as having a whitish cream color without silvery sheen. Moreover, when these insects lose dorsal scales, they become whitish because their epidermic pigment is also of this color, so confusion of *C. calvum* with some other synanthropic species is possible. Some colleagues who purported to have discovered specimens of *C. calvum* in Spain sent us the specimens they collected, but they were in fact, young *C. longicautatum* in some cases and *C. villosum* in some others. In citizen science platforms, some specimens are identified as *C. calvum* with the only criterion of being whitish, which is an unfortunate mistake.

**Length of antennae and caudal appendages.** This character was used in the taxonomy of these insects several decades (or centuries) ago, but it is not important (or only a little) as the diagnostic character to separate species because these appendages are easily broken, even in nonmanipulated living specimens. As happens with body length, differences between intact *Ctenolepisma longicaudatum* and intact specimens of other species are probably significant, but not when comparing *C. calvum*, *C. rothschildi*, or *C. villosum*, among others.

**Thoracic ventral chaetotaxy.** Nothing has been mentioned in any previous description of this species, but we have observed that each thoracic sternite of *Ctenolepisma calvum* has 1 + 1 subapical bristle-combs. This is different in *C. longicaudatum* and *C. villosum*, that bear 2 or 3 pairs of combs in the prosternum (*C. longicaudatum* has also 2–3 pairs in the mesosternum).

**Abdominal dorsal chaetotaxy.** As commented on previously, the mistake in the diagnostic character of *Peliolepisma* has been transmitted to the key of Aak et al. (2019) and to the table and key presented by Querner et al. (2022). Bristle-combs actually exist in the dorsal plates of the abdomen of this species, as in any other *Ctenolepisma*, and their visibility depends on the ability of the observer and the methods used for the observation. Incidental identification can have success when identifying a silverfish belonging to *C. calvum* using Aak’s or Querner’s keys when their bristle-combs are not detected because they are smaller than in other *Ctenolepisma*, but it is incorrect to write that these combs do not exist and very imprecise that “they are not visible” since they exist and could be visible. Frequently, dorsal macrochaetae are also unnoticed on a cursory examination of *C. villosum* or *C. rothschildi*. *Ctenolepisma calvum* bears 1 + 1 bristle-combs in the abdominal tergite I, 3 + 3 bristle-combs in abdominal tergites II–V (Fig. 6), and 2 + 2 bristle-combs in abdominal tergites VI–VIII. The urotergite IX has no combs, and the urotergite X has 1 + 1 bristle-combs. This arrangement is similar to that of *C. villosum* and *C. rothschildi*, but different from that of *C. longicaudatum* or *C. lineatum* (*C. longicaudatum* has 3 + 3 combs in the urotergite VI and *C. lineatum* has 3 + 3 combs in the urotergites VI and VII). Moreover, the number of macrochaetae per comb is higher in *C. villosum* and *C. rothschildi*, although in young specimens of this latter species, this difference is not very clear (one of the synonyms of *C. rothschildi* is *C. reducta*  Folsom, 1923; this specific name refers to the reduction of the number of macrochaetae compared with other *Ctenolepisma* species).

**Abdominal ventral chaetotaxy.** As it happens in most species of the subgenus *Ctenolepisma*, *C. calvum* bears 1 + 1 bristle-combs of several macrochaetae in their abdominal sternites III–VIII (Fig. 7). This is also contrary to Ritter’s original description, who wrote ‘*die Ventralseite des Abdomens ist völlig kahl*’ (i.e., the ventral side of the abdomen is completely bare). The present redescription states that *C. calvum* has small but visible urosternal bristle-combs (1 + 1 in urosternites III–VIII). These ventral bristle-combs are difficult to see in living specimens and even in fixed specimens if the macrochaetae are broken off, but the dissection will confirm their presence: at least their insertions should be visible. This character is useful to separate *C. calvum* from *C. villosum*, which shows one additional median comb on urosternites II–VI (*C. calvum* lacks median urosternal combs). But *C. rothschildi* has the same ventral chaetotaxy as *C. calvum*.

**Number of pairs of abdominal styli.**  Ritter (1910) reported in his original description that his *Peliolepisma calva* has 2 pairs of styli in both sexes, one on abdominal segment VIII and one on segment IX. He also included an illustration to support this (Figs. 2 and 3 in the aforementioned work of Ritter), but in all the specimens examined, the number of pairs of styli is only one (Fig. 14B). In some species of Lepismatidae young specimens have not developed the definitive number of styli. In *C. longicaudatum*, for example, the styli VIII is not developed in young specimens. If the authentic *C. calvum* from Sri Lanka has 2 pairs of styli, all the specimens studied in this work might correspond to a different and undescribed species. However, the specimen from Sri Lanka examined by the first author of the present work has only one pair of styli and does not correspond to a young specimen of *C. longicaudatum*. In our opinion, Ritter illustrated the ventral part of specimens of *C. longicaudatum* that he collected in Colombo together with “*Peliolepisma calva*” and reported in the same paper (Ritter, 1910). He probably mixed in the same sample specimens of both synanthropic species and made a mistake in attributing the illustrations of *C. longicaudatum* to *Peliolepisma calva*; this mistake has not been corrected until now.

**Fig. 24.**
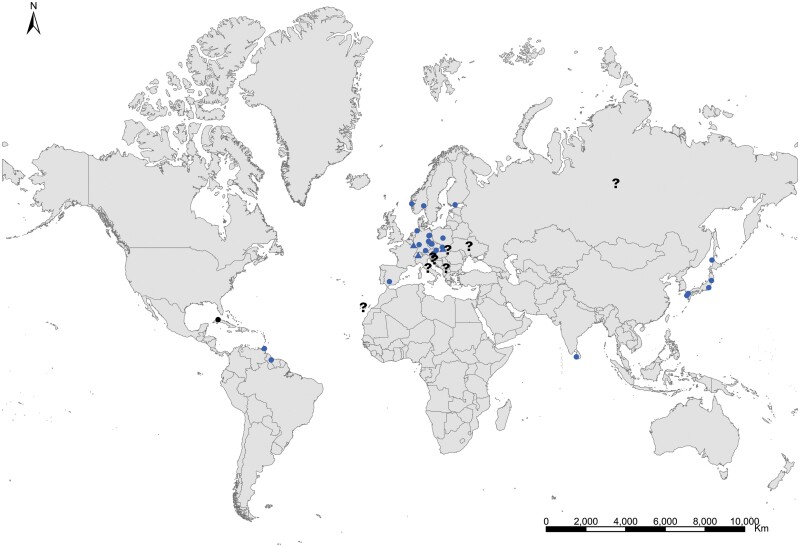
Distribution map of *Ctenolepisma calvum.* Triangles (▲) indicate records with nonaccurate locations, and circles (⚫) correspond to records with accurate location coordinates. Records are classified as without habitat information (black), synanthropic habitat (blue), and natural habitat (green). We have designated with a (**?**) those records where the specific identification of the silverfish cannot be assured based on the available information.

**Geographic distribution and origin** (Fig. 24). *Ctenolepisma calvum* has not been recorded in natural habitats. All records published come from domestic environments, so their geographic origin is uncertain. Nevertheless, considering the geographic distribution of the characters that it shares with other species of *Ctenolepisma* (for example, heterogeneous and wide-spaced dorsal scales or modified scales on femora), it is probable that it has a sub-Saharan African origin. However, the possibility of an East-Palearctic origin cannot be discarded, but it is less likely. Records of this species in Europe and Asian countries different from Sri Lanka are recent, all of them in the last 2 decades, but unfortunately, the reliability of most of them should be considered with caution because most ‘experts’ that identified them did not provide accurate morphological support for their identifications, so at this moment most of them should be considered as doubtful (see Supplementary Material 4). Nevertheless, it is clear that the species is widespread in Central Europe (Kulma et al. 2022, Querner et al. 2022, Bednár et al. 2023), the Caribbean area (Wygodzinsky 1972), and southern and eastern Asia: Ceylon (Ritter 1910, Wygodzinsky, 1957) and Japan (Shimada et al. 2022). It is likely that the species has been introduced in other geographic areas (i.e., Singapore, Venezuela), but an accurate morphological or genetic assessment of the specimens of most countries should be carried out to validate the most inaccurate records. We have checked morphologically some specimens initially identified as *C. calvum* and some of them turned out to be *C. longicaudatum*, and some others turned out to be *C. villosum*. *Ctenolepisma calvum* is apparently increasing its distribution in some countries of Central Europe (Austria, Germany, Czech Republic, or Slovakia). The present record from Spain should be considered the first reliable one in the Mediterranean area.

This species has recently been found to be parthenogenetic (Watanabe et al. 2023), and this has been confirmed in our laboratory: we only found female specimens in Córdoba and, starting from a single female isolated for 1 year in a container, 2 subsequent generations of females have been obtained. This biological particularity, not indicated previously in Lepismatidae but documented for Nicoletiidae (Picchi 1972), has probably favored the expansion of this species since a new population can originate from a single silverfish inadvertently transported.

### *Ctenolepisma lineatum* (Fabricius, 1775)

**Diagnosis.** Body length up to 14 mm. Macrochaetae plumose. Epidermic pigment is light yellowish to brownish, darker on the head, and some appendages. Dorsal scales on the thorax and abdomen usually form a pattern of parallel longitudinal fringes, alternating darker and lighter lines (frequently 4–6 darker lines can be distinguished); the contrast between these lines is variable, and dark lines can be dashed, interrupted to form isolated points. The body is covered by orbicular rounded or subrectangular scales with dense ribs. Coxae have scales similar to those of the body, and the inner side of the femora and tibiae are covered with lanceolate scales, most of them ending in an acute apex. Tarsi, styli, and cerci with some narrow acute lanceolate scales. Antennae, when intact, are longer than body length; caudal filaments are about as long as the body or even longer. Cephalic chaetotaxy is typical of *Ctenolepisma*, with a median gap between the frontal tufts, 1 + 1 clypeal tufts of macrochaetae, and 1 + 1 tufts on the labrum. An apical article of the labial palp with 5 sensory papillae is arranged in a row. Pronotum with setal collar. Thoracic nota with 7–11 pairs of lateral combs and with 1 + 1 posterior combs of 4–8 macrochaetae. Anterior trichobothrial areas of the pronotum are associated with the antepenultimate (N-2) lateral comb; anterior trichobothrial areas of the mesonotum are associated with the penultimate (N-1) comb. Prosternum with truncate hind margin, with 4 + 4 or more small combs of macrochaetae. Mesosternum and metasternum with rounded convex hind margins and 2–3 pairs of combs on their subapical areas (the metasternum usually with 2 pairs, wider and more rounded); macrochaetae of these combs arranged on a single row. Abdominal tergite I with 1 + 1 combs, II–VII with 3 + 3 combs, urotergite VIII with 2 + 2 combs, and IX bare; abdominal sternites lacking median combs and with 1 + 1 lateral combs on segments III–VIII. Dorsal abdominal combs with 4–12 macrochaetae each. Urotergite X subtriangular, short, with convex, rounded to slightly acute hind margin. Both sexes with only 3 pairs of abdominal styli on segments VII–IX; styli on segment VII are developed only in adults longer than 8 mm. Ovipositor long, with 47–52 divisions, its apex clearly surpassing the tip of styli IX by 0.9–2 times their length.

**Morphological remarks.** For a long time, *C. lineatum* was confused with *Ctenolepisma nicoletii* (Lucas, 1846). Recently, the status of *C. nicoletii* as a different species was revalidated (Molero Baltanás et al. 2012). Previous to this taxonomic revision, most of those records published as the “variety” (or subspecies or “aberration”) *Ctenolepisma lineata pilifera* actually correspond to the synanthropic species *C. lineatum*. The common name “four-lined silverfish” has been used for *C. lineatum* in several works. This pattern with 4 longitudinal lines of scales is shared with several species of Lepismatidae like *C. nicoletii*, *C. vieirai* Mendes, 1981, *C. almeriense* Molero-Baltanás, Gaju-Ricart, and Bach de Roca, 2005 and *C. algharbicum*  Mendes, 1978, all of them with 2 pairs of styli when adults (with the exception of some specimens attributed to *C. vieirai* that require revision). These species, distributed mainly in the Macaronesian and western Mediterranean regions, could show 4 or 6 alternative and parallel dark and white lines. Furthermore, the 4-lined pattern is variable; for example, it is not clearly visible in specimens of *C. lineatum* with poor contrast between dark and light scales, especially when the insects are close to their molt, or the dark lines can be discontinuous and reduced to isolated dark spots (this variability has also been observed in *C. nicoletii*). Therefore, this common name should be reconsidered as it only leads to confusion between this and other similar species. In fact, there are many free-living silverfish identified as *C. lineatum* that probably correspond to other species, including undescribed ones (Supplementary material 4). The most updated description of *C. lineatum* has been presented in Molero Baltanás et al. (2012). Adults of this species have 3 pairs of abdominal styli, but young specimens (less than 8 mm) have developed only 1 or 2 pairs. The lanceolate shape of its femoral scales and the shape of its prosternum are also diagnostic characteristics of this species. This type of scale has also been observed recently in terminal filaments and styli (Supplementary Fig. S10 in Supplementary material 2). In addition, *C. lineatum* has 3 + 3 combs of macrochaetae on the seventh abdominal tergite, while other synanthropic *Ctenolepisma* have 2 + 2. The trichobothrial areas of the mesonotum are placed in the 2 last lateral combs, i.e., the anterior trichobothrial area is on the penultimate comb (N-1), while in the remaining synanthropic *Ctenolepisma* the anterior trichobothria are placed in the antepenultimate comb (N-2). Probably, with a future accurate taxonomic revision of the genus *Ctenolepisma* and a new generic subdivision established, *C. lineatum* (as type species of the genus) would be the only species remaining within this genus, and the others should be placed in different genera. Some species that have been considered as synonyms, such as *C. nicoletii* in the Western Mediterranean area (and probably in the Canary Islands), *C. brauni*  Wygodzinsky, 1941 in northern Africa, and *C. rubroviolaceum* (Schött, 1897) in SW North America, can be considered as valid species living in natural habitats in their respective distribution areas, where they can enter buildings accidentally. Detailed differences between these species and *C. lineatum* should be provided in the future; those with *C. nicoletii* were given by Molero Baltanás et al. (2012).

**Fig. 25.**
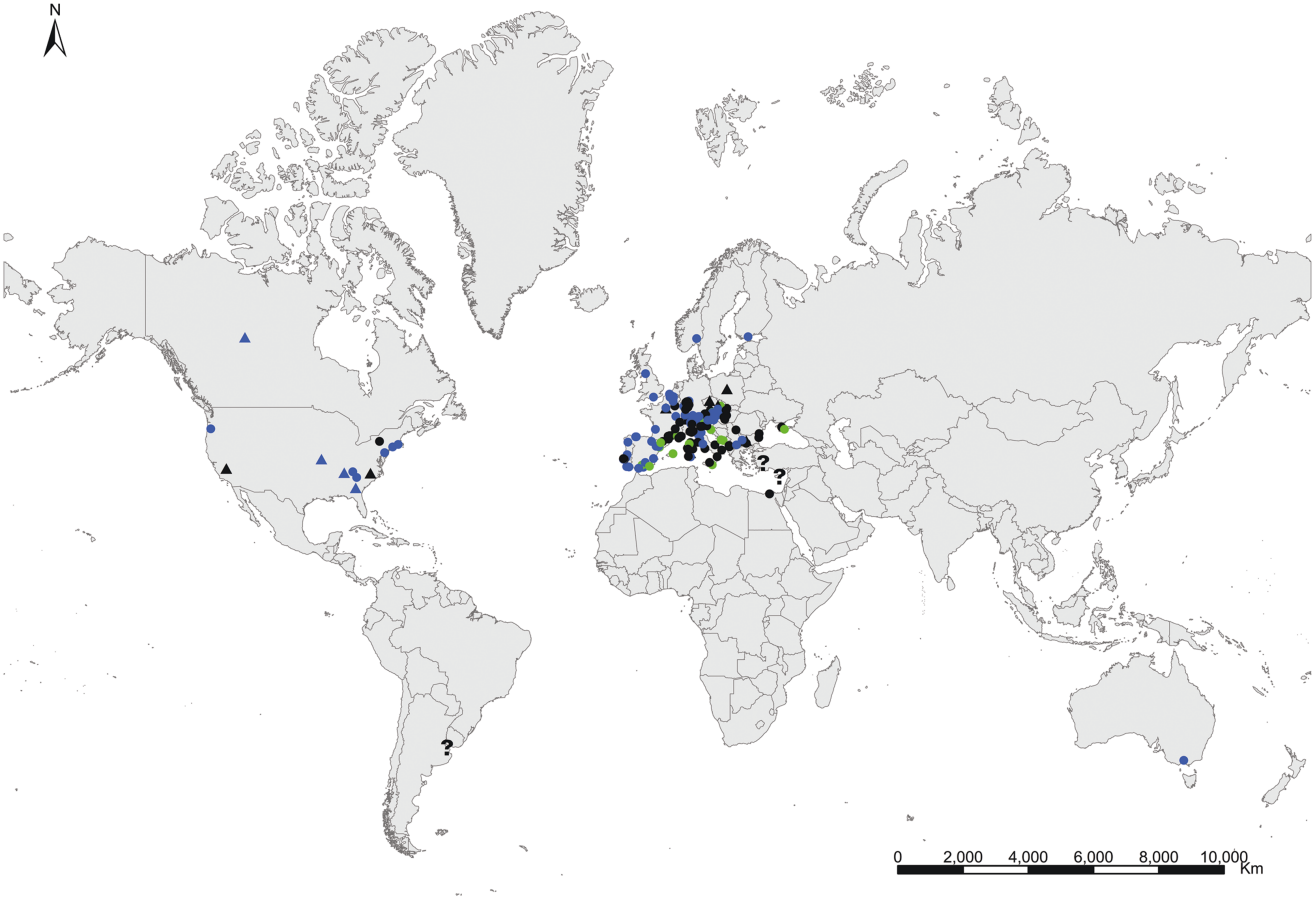
Distribution map of *Ctenolepisma lineatum*. Triangles (▲) indicate records with nonaccurate location, and circles (⚫) correspond to records with accurate location coordinates. Records are classified as without habitat information (black), synanthropic habitat (blue), and natural habitat (green). We have designated with a (**?**) those records where the specific identification of the silverfish cannot be assured based on the available information.

**Geographic distribution and origin** (Fig. 25). In southern and central Europe, *C. lineatum* is a facultative synanthropic species, with records both in natural habitats and in human buildings. Its extension in natural habitats of central Europe is not precise since some records do not specify the habitat where the insects were found, but it probably lives in warm locations where it is probably native. In the Iberian Peninsula, records as synanthropic are more frequent than those from natural habitats (generally found under rocks or bark). As some related species have been wrongly identified as *C. lineatum* (examples in Supplementary Material 4), it is difficult to establish the limits of its natural distribution. Previous reliable records of *C. lineatum* are detailed in Molero Baltanás et al. (2012), together with those new included in Supplementary Material 1. Outside of Europe, some records could correspond to *C. lineatum* or not, but it is very likely that this species is not native to other continents, so all the non-European records from natural habitats probably correspond to different species. As synanthropic, it has been recorded in Europe, North America, Argentina, and Australia. In several areas, especially in North America (where the knowledge of Lepismatidae is especially outdated), it has been cited with other names that are considered synonyms, such as *C. reticulatum* (Schött, 1897) or *C. quadriseriatum* (Packard, 1873). A complete list of synonyms can be consulted in Supplementary Material 1 or in Molero Baltanás et al. (2012). Here we provide the first records for Asturias (Spain) (Supplementary Material 1).

### *Ctenolepisma longicaudatum* Escherich, 1905

**Diagnosis.** Body length up to 16 mm. Macrochaetae plumose. The epidermic pigment is whitish to yellowish, slightly darker on the head and appendages. Dorsal scales are usually dark grayish or silvery gray but sometimes light grayish, light brown, or even transparent, with dense parallel ribs and a relatively homogeneous structure. Scales of coxae and femora are transparent and similar in shape to those of the body. Tibiae, tarsi, and remaining appendages lack scales. Cephalic chaetotaxy is typical of *Ctenolepisma* and is similar to that of *Ctenolepisma ciliatum*, as illustrated in Molero-Baltanás et al. (2010). Apical article of the labial palp usually has 5 sensory papillae arranged in a row, but some specimens can show a higher number (up to 12 papillae). Pronotum with setal collar. Thoracic nota with 9–12 pairs of lateral combs and with 1 + 1 posterior combs with 2–9 macrochaetae each. Anterior trichobothrial areas of the pronotum located next to the comb N-3 (the anterior to the antepenultimate lateral comb) or sometimes on comb N-4; anterior trichobothrial areas of the mesonotum located next to the comb N-2 (antepenultimate lateral). Prosternum with 2–4 pairs of lateral combs of macrochaetae on its subapical area. Mesosternum with 1–3 pairs of combs (usually 2 combs at each side, very close between them on the subapical area), and metasternum with 1 + 1 subapical combs of macrochaetae, arranged in a single row. The shape of these thoracic sternites is more similar to those of *C. armeniacum*  Molero-Baltanás, Gaju-Ricart, Bach de Roca, and Mendes, 2010, with the lateral margins of these sternites more convex, the anterior margin straighter, and the posterior margin less acute than those of *C. ciliatum*. Abdominal tergite I with 1 + 1 combs, II–VI with 3 + 3 combs, VII–VIII with 2 + 2 combs, urotergite IX bare; abdominal sternites lacking median combs and with 1 + 1 lateral combs on segments III–VIII. Urotergal combs have 6–13 macrochaetae and urosternal combs usually have 15–25 macrochaetae (usually, a lower number on urosternite VIII of some specimens). Urotergite X is trapezoidal, about 0.4 times longer than wide at its base, with a straight or slightly concave hind margin. Both sexes have 2 pairs of abdominal styli on segments VIII and IX (very young specimens can bear only 1 pair). Ovipositor long, with 40–45 divisions; its apex clearly surpasses the tip of the ninth styli, usually by more than twice the length of these styli.

**Morphological and taxonomic remarks.** One of the best-known, most studied, and widespread synanthropic silverfish species throughout the world. There are several available descriptions of this species (e.g., Aak et al. 2019, Thomsen et al. 2019), but most of them are incomplete or not clearly diagnostic. The photographs shown by Thomsen et al. (2019) and Bednár et al. (2023) illustrate good-quality specimens collected in the Faeroe islands and in Slovakia, respectively, and are useful for distinguishing this species from *Lepisma saccharinum.* However, some diagnostic characters required for comparison with other *Ctenolepisma* species are not mentioned. Some others are poorly illustrated or described and, at most, useful for distinguishing *C. longicaudatum* from the species of Lepismatidae previously known in the geographic range of those works, usually very concrete. In fact, the information provided could lead, in some cases, to a misidentification with some synanthropic species such as *C. calvum*, *C. rothschildi*, or *C. villosum*. For example, the description given by Aak et al. (2019) is useful for distinguishing it from *Lepisma saccharinum* but not from species of the genus *Ctenolepisma* with similar habitus. Extra details are given in Supplementary material 4.

The present diagnosis is based mainly on specimens collected in Spain, but material from other countries (Supplementary material 1) has been examined to check its variability. If we use most of the previous descriptions that use nonimportant taxonomic characters, overlooking others that should be used to distinguish *C. longicaudatum* from related species, some specimens of *C. calvum*, *C. villosum*, or some related free-living species such as *C. ciliatum*, *C. armeniacum* or *C. iranicum* Molero, Kahrarian, and Gaju, 2016 could be identified as *C. longicaudatum*. Although the free-living species of silverfish do not usually enter buildings, there is a possibility in their native distribution areas.

The epidermic pigment of *C. longicaudatum* is usually less intense than in these free-living *Ctenolepisma*, but there is a weak variability in this character, which is difficult to appreciate in dorsal view where the color of the scales hides the pigment. The most important difference between *C. longicaudatum* and the aforementioned species is the shape and chaetotaxy of the thoracic sternites. Illustrations of these sternites were given by Kaplin (1978), and new drawings are presented in this work (Supplementary Fig. S18 in Supplementary material 2). A dissection and microscopic examination of these ventral plates are advisable to compare its sternites with those presented in the descriptions of the free-living species (Molero-Baltanás et al. 2010, Kahrarian et al. 2016), their combs of macrochaetae are arranged in 2 rows in *C. armeniacum* and *C. iranicum*, their lateral margins are straighter and the apex more acute in *C. ciliatum*, etc. Other relevant characteristics to distinguish this species from related synanthropic silverfish with uniform silvery grayish color (due to dorsal cover of scales) are the absence of median urosternal combs (present in *C. villosum*) and bearing 2 pairs of styli in adults of both sexes (males of *C. villosum* and both sexes in *C. calvum* have only 1 pair). Coxae and femora of *C. longicaudatum* are covered ventrally by rounded scales similar to those of the body (usually a bit smaller in femora); femoral scales of *C. calvum* are different, although they are transparent and difficult to discern without microscopic examination. The anterior trichobothrial areas are associated usually to the anterior to antepenultimate lateral comb (N-3; sometimes to the N-4) on the pronotum, to the antepenultimate (N-2) on the mesonotum and to the penultimate lateral comb (N-1) on the metanotum. This arrangement is similar to other synanthropic Ctenolepismatinae, with the exception of *C. villosum* in the pronotum and of *C. lineatum* in the pronotum and mesonotum. In *Ctenolepisma longicaudatum* the usual number of labial papillae is 5, but some specimens from different countries show an unusually higher number (up to 12), and those with different numbers of papillae have been proposed to correspond to a different species. The species was described by Escherich (1905) on the basis of specimens from South Africa, but he did not mention the number of papillae in his description. Most authors did not give details on this character, although Womersley (1939) presented a drawing based on specimens from Australia with a higher number of papillae (about 8 in Fig. 7B of that paper) but did not mention this in the text. Slabaugh (1940), finding specimens in Urbana (IL, USA) with 5 sensory papillae, assumed that African specimens share with those from Australia this high number of papillae (9–12) and this is the typical form of *C. longicaudatum*, giving the name *Ctenolepisma urbana*  Slabaugh, 1940 to the form with 5 papillae. She also showed some additional differences between *C. urbana* and *C. longicaudatum* related to the arrangement of macrochaetae on frontal tufts or the shape of the hind margin of the last abdominal tergite, presenting figures of both forms but without clarifying the origin of the specimens of *C. longicaudatum* used for comparison. Apart from the number of papillae, the other characters have turned out to be variable in both forms. However, Paclt (1966), examining the types of *Ctenolepisma longicaudatum* in the Hamburg Museum, detected that South African specimens used for the original description of *C. longicaudatum* have 5 sensory papillae in their labial palps, so the typical form that should retain the specific name *longicaudatum* is that with this usual number of papillae. The question is complex since specimens with different numbers of papillae have been collected in the same country (for example, in Australia and South Africa), and no additional differences have been detected that distinguish the specimens with 9–12 papillae from those with the usual number. So, the correspondence of this difference with different taxa has not been proved, and *C. urbana* and other names have been proposed as synonyms of *C. longicaudatum*, considering that all forms suggested as different species actually belong to the same taxon (see synonyms in Supplementary Material 1). However, genetic differences have been detected in this work (Fig. 1) for specimens identified as *C. longicaudatum*. Those from northern Australia and Spain apparently match with the diagnosis presented in this work, but the specimen with GenBank accession number MT674899, collected in Tasmania, probably corresponds to a separate lineage congruent with establishing a different species (even closer to the free-living species *C. ciliatum* than to the typical *C. longicaudatum*), so this requires further investigation to identify the morphological differences that can be useful to distinguish both lineages and if they match with the 2 forms with different number of papillae or not. The specimen with GenBank accession number NC_073550, collected in China, is also clustered as a different taxon and perhaps it could be identified morphologically as a different species if experts were consulted; in our opinion, this third “lineage” of *C. longicaudatum* detected in the molecular tree could correspond to a species related to those recently described from India (Hazra et al. 2022b) that are very similar to *C. longicaudatum* but easy to distinguish if appropriate identification is carried out. So, specimens that do not correspond strictly to the typical *C. longicaudatum* are not marked in bold in Fig. 1 because they could probably be assigned to another species in the future.

**Fig. 26.**
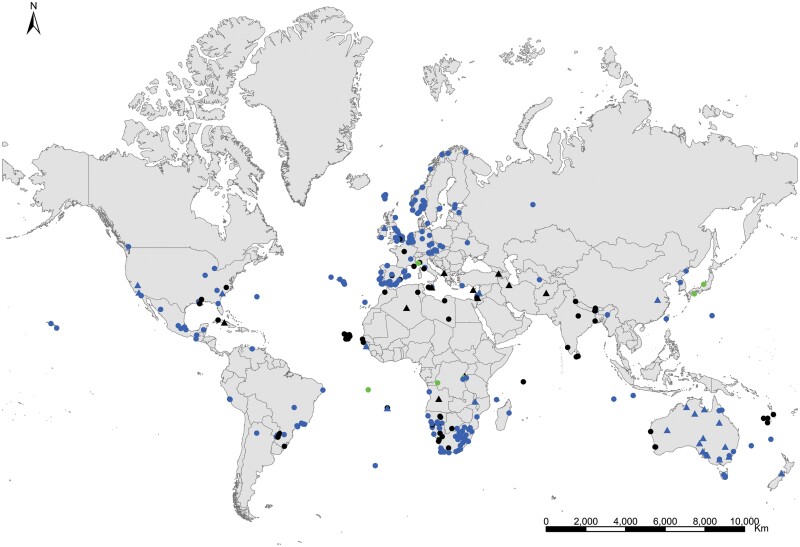
Distribution map of *Ctenolepisma longicaudatum.* Triangles (▲) indicate records with nonaccurate locations, and circles (⚫) correspond to records with accurate location coordinates. Records are classified as without habitat information (black), synanthropic habitat (blue), and natural habitat (green). We have designated with a (**?**) those records where the specific identification of the silverfish cannot be assured based on the available information.

**Geographic distribution and origin** (Fig. 26). As synanthropic, this is the most widespread species of silverfish, together with *Lepisma saccharinum*. Probably *C. longicaudatum* is even more common, and perhaps global warming is helping this species to be predominant because of its preference for higher temperatures. In the literature, we can find records of *C. longicaudatum* from many countries of all continents except Antarctica (Supplementary material 3). New records for Europe have become more frequent in the last decade, especially those of northern countries, but tracing the historical expansion of this species requires the use of different strategies and not only a web-based search. Kulma et al. (2021) collected a lot of records of the dubious reliability of this species in Europe but overlooked old scientific literature. For example, they marked with a question about the presence of this species in Malta when a record made by a Zygentoma specialist exists for this island (Mendes 1980). Furthermore, the first record of *C. longicaudatum* for Portugal dates back to 1945 and not to 1948 (Wygodzinsky 1945). Some other old but reliable records from the scientific literature on Zygentoma are not registered, resulting in the first records for some countries being earlier than those indicated in the aforementioned work (Supplementary Material 1). On the other hand, recent records that are only based on photographs and/or are published by nonspecialists cannot be considered reliable. These recent records should be checked because confusion with *C. calvum*, *C. rothschildi*, and *C. villosum* is possible (in some cases, even with *Lepisma saccharinum*; see cases in Supplementary Material 4). Misidentifications are not only recent but probably frequent before the taxonomic revision of Escherich (1905) that gave the first accurate reference for identification by nonexpert entomologists. So, we think that *C. longicaudatum* would have been found in Europe more than a century ago. For example, the species *Ctenolepisma tavaresi*  Navás, 1906, described from Lisbon, can be considered as *species inquirenda* because of the loss of types and the poor original description, but it is very likely that it could correspond to *C. longicaudatum* (Mendes 1978).

In spite of errors that can distort its precise current and past distribution, it is clear that *C. longicaudatum* is almost cosmopolitan, and it has expanded to the north in recent decades. Here, we have provided the first records for Ukraine and Asturias (Spain) (Supplementary material 1). The biology under laboratory conditions of this species is one of the best known of Zygentoma (Aak et al. 2019), but its geographic origin is not clear. Although some records of *C. longicaudatum* correspond to natural habitats in several Atlantic islands such as Saint Helena, Ascension, and the Cape Verde archipelago, we think that it has been introduced in these areas and become feral because of the absence of competition with other Lepismatidae. In our opinion, the original area of this species could be placed in SW Asia, in a steppe zone between Turkey and India, because species with more similar morphological characteristics (*C. armeniacum*, *C. iranicum*, *C. ciliatum*, even *C. mauritanicum* (Lucas, 1846)) occur in this area. This or some close geographic regions could have been the dispersal center of this species.

### *Ctenolepisma rothschildi* (Silvestri, 1907)

**Diagnosis.** Body length up to 8.5 mm. Macrochaetae plumose. The epidermic pigment is light yellowish to brownish, more intense on the head, apex of styli, and sometimes on the legs. Dorsal scales are variable in color, usually dark grayish or brownish to blackish, and show different patterns (uniform, with dark spots, etc.). Dorsal scales are very diverse in size, shape, and structure; some large scales with about 12–14 spaced ribs are remarkable on the anterolateral part of thoracic nota, combined with smaller scales with 20–30 more dense rays. Ventral scales are more uniform. Coxae have broad rounded scales similar to those of the body, and the inner side of the femora is covered with smaller and narrower scales that can be rounded to subtriangular truncate. Scales on tibiae have not been detected. Tarsi and remaining appendages apparently lack scales. Antennae and terminal filaments are usually not longer than body length. Cephalic chaetotaxy typical of *Ctenolepisma*, with 1 + 1 frontal tufts of macrochaetae forming subtriangular areas on the frontal margin, clypeus, and labrum with 1 + 1 tufts each, those of the labrum smaller. Apical article of the labial palp with 5 sensory papillae in a single row. Pronotum with setal collar. Thoracic nota with 6–9 pairs of lateral combs and with 1 + 1 posterior combs of 3 macrochaetae each. Anterior trichobothrial areas of the pronotum located next to the comb N-2 or N-3 (the antepenultimate lateral comb or the anterior to antepenultimate); anterior trichobothrial areas of the mesonotum located next to the comb N-2 (antepenultimate lateral). Each thoracic sternite with 1 + 1 subapical combs of 3–7 macrochaetae. Abdominal tergite I with 1 + 1 combs, II–V with 3 + 3 combs, VI–VIII with 2 + 2 combs, urotergite IX bare; abdominal sternites lacking median combs and with 1 + 1 lateral combs on segments III–VIII. Abdominal combs have few macrochaetae, and the infralateral ones usually have 3–6 macrochaetae, the sublateral and submedian combs with 2–3 macrochaetae each, and the urosternal combs with 3–9 macrochaetae. Urotergite X trapezoidal, short (ratio length-width of the trapezoid about 0.3), with straight or slightly concave hind margin. Two pairs of abdominal styli on segments VIII and IX, although the styli on segment VIII is only developed in adult specimens. The inner process of the coxite IX is about 3.3–3.5 times longer than the outer process; in males, this process is about 1.1 times as long as broad at its base 1.8 times in females. Ovipositor not very long, with about 30–35 divisions; its apex surpassing the tip of styli by 1–2 times their length (or exceeding the apex of the inner process of the ninth coxite by more than 4 times the length of the process).

**Morphological remarks.** The most detailed available description of this species was given by Smith (2012) on the basis of specimens collected in Australia. Other descriptions have become obsolete, including those of specimens attributed to different species, but are now considered synonyms (*C. diversisquamis* Silvestri, 1908 and *C. reducta*). As suggested by Smith (op. cit.), the combination of the arrangement of urotergal combs, the occurrence of heterogeneous scales dorsally (Fig. 13C), especially those described as pauciradiate (with few longitudinal ribs or rays), the number of abdominal styli and the shape of the urotergite X, can be enough to identify this species. Some details of differences with related species are presented in Supplementary material 1. In some cases, misidentifications are possible with *C. calvum* since both species show pauci-radiate dorsal scales and are relatively small compared with related synanthropic species. Nevertheless, the dorsal scales of *C. calvum* are more uniform (Fig. 13B), and adults of *C. rothschildi* bear 2 pairs of styli (only 1 pair in *C. calvum*) and 5 labial papillae (3 in *C. calvum*). The longer ovipositor of *C. rothschildi* and the higher number of macrochaetae in their combs are also characteristics that help with this distinction. Given its small size compared with other synanthropic *Ctenolepisma*, it can be identified as a juvenile of other species if the specimens are only observed by eye or even by stereomicroscope if the key characters are not correctly detected (for example, the scales can be lost in poorly preserved specimens). The color pattern of *C. rothschildi* seems to be variable since the original description (Silvestri 1907) states that these insects are brownish; in the original description of *C. diversisquamis*, the scale cover is described as reddish-black or black-green (Silvestri 1907), in the description of Puerto Rican specimens given by Folsom (1923) it was described as “silvery white, mottled with dark brown scales dorsally,” and Australian specimens are described as having dark dorsal scales (Smith 2012). We have seen uniform grayish-brown specimens from Cape Verde and Iran, so the color pattern is not a diagnostic character for this species. This variability could have led to the establishment of different taxa in the past, but the synonymy with *C. diversisquamis* (originally described from the Cape Verde Islands) was confirmed by Irish (1995), and the synonymy with *C. reducta* (described from Puerto Rico) was established by Wygodzinsky (1972). Other names, such as *C. brachyura* Silvestri, 1918 and *C. incita* Silvestri, 1918, both assigned to specimens collected in Kenya, are proposed as synonyms of *C. rothschildi* (Paclt 1967; Irish 1995). Without discarding the idea that more than 1 species is involved, our description is mainly based on specimens from Boavista (Cape Verde Islands), which is one of the type localities referred to in the original description of this species, and no significant differences have been observed with specimens from other geographic areas (Iran, Australia) that we have examined. Another silverfish with a similar aspect is *C. nigrum* (Oudemans 1890), mainly distributed in SE Asia, but this species has 3 + 3 combs of macrochaetae in the urotergite VI (where *C. rothschildi* has 2 + 2 combs). It is likely that *C. nigrum* could enter houses in SE Asia, where confusion between both species is possible (see more details of this species in Supplementary Appendix 1).

**Fig. 27.**
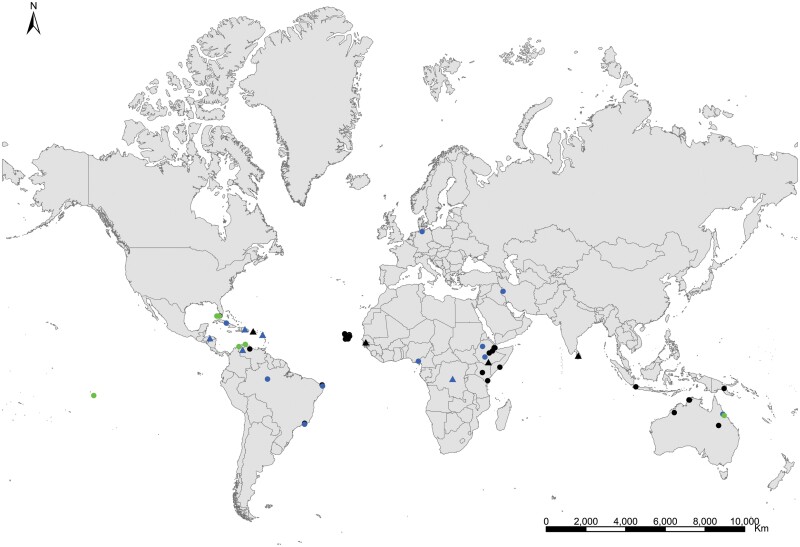
Distribution map of *Ctenolepisma rothschildi.* Triangles (▲) indicate records with nonaccurate locations, and circles (⚫) correspond to records with accurate location coordinates. Records are classified as without habitat information (black), synanthropic habitat (blue), and natural habitat (green). We have designated with a those records where the specific identification of the silverfish cannot be assured based on the available information.

**Geographic distribution and origin** (Fig. 27). This species has been reported mainly from tropical countries in America, Asia, and Africa (Supplementary Material 3), and few records exist out of these areas. Apart from the aforementioned record from Australia (Smith 2012), it has been recorded in the United States (Wygodzinsky 1972). In Europe, it has been registered in Hamburg (Germany) by Paclt (1966). So, this species should be added to the key of synanthropic silverfish from Central Europe given by Querner et al. (2022). Nevertheless, the aforementioned record could correspond to *C. calvum* and not to *C. rothschildi*, but this has not been checked. Wygodzinsky (1972) indicated that *Ctenolepisma rothschildi* had been dispersed by man but cannot be clearly considered as strictly synanthropic; in his key of Caribbean Lepismatidae it is included as *C. diversisquamis*. Probably in the Caribbean area, this species is introduced and can be found in natural habitats of small islands where it has naturalized because of the absence of competition with other Lepismatidae, but in large islands where native *Ctenolepisma* are present, this species is not found in these habitats (as suggested by the observations of the first author in Puerto Rico) and in those areas it can be considered as strictly synanthropic. *Ctenolepisma diversisquamis* and *C. reducta* are names given to this species in several records in the 20th century. Most of its records correspond to domestic or peridomestic habitats, although in some cases, the circumstances of capture are not detailed in the literature. The publications where it is expressly mentioned that this species is collected under stones or in natural habitats can give a clue for its original habitat; unfortunately, they are scarce, and perhaps some of them correspond to insects that have naturalized after their introduction, such as those from Cape Verde Islands. Nevertheless, its original area could be very likely found in tropical Africa since this region is the only one where other species showing 2 types of scales (big pauciradiate and smaller with dense rays) can be found in natural habitats. For example, *C. brachyura* has been found in Mount Kenya (if this species is actually a synonym of *C. rothschildi*, this represents a locality within the original area of this species). These species are probably phylogenetically related, and sub-Saharan Africa could have been the area where they evolved.

### *Ctenolepisma (Sceletolepisma) villosum* (Fabricius, 1775) = *Ctenolepisma (Sceletolepisma) targionii* (Grassi and Rovelli, 1889), new synonym

Figs. 28–31

**Fig. 28.**
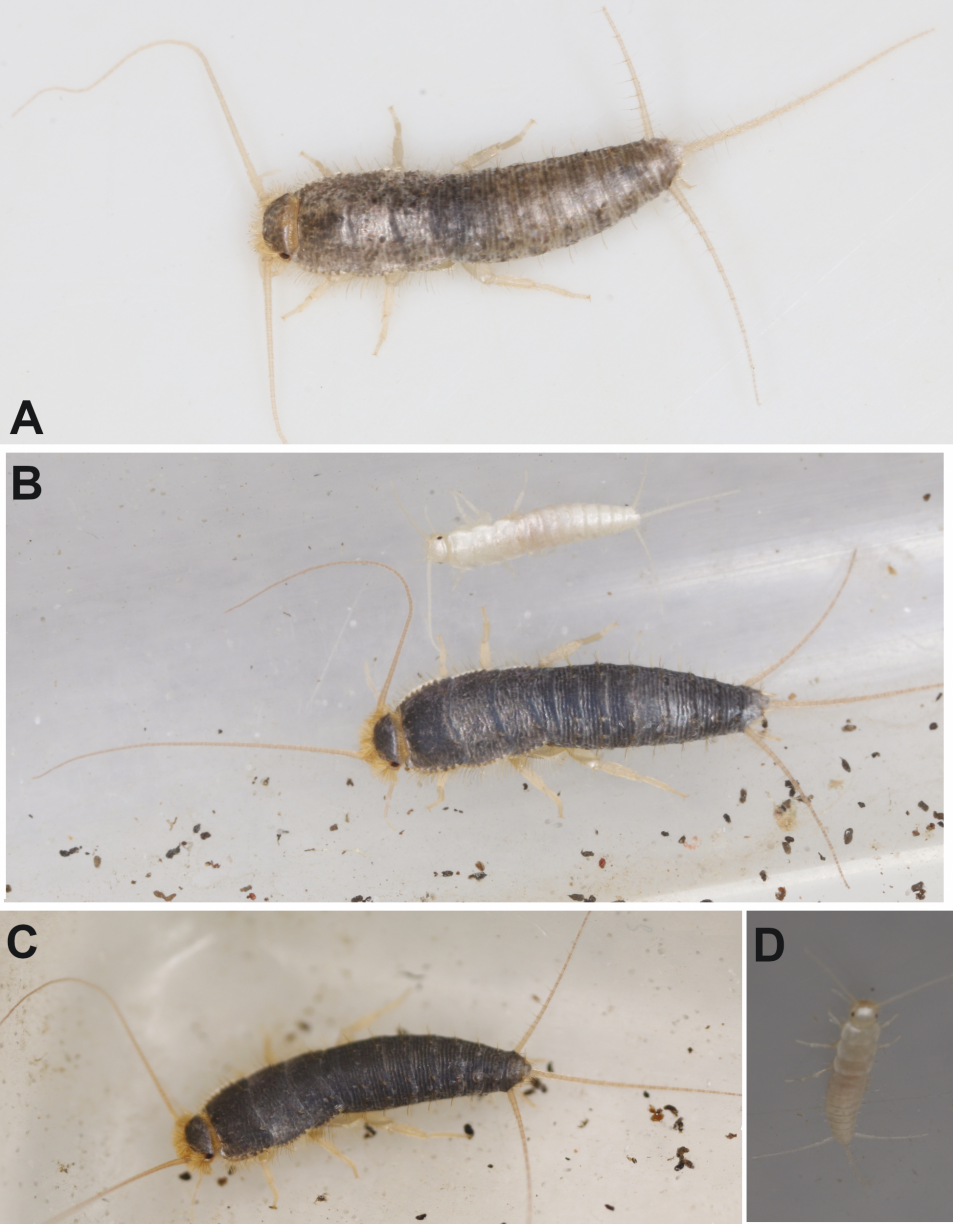
*Ctenolepisma* (*Sceletolepisma*) *villosum*, the habitus of living specimens found in Spain. A), B), and C) Adult specimens with darker B, C) or lighter A) grayish scales. B) and D) A young specimen with whitish or transparent scales (not all young specimens are whitish). The body length of adults is 8–9 mm, and the length of the young specimen is about 5 mm.

**Fig. 29.**
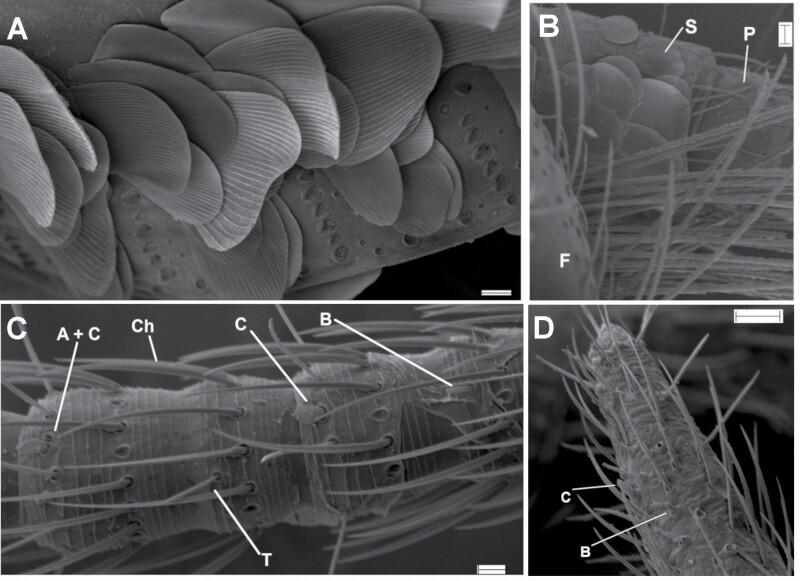
*Ctenolepisma* (*Sceletolepisma*) *villosum* redescription based on Spanish specimens. SEM photographs. A) Dorsal scales of the pronotum surrounding some lateral combs. B) Basal part of the antenna and frons (F), showing the scapus covered by scales (S) and the pedicel devoid of scales (P). Macrochaetae of the frontal tuft are clearly feathered. C) Some flagellomeres of the antenna have different types of sensilla. D) The apical part of the maxillary palp shows basiconic sensilla type B and C. B: basiconic sensillum type B. C: basiconic type C. A + C: pair of basiconic sensilla (diad), one type A very close to one type C. Ch: chaetic sensillum, typically mechanoreceptor. T: Trichoid sensillum. Scales: 20 µm, except for C) 10 µm.

**Fig. 30.**
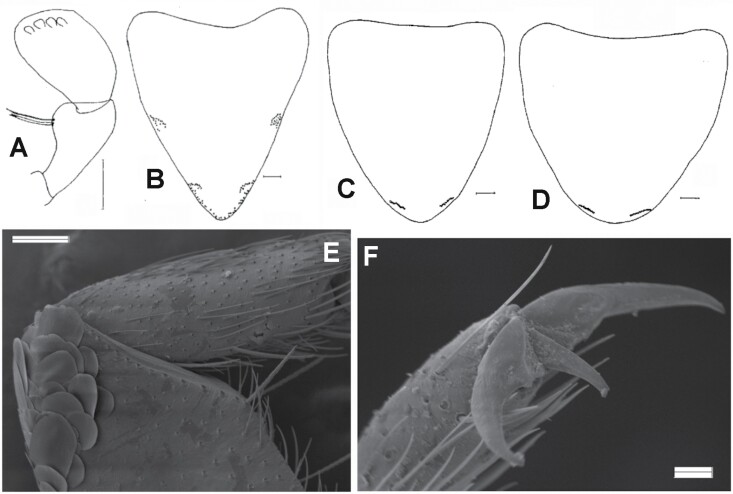
*Ctenolepisma* (*Sceletolepisma*) *villosum* redescription based on Spanish specimens. A) Labial palp. B) Prosternum. C) Mesosternum. D) Metasternum. E) Outer (dorsal) side of a femur and a tibia. F) Pretarsus. Scales: 0.1 mm, except for E) 20 µm.

**Fig. 31.**
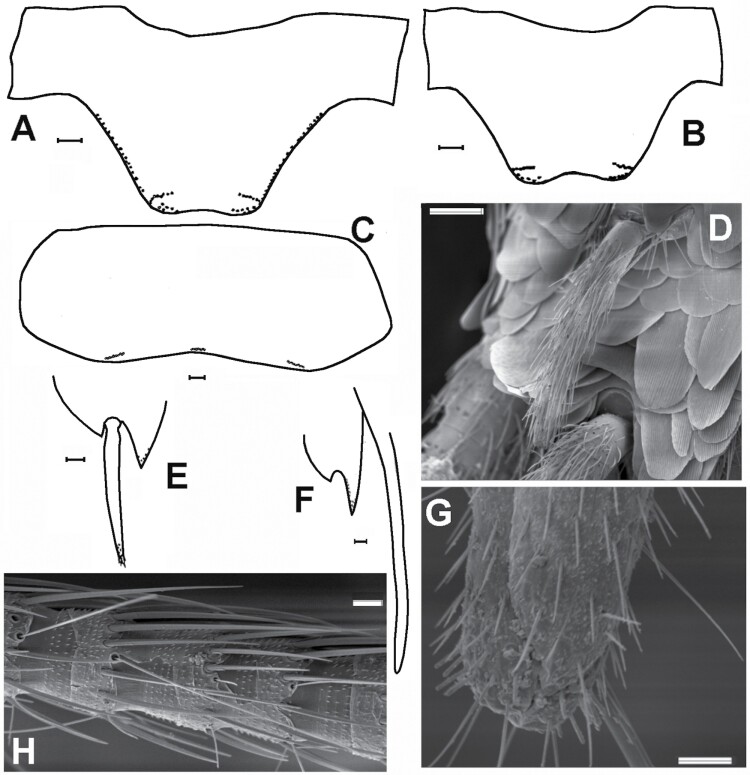
*Ctenolepisma* (*Sceletolepisma*) *villosum* redescription based on Spanish specimens. A and B) Variability in the shape (especially in the hind margin) of the urotergite X of 2 specimens. C) Urosternite V, showing median comb and lateral combs. D) Stylus VIII and coxite IX are covered with scales, with the base of stylus IX. E) Coxite IX and stylus IX of the male. F) Coxite IX and ovipositor of an adult female. G) Apex of the ovipositor. H) Some basal divisions of the cercus. Scales: 0.1 mm, except for G) and H): 20 µm.

**Diagnosis.** Macrochaetae plumose. The epidermic pigment is whitish to yellowish, slightly darker on the head and appendages. Dorsal scales are usually dark grayish (almost blackish) or silvery gray, but sometimes light grayish, light brown, or even transparent, with dense parallel ribs but relatively heterogeneous structures. Scales of coxae and femora are transparent and similar in shape to those of the body. Tibiae, tarsi, and remaining appendages lack scales. Cephalic chaetotaxy with 1 + 1 clypeal tufts of macrochaetae, 1 + 1 small tufts on the labrum, and a median gap on frontal margin between the large frontal tufts. Apical article of the labial palp with 3–5 sensory papillae arranged in a row (specimens with 4 or 5 are more frequent than those with only 3). Pronotum with setal collar. Thoracic nota with 8–13 pairs of lateral combs and with 1 + 1 posterior combs with 2–7 macrochaetae each (some last lateral combs of the metanotum can be reduced to 1 macrochaeta). Anterior trichobothrial areas of the pronotum and mesonotum are usually associated with the comb N-2 (antepenultimate lateral comb), sometimes with comb N-3 on the pronotum. Prosternum with 2 + 2 combs of macrochaetae close to the lateral margins of its apical area. Mesosternum and metasternum with only 1 pair of combs. These combs usually have 9–14 macrochaetae each. Abdominal tergite I with 1 + 1 combs, II–V with 3 + 3 combs, VI–VIII with 2 + 2 combs, urotergite IX bare. Urotergal combs have about 4–6 macrochaetae, although infralateral combs can have a higher number. Abdominal sternites II–VI with 1 median comb of 6–12 macrochaetae. Abdominal sternites III–VIII with 1 + 1 lateral combs of 7–12 macrochaetae. Urotergite X trapezoidal, about 0.32–0.45 times longer than wide at its base, with a straight or slightly concave hind margin. Adult females have 2 pairs of abdominal styli on segments VIII and IX; males only bear 1 pair. Ovipositor long, with more than 40 divisions, its apex clearly surpasses the tip of styli by 2–3 times its length or even more.

**Examined material.** Specimens from previously published studies from Spain, Italy, and Iran have been studied and reexamined for the redescription. Some additional samples from Spain as new records have also been studied (Supplementary Material 1). We have provided the first records for Albacete and Ciudad Real (Spain) (Supplementary Material 1).

**Redescription.** Medium-size silverfish with body fusiform, thorax a little wider than the abdomen, which tapers posteriorly. Scale patterns are usually uniform dark grayish dorsally, but the tonality of dorsal scales is variable, ranging from almost transparent or hyaline (especially in juveniles) to dark brownish, almost black (see Fig. 28).

**Body size.** Body length up to 10 mm, thorax length up to 3.1 mm, thorax width up to 2.2 mm. Maximum preserved antennal length 6 mm, probably slightly longer when intact. Maximum preserved length of a terminal filament 7 mm.

**Pigmentation and scales.** Body pigment is nearly absent, whitish to light yellowish. Macrochaetae hyaline is yellowish and feathered, with abundant pectinations. Scales with numerous parallel or subparallel ribs that do not surpass the margin of the scales (Fig. 29A), those of dorsal side ovoid, rounded, orbicular, with the apical margin rounded or straight, usually grayish, those of ventral side hyaline or light yellowish. Some dorsal scales show deep indentations associated with bare points of the dorsal surface, described by Irish (1996) as unscaled spots. Ventral scales are rounded or rectangular. Scales cover all of the body but are absent from appendages, with the exception of scape (Fig. 29B), coxae, and femora. Femoral scales are rounded, similar to those of coxae (Fig. 8B).

**Head.** Chaetotaxy is similar to that of other *Ctenolepisma* species, with 1 + 1 frontal tufts that have about 100 macrochaetae or more arranged in irregular rows (the row closer to the median gap has 11–14 macrochaetae in adult specimens). The median gap between these frontal brushes is about as wide as the width of the brushes. There are 1 + 1 groups of periocular macrochaetae surrounding the inner margin of the eyes consisting of about 25 macrochaetae arranged in 2–3 rows and 1 + 1 elongated frontolateral tufts of about 20–30 macrochaetae between the frontal tufts and the periocular ones, separated from the frontal tufts by a narrow gap and almost connected to the periocular groups by a row of the frontolateral tufts. An isolated group of 6–9 macrochaetae is placed above this frontolateral tuft. Clypeus with 1 + 1 tufts of about 50 macrochaetae; there are some thin and long acute setae dispersed on the median area. The 1 + 1 tufts inserted laterally on the labrum are small, consisting of a group 6–10 macrochaetae. These numbers are lower in young specimens.

**Antenna.** Scapus longer than wide, with scales similar in shape to those of the body and some setae apically. The Pedicel is shorter, without scales, less than half the length of the scapus, and slightly narrower, with a subapical ring of numerous setae and some others dispersed in its basal part. The flagellum has several types of sensilla: trichobothria, chaetic (arranged in rings or rosettes), trichodea, coeloconic, and basiconic (types A, B, and C) have been detected using SEM (Fig. 29C).

**Mouthparts.** Mandibles with molar and incisor areas; about a dozen smooth bifid setae are inserted close to the molar area on the inner side, and more than 100 macrochaetae form a big, elongated tuft on the outer side. Maxillary palp long, apical article about 6–7 times longer than wide and as long as the penultimate article, with some basiconic sensilla of types B and C (Fig. 29D); the antepenultimate a little shorter. Apical article of labial palp club-shaped, with 3–5 papillae arranged in a single row; the most frequent number seems to be 4 (Fig. 30A), but 3 have been seen in some Japanese specimens and 5 in Egyptian and some Spanish representatives. This apical article is as long as wide and about as long as the penultimate article of the palp.

**Thorax.** The setal collar of the pronotum occupies a little more than half of the width of the anterior margin of the pronotum and consists of several subparallel oblique rows of 4–6 macrochaetae. The setae of the anterolateral rows of this margin of the pronotum are smooth, without pectinations or very few. Pronotum with 8–9 pairs of lateral combs on each side, mesonotum with 11–13 lateral combs, and metanotum with 9–11, composed of 2–7, 2–5, and 1–4 macrochaetae each. Hind margin of nota with 1 + 1 posterior combs, with 3–7 macrochaetae each. Trichobothrial areas of the pronotum are separated by 2 lateral combs (i.e., trichobothria are located on the N and N-3 combs), although in some specimens, the anterior trichobothrial areas are located on the antepenultimate comb (N-2) and the trichobothrial areas are separated by only 1 comb. In the mesonotum, the trichobothrial areas are separated by 1 lateral comb, i.e., they are located on the last and antepenultimate combs (N and N-2); those of the metanotum are associated with the last 2 pairs of lateral combs (N and N-1). Lateral macrochaetae of the nota are long, from 1/2 to 1/3 of the length of each notum; the trichobothria, rather long, reach or even exceed 1/4 of the length of the corresponding notum. Prosternum, as in Fig. 30B, is about 1–1.1 times as long as wide at its base, subtriangular and acute apically, shorter than the other 2 thoracic sternites, with 2 + 2 combs of 8–10 macrochaetae that are very separated from each other (the anterior comb is close to the middle of the lateral side of the sternite). Mesosternum and metasterntum as in Fig. 30C and D, both with only 1 + 1 subapical combs of 12–14 macrochaetae. The mesosternum is longer than wide at its base (ratio length/width about 1.1), but the metasternum is 0.9–1 times as long as wide, i.e., a little wider than long. The distance between the apical combs of the metasternum ranges from 0.45 to 2 times the width of a comb.

**Legs.** Quite long; ratio length/width of protibiae up to 3.1, the same ratio of metatibiae 4.0–4.5. Protibiae with 2 dorsal and 3–5 ventral macrochaetae; metatibiae with 3 dorsal and 4–6 ventral macrochaetae. In all legs, the coxa, the inner side of the femur (Fig. 8B), and the apical part of the outer side of the femur (Fig. 30E) have rounded orbicular scales similar to those covering the body. Tibiae and tarsi only with setae. Pretarsus with smooth claws and a small empodium (Fig. 30F).

**Abdomen.** Urotergite I with 1 + 1 combs of macrochaetae in infralateral position. Urotergites II–V with 3 + 3 combs of macrochaetae. Urotergites VI–VIII with 2 + 2 combs of macrochaetae (in infralateral and lateral position). Urotergite X bare. Combs B and C (those that can usually be observable dorsally in lateral and submedian position) with 4–6 macrochaetae, exceptionally 7. Infralateral combs (combs A) with 5–9 macrochaetae, usually more than 6. Urotergite X trapezoidal with rounded posterior corners; its ratio length/width is 0.32–0.45, and its hind margin can be almost straight or clearly concave; the 1 + 1 combs of this urotergite have 7–9 macrochaetae each (Fig. 31A and B). Urosternites III–VIII with 1 + 1 lateral combs consisting of 7–12 macrochaetae. Urosternites II–VI with a median comb of 6–12 macrochaetae (Fig. 31C). Males with only 1 pair of styli associated with the ninth abdominal segment. Females always have 2 pairs of abdominal styli (Fig. 31D). Coxite IX with its inner process about 1.1–1.3 longer than wide at its base in males (Fig. 31E) and 1.5–2 times longer than wide in females (Fig. 31F). The inner process is about 4–5 times longer than the outer process in both sexes (close to 4 in males). These metrics are lower in juveniles. In both sexes, the styli IX is 2–2.5 times longer than the inner process of the coxite IX (in males, the difference of length is usually higher). In adult females, the ovipositor has 33–42 divisions and can surpass the tip of the inner process of the coxite by more than 3 times its length (Fig. 31F; not so long in young females). The apex of gonapophysis as in Fig. 31G. Penis as usual in Lepismatidae. Males without parameres, as in other Ctenolepismatinae.

**Terminal filaments.** Cerci as in Fig. 31H, with strong macrochaetae, chaetic sensilla, trichoid sensilla, and trichobothria.

### Taxonomic Discussion

The genus *Ctenolepisma* was divided into 2 subgenera by Wygodzinsky (1955): *Ctenolepisma* s. str. and *Sceletolepisma*. Irish (1987), in his revision of South African *Ctenolepisma*, maintained this division using a different character than Wygodzinsky to separate both subgenera: *Sceletolepisma* sensu Irish bears median urosternal combs of macrochaetae, while *Ctenolepisma* s. str. lacks median combs on urosternites. The species *Lepisma villosa* Fabricius, 1775, described from China, and *Lepisma targionii* Grassi and Rovelli, 1889, described from Italy, were included in *Ctenolepisma* by Escherich (1905) but considered different species. As they have median urosternal bristle combs (concretely in urosternites II–VI), they were placed in the subgenus *Sceletolepisma* sensu Irish. This subgenus is going to be raised to a generic level in a future publication.

The descriptions of *Ctenolepisma targionii* based on specimens from Egypt (Stach 1935) and of *C. villosum* (as *Ctenolepisma villosa*) based on specimens from Japan (Yosii 1939), which can be considered as the most updated and detailed descriptions of both taxa, have been compared with each other and with specimens collected in Spain and Italy and previously identified as *C. targionii* (Supplementary Table 1 in Supplementary Material 4). All descriptions are essentially identical. The only detected differences between both descriptions that can be considered relevant concern the number of labial papillae (5 in Egyptian specimens, 4–5 in Spanish insects, and 3–4 in Japanese specimens) and the number of divisions of the ovipositor, and both of them can be attributed to intraspecific variability. The number of labial papillae seems to be variable, both in western populations considered as *C. targionii* and in eastern ones identified as “*C. villosa*.” We consider the possibility that Yosii (1939) overlooked 1 or 2 labial papillae because sometimes when dirt particles are present in the apical area of the last article, they are difficult to see even in microscopic slides. If this is what happened in his study of Japanese silverfish, both Asian and European populations could have the same range of variability (4–5 papillae). Variability in the number of these papillae has also been reported for other synanthropic species like *C. longicaudatum*. Regarding the divisions of the ovipositor, Stach (1935) indicated that Egyptian *C. targionii* has 33–35 divisions, and Yosii (1939) indicated that the ovipositor of Japanese “*C. villosa*” has about 40 divisions. The available specimens from Spain and Italy have a variable number of divisions, from 35 to 42, overlapping the variability of the Egyptian and Japanese records. Other characteristics, such as the number of macrochaetae of abdominal combs, are apparently different in Egyptian *C. targionii* (11–15) and Japanese “*C. villosa*” (6–7), overlap when including the higher number of specimens previously identified as *C. targionii* (more than 20) from Spain and Italy (7–12 macrochaetae). It is likely that Yosii studied subadult specimens. In Supplementary material 4 (Supplementary Table S1), we have included a complete comparison where the differences observed between the description of *C. targionii* by Stach and that of “*C. villosa*” by Yosii can be considered negligible, and coincidences (sometimes, surprisingly, even in the writing style) are evident. So, these characteristics are not clear enough to separate the forms from Europe, previously identified as *C. targionii*, and those from Asia identified as *C. villosum* as different species. Mitochondrial DNA sequences support this statement: the sequence provided by Chen et al. (2019) on Chinese specimens of *C. villosum* is identical to that obtained on *C. targionii* collected in Spain (Fig. 1), compatible with a hypothesis of conspecificity. So, morphological and molecular evidence is presented to state that the species *Ctenolepisma targionii* and *Ctenolepisma villosum* are synonyms. As *Lepisma villosa* was described earlier, the name “*villosum*” is the one that will be retained.

The covering of scales on appendages, the position of trichobothrial areas, as well as the shape and chaetotaxy thoracic sternites of this species, are described here for the first time. Compared with *C. longicaudatum*, this latter character is clearly different (compare Fig. 24B–D and Supplementary Fig. S18 in Supplementary material 2), but the other 2 are very similar. *Ctenolepisma villosum* has an almost identical appearance to *C. longicaudatum*; although adults are usually slightly smaller than those of *C. longicaudatum,* both species can be correctly distinguished only with a microscopic study. The most important and remarkable diagnostic character for separating *C. villosum* from *C. longicaudatum* is the presence of median bristle-combs in some urosternites, typical for the subgenus *Sceletolepisma* sensu Irish. Other characteristics are, for example, the occurrence of only 1 pair of abdominal styli in males of *C. villosum*, the lower number of macrochaetae of the abdominal combs, or the aforementioned different shape and chaetotaxy of thoracic sternites. Some misidentification errors between *C. longicaudatum* and *C. villosum* are possibly undetected since their distinctive characteristics cannot be clearly detected by eye or usual photographs. Generally, almost all the unstudied records (mostly from citizen sciences platforms) of the specimens are attributed to *C. longicaudatum*, which could be an underestimating of the actual distribution of *C. villosum* in the entire world. In some specimens, scales are whitish, whitish gray, or almost transparent; this is frequent in young specimens (Fig. 28B and D), and a whitish appearance has also been observed in specimens that have lost partially or totally their dorsal scales, so confusion with *C. calvum* is also possible if an accurate study is not made. Probably, some identifications of *C. calvum* based on photographs in southern Europe actually correspond to *C. villosum*. Some glands associated with the apical area of the ultimate article of the lapial palp can be seen by transparency inside the article, occupying the majority of the volume of this article, which is different from what has been observed for *C. longicaudatum,* where these glands are restricted to the apical part of the article (Molero-Baltanás et al. 2015). We do not know if this difference is constant for both species or whether it could be a diagnostic character distinguishing *Sceletolepisma* from *Ctenolepisma* s. str. This requires further study.

**Fig. 32.**
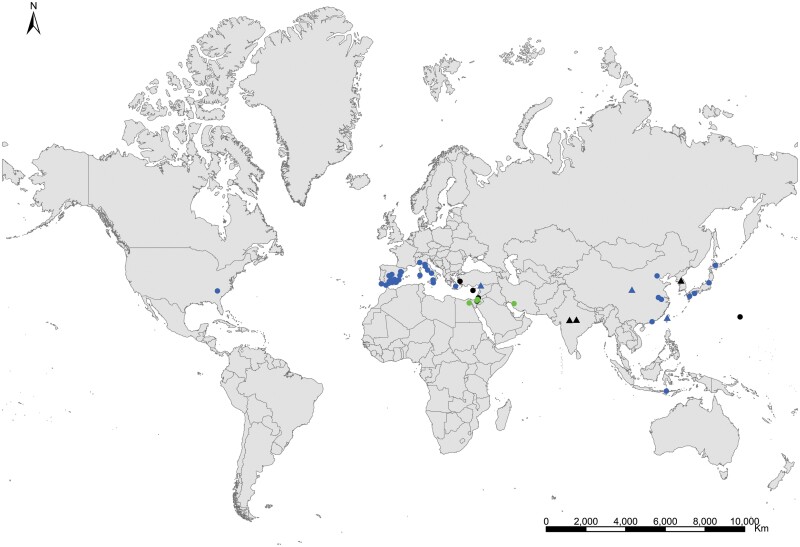
Distribution map of *Ctenolepisma* (*Sceletolepisma*) *villosum*. Triangles (▲) indicate records with nonaccurate locations, and circles (⚫) correspond to records with accurate location coordinates. Records are classified as without habitat information (black), synanthropic habitat (blue), and natural habitat (green). We have designated with a those records where the specific identification of the silverfish cannot be assured based on the available information.

**Geographic distribution and origin** (Fig. 32). This species has been recorded as *Lepisma targionii* or *Ctenolepisma targionii*, in several countries of the Mediterranean Region and in the Near East (Supplementary material 3). Moreover, one record of this species from America was given by Wygodzinsky (1972). As *Lepisma villosa* or *Ctenolepisma villosa* or *C. villosum*, it has been recorded in China, Japan, and some other areas of SE Asia (Supplementary material 3). Probably some records in other temperate or subtropical geographic areas of domestic *Ctenolepisma* with dorsal uniform color could correspond to misidentified *C. villosum*. Most of these records do not mention the habitat where the insects were found, but several records from the SE Mediterranean basin and SW Asia (Egypt, Israel, and Iran) suggest that this species is autochthonous in arid regions of NE Africa and SW Asia.

### *Thermobia aegyptiaca* (Lucas, 1840)

**Diagnosis.** Body length up to 13 mm. Body fusiform, with plumose macrochaetae; antennae and terminal filaments longer than the body when intact. Epidermic pigment yellowish white, with dark areas on some annuli of antennae and terminal filaments, palps, head, apical margins of the femur, part of the tibia and the first tarsomere, styli, and ovipositor. Appendages are devoid of scales, except for scapus, coxae, and femora, absent from the remaining parts of appendages, those of coxae and femora similar in shape to the scales covering the body. All ventral scales are hyaline or whitish. Maxillary palp with 5 articles. The apical article of the labial palp is slightly longer than wide, with 5 papillae arranged in a single row. Thoracic nota with 8–12 lateral combs of macrochaetae, and 1 + 1 posterolateral combs of 6–8 macrochaetae. Anterior trichobothrial areas of the pronotum are located on the lateral comb N-4 or N-5; posterior trichobothrial areas of the pronotum are on the antepenultimate lateral comb N-2. Trichobothrial areas of the mesonotum are located on the antepenultimate lateral comb (anterior) and on the last lateral comb (posterior). Trichobothrial areas of the metanotum on the 2 last lateral combs. Prosternum with 5–8 + 5–8 combs of macrochaetae, relatively wide in its median part, mesosternum with 1–2 + 1–2 combs, and metasternum with 1 + 1 combs. Urotergite I with 1 + 1 infralateral combs of macrochaetae, urotergites II–VII with 2 + 2 combs. Urotergite VIII has 2 + 2 combs, but in some populations, the submedian comb is reduced to 1–2 small macrochaetae and is frequently overlooked. Urotergite IX devoid of setae. Urotergite X is short, subtriangular, and posteriorly rounded, with 1 + 1 combs. Urosternites I–II without setae, urosternites III–VI with a median comb, and urosternites IV–VIII with 1 + 1 lateral combs. Adults bearing 3 pairs of abdominal styli. The ovipositor is slender, of primary type, surpassing the tip of the styli IX by 1–1.5 times their length.

**Morphological remarks.** The most updated description of this species was given by Irish (1988) in his revision of the genus *Thermobia*. Some characteristics, such as the scale distribution on appendages and the arrangement of trichobothrial areas on thoracic nota, have been incorporated into the present diagnosis. The scale pattern of this species has not been described. Most specialists have studied specimens preserved in alcohol, where the original color of the scales is lost. There are several photographs in some web platforms attributed to this species that suggest that the pattern is similar to that of *T. domestica*, but identifications at specific levels of these insects cannot be confirmed. It is very likely that the scale pattern of this species is variable, from uniformly dark brown to bicolor or tricolor, with patches or transverse rows of dark scales alternating with lighter scales (yellowish or brownish), but it has not been included in the diagnosis since it requires further study. As indicated by Irish (1988), the third pair of styli appears when the body length of the insect reaches 7 mm. Moreover, the genus *Thermobia* requires revision. Palearctic *T. aegyptiaca* and *T. domestica* are very similar in the new characters used for the taxonomy of these insects, but we have examined specimens of *T. vallaris* from South Africa and observed that they are different, at least in the covering of scales of legs (unpublished data). Perhaps these differences could also be observed in South African “*T. aegyptiaca*,” correlating with the variability of the chaetotaxy of the urotergite VIII. If insects identified previously as *T. aegyptiaca* correspond to at least 2 different species, as Irish (2018) suggests, the species that will retain the name *T. aegyptiaca* is the Palearctic one (described from Egypt), and this is probably the form that has been spread to North America as synanthropic. We think that the specimen included in our tree as *Thermobia* sp. belongs to this species, considering its geographic origin, but no additional fresh specimens in better conditions were available for DNA sequencing, so the position of this species in the tree remains unresolved, and the present diagnosis is the reference for correct identification of specimens that will be sequenced in the future.

**Fig. 33.**
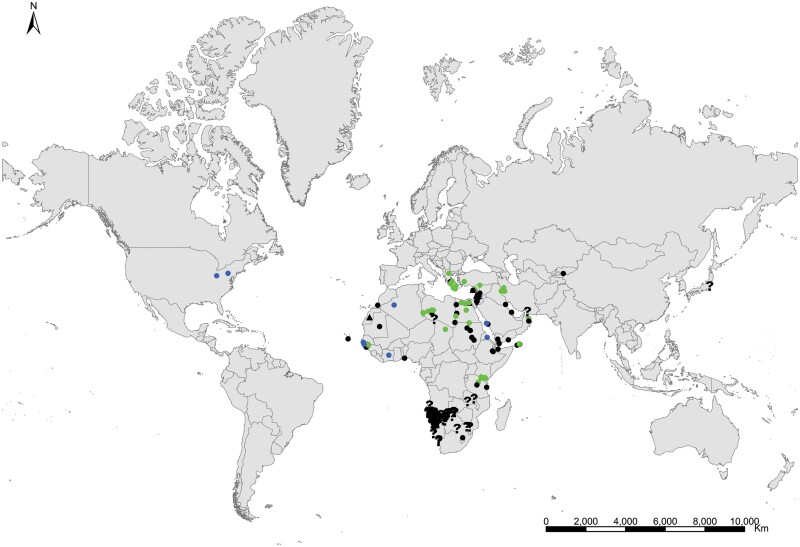
Distribution map of *Thermobia aegyptiaca*. Triangles (▲) indicate records with nonaccurate locations, and circles (⚫) correspond to records with accurate location coordinates. Records are classified as without habitat information (black), synanthropic habitat (blue), and natural habitat (green). We have designated with a (**?**) those records where the specific identification of the silverfish cannot be assured based on the available information.

**Geographic distribution and origin** (Fig. 33). This species occurs in natural habitats of almost all of Africa and the Near East (Southwest Asia) and is probably native to all this area. It reaches southeastern Europe in Greece. The available records were compiled by Irish (1988) and most of them are indicated in the Supplementary material 3. In North America, it occurs only as synanthropic. Perhaps it occurs in other countries or continents where it could have been misidentified with *T. domestica*.

### *Thermobia domestica* (Packard, 1873)

**Diagnosis.** Body length up to 11 mm, males usually slightly smaller than females. Antennae longer than body and terminal filaments as long as body when intact. Epidermic pigment absent to yellowish white. Dorsal scales are yellowish, whitish, and/or light brownish, with darker patches that tend to form transverse bands. Appendages are devoid of scales, except for scapus, coxae, and femora, absent from the remaining parts of appendages, those of coxae and femora similar in shape to the scales covering the body. All ventral scales are hyaline or whitish. Maxillary palp has 6 articles, resulting from a secondary subdivision of the apical article; there is a suture that divides the fifth article into 2 segments, which is very difficult to discern in young insects. An apical article of the labial palp with 5 papillae is arranged in a single row. Thoracic nota with 7–12 + 7–12 lateral combs of macrochaetae (usually a higher number of combs on the pronotum than in the meso- and metanotum) and with 1 + 1 posterolateral combs. Anterior trichobothrial areas of the pronotum usually associated to the comb N-4 or N-5, sometimes to the N-6, located on an anterior position, ahead of the middle length of the sclerite; posterior trichobothrial areas in the N-2 comb. Anterior trichobothrial areas of the mesonotum and of the metanotum as in *Thermobia aegyptiaca*. Prosternum with 5–8 + 5–8 combs of macrochaetae, narrowing in its median part and slightly truncated in its apex. Meso- and metasternum with 2 + 2 subapical combs. Urotergite I with 1 + 1 infralateral combs of macrochaetae, urotergites II–VIII with 2 + 2 combs. Urotergite IX is devoid of setae. Urotergite X is short, subtriangular, and posteriorly rounded, with 1 + 1 combs. Urosternites I-II without setae, urosternites III–VI with a median comb, and urosternites IV–VIII with 1 + 1 lateral combs. Adults of both sexes bear 3 pairs of abdominal styli. Ovipositor is slender, of primary type, surpassing the tip of the styli IX by 1–2 times their length.

**Morphological remarks.** The most updated description of this species, commonly known in English as “firebrat,” is available in Irish (1988), although characters such as the scales of appendages and arrangement of trichobothrial areas of the nota (illustrated in Supplementary Material 2, Supplementary Figs. S8, S9, and S16) have been added to the present diagnosis. The arrangement of trichobothrial areas of the nota is apparently exclusive to *Thermobia* species (at least of both Palearctic species studied in this work). *T. domestica* is very similar to *T. aegyptiaca* (Lucas 1840). The main difference is the division of the last article of the maxillary palp (Irish 1988), which in *T. domestica* is a 6-segmented maxillary palp (Fig. 16B), while all remaining silverfish, including *T. aegyptiaca* have 5-segmented palps (Fig. 16A). Some other differences noted by Irish (1988) relate to the chaetotaxy and shape of thoracic sternites, the chaetotaxy of the eighth urotergite, pigmentation pattern of appendages, etc., but there is variability in these characters that have not been sufficiently assessed. For example, the prosternum drawn by Irish (1988) shows a truncate apex, but the prosternum of Palearctic specimens examined in this work has a more acute apex (Supplmentary Fig. S19B in Supplementary Material 2), similar to that of *T. aegyptiaca*. Irish commented that the metasternum of *T. aegyptiaca* has 1 + 1 combs while *T. domestica* has 2 + 2, but Palearctic *T. aegyptiaca* frequently have 2 + 2 combs (sometimes the combs of one or both sides can be confluent), so there is no significant difference in this character between this species and *T. domestica* (Supplementary Fig. S19 in Supplementary Material 2) In the same way, both species are similar in the chaetotaxy of the urotergite VIII, contrary to observations of Irish (1988) based on South African specimens attributed to “*T. aegyptiaca*” where this author observed a reduction of the submedian bristle-combs; this slightly different *Thermobia* probably belongs to a different undescribed free-living species, as the same author suggested (Irish 2018). Compared with most *Ctenolepisma* species, the firebrat has a different scale pattern: *T. domestica* usually has a set of alternating black and yellowish bands, illustrated by Wygodzinsky (1972); black bands are usually transverse, not longitudinal as in *C. lineatum* and other species with similar pattern. However, these patterns are variable, and worn specimens totally lose them. This variability has probably led to the misidentification of some *Ctenolepisma* species with *Thermobia domestica*, as suggested by the molecular tree presented in this work that includes the sequences of some specimens identified as *Thermobia* inside the branch of the tree that corresponds to correctly identified *Ctenolepisma* species (Fig. 1). The most definitive diagnostic characteristics are related to chaetotaxy. Species of the genus *Thermobia* only show 2 + 2 urotergal bristle combs, while those belonging to *Ctenolepisma* have at least 4 urotergites (II–V or more) with 3 + 3 combs of macrochaetae. *Ctenolepisma* species lack median bristle combs on urosternites (except those of the subgenus *Sceletolepisma*, including *C. villosum*, that are going to be separated soon in an independent genus), but *Thermobia* species have median combs on urosternites III–VI. The types and patterns of antennal sensilla were described by Adel (1984). *Thermobia domestica* has been used as a representative model of Zygentoma in a lot of anatomical and biological studies, but perhaps other species are studied under this name.

**Fig. 34.**
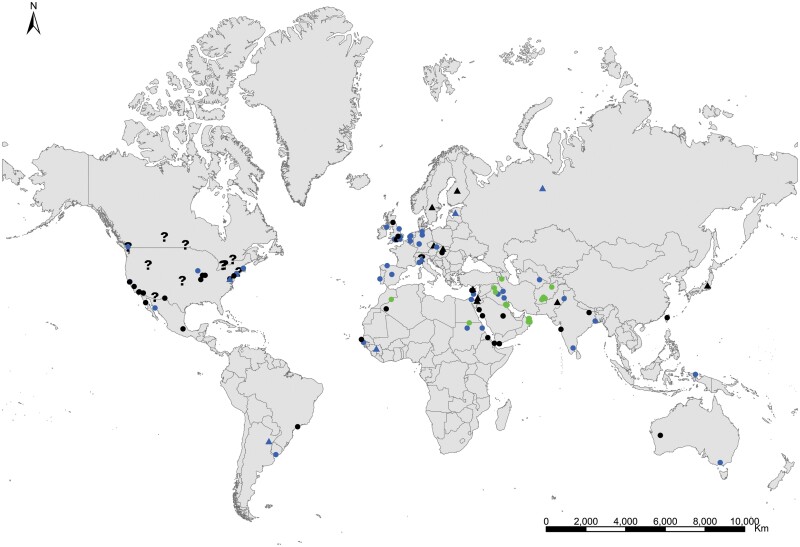
Distribution map of *Thermobia domestica*. Triangles (▲) indicate records with nonaccurate locations, and circles (⚫) correspond to records with accurate location coordinates. Records are classified as without habitat information (black), synanthropic habitat (blue), and natural habitat (green). We have designated with a (**?**) those records where the specific identification of the silverfish cannot be assured based on the available information.

**Geographic distribution and origin** (Fig. 34). We have provided new records for Algeria, Uruguay, and Spain (Asturias). This species can be considered almost cosmopolitan since it has been recorded in many countries on all continents. The species is reared to feed exotic reptiles in pet shops, and it has been inadvertently transported by human trade. However, some identifications are based only on photographs, and the scale color pattern can be shared at least with *T. aegyptiaca* and probably with other poorly studied nondomestic silverfish. Thus, identification has not been confirmed with the examination of the reliable taxonomic characters of these insects detailed in the present diagnosis. In the Iberian Peninsula, this species was previously recorded in 1 bakery in Portugal by Mendes (1980) and recently in Madrid (Spain) (Bernal et al. 2022). The specimens studied here were found on pallets deposited next to a bazaar in an industrial park. This species has been recorded in natural habitats in Northern Africa and Southwestern Asia (from Morocco to Afghanistan, see Supplementary material 3), suggesting that this area of the south Palearctic is probably the geographic origin of the domestic populations. There are also some records of natural habitats in North America. Although Wygodzinsky (1972) stated that this species has been introduced in this continent and the populations found in natural environments are secondary (feral), Irish (1988) thinks that *T. domestica* could also be native to North America and the origin of the genus *Thermobia* has occurred prior to the breakup of Pangea, and he uses the example of *Ctenolepisma* as having a similar disjunct distribution. As stated above in the section of *T. aegyptiaca*, the genus *Thermobia* requires revision, together with other genera of Ctenolepismatinae. Some unpublished studies made by us on North American native *Ctenolepisma* suggest that they are very different to those from the Old World and, similarly, native “*Thermobia*” could correspond to different autochthonous taxa more closely related to *Leucolepisma*. This hypothesis requires confirmation with a detailed revision of North American Ctenolepismatinae.

### Other Synanthropic Records

In the Iberian Peninsula and the Balearic Islands, 2 other silverfish species have been found inside human dwellings: *Ctenolepisma nicoletii* and *C. ciliatum.* These synanthropic records should be considered to be accidental since the number of records in human buildings is low compared to the number of records in natural habitats. The records of the synanthropic cases of these 2 species are also provided in Supplementary Material 1. Some species of *Ctenolepisma* or *Thermobia* that are native to other geographic areas could be occasionally found in human habitats in those areas (e.g., *C. abyssinicum* Mendes, 1982 or *C. plusiochaetum* Silvestri, 1922). Their differences with those that are synanthropic are indicated in Supplementary Material 1. Some other species of Zygentoma have been recorded in peridomestic habitats, but their classification as synanthropic is doubtful, considering that they have been found mainly in other habitats or there are few records. This is the case for species of Lepismatidae belonging to the rare genus *Namunukulina*  Wygodzinsky, 1957 (*N. funambuli*  Wygodzinsky, 1957 and *N. congolensis* Mendes, 1982). *N. funambuli*, described from squirrel nests in South Africa, was further found in human rooms in Peru (Wygodzinsky 1959). *N. congolensis*, described from swallow nests, was later found in the gardens of a hotel in Gambia (Irish 1986) and could be considered peridomestic. These species belong to the family Silvestrellatinae, which bear feathered macrochaetae and lack paramera like *Ctenolepisma* or *Thermobia* but lack setal collar, and their dorsal setae do not form combs as in *Lepisma*. This combination of characters is enough to distinguish them from other synanthropic genera, and as they are not treated here as indisputable synanthropic species, they are not included in the identification key. Finally, Wygodzinsky (1991) includes *Nicoletia phytophila* Gervais, 1844, a species of the family Nicoletiidae, in his list and identification key of American synanthropic species, but in urban environments, this insect is only found in tropical gardens in greenhouses, associated with soil that has been transported from tropical regions. Considering this accidental occurrence in human habitats, this species is not considered in the identification key. Anyway, *Nicoletia* can be distinguished from synanthropic silverfish by the lack of eyes and scales, which are present in all Lepismatidae (these are the characters used in the key of Wygodzinsky for identifying *Nicoletia phytophila*).

## Discussion

Identification of pests and potential introduced and invasive species should be made accurate, especially in groups of invertebrates that are poorly known because of the scarcity of specialists or the unavailability of appropriate identification material. Accurate identifications are also important before conducting expensive molecular studies. Expert assistance is required; however, adequate resources are not available for reliable identification. This work represents an important consolidation in the knowledge of the most worldwide distributed synanthropic Lepismatidae species. We hope that the provided identification key based on truly reliable characters, the updated morphological diagnosis of each species, at least one COI gene sequence of a specimen correctly identified, and the extensive additional Supplementary material help future researchers, nonspecialists, and citizen scientists to correctly identify Zygentoma species. Furthermore, although the morphological descriptions are sufficient for the identification of silverfish species, the combination with molecular studies has been proven to be useful for detecting several previous misidentifications.

We are sure that some of the published sequences of *Thermobia domestica* are based on incorrectly identified specimens: those that cluster with our sample of *Ctenolepisma lineatum* in our tree, with GenBank accessions AF370848, MN448219, MG375320, MN556951, and those with BOLD Process IDs MOBIL1017-15 and GMBUA1578-14. We have found the origin of some of these sequences. For example, in the study of Espinasa et al. (2007), 1 specimen labeled as *Thermobia* cf. *domestica* (GenBank accession DQ280136) but the other, labeled as *Thermobia domestica* (AF370848) clusters with our sample of a correctly identified *Ctenolepisma lineatum*. This suggests that in some countries where both species occur, misidentifications have been frequent. This is likely because the scale pattern of both species is variable: that of *T. domestica* does not always form transverse patches, and that of *C. lineatum* does not always form longitudinal stripes, or perhaps people identifying these insects assumed a scientific name without examining appropriate characters. A lot of records of *T. domestica* in North America probably correspond to *C. lineatum*, so further research is needed to clarify the distribution of these species in the mentioned areas. Moreover, the complete mitochondrial genome sequence published by Bai et al. (2020) for *Lepisma saccharinum* very likely represents an unidentified silverfish of the genus *Ctenolepisma* and does not correspond to *Lepisma*, because the COI sequence provided in the aforementioned work fits in a part of the tree very far from correctly identified *Lepisma saccharinum*. Online databases usually apply high standards for sequence quality, but there appear to be limited, if any, standards for ensuring the quality of species identifications attached to sequence data. Therefore, any work that has included these incorrectly identified sequences for phylogenetic analysis would have been making errors. The new genetic data for certain species, such as *Acrotelsa collaris* and *Ctenolepisma rothschildi* ensures that at least there is well-identified baseline data for all common synanthropic species on which we can continue studying the relationships between silverfish or using this as a tool to identify doubtful specimens. Furthermore, this data is useful for unraveling cryptic diversity. For example, *C. longicaudatum* is one of the most widely spread synanthropic silverfish, and our results suggest the possibility that this taxon could correspond to 2 or more closely related and similar species. To clarify this situation, morphological differences and geographic distribution of the different lineages should be assessed in the future. The research on the diversity of synanthropic silverfish does not finish with this work; it is likely that some undescribed species of *Ctenolepisma* and *Thermobia* await accurate description, and molecular tools will help to detect these taxa. We cannot state that the *Ctenolepisma* sp. sequenced by Bai et al. (2020) as *L. saccharinum* corresponds to a synanthropic species since the habitat where the specimens used for this study was not mentioned; the same happens with other singletons that are not clustered with any previously described synanthropic species.

Considering the taxonomic and phylogenetic implications of the relationships provided in this work, we can compare the morphology of the clusters that arise from the molecular study and obtain some conclusions. For example, morphological differences between *C. lineatum* and *C. nicoletii* that were considered sufficient to separate both species (Molero Baltanás et al. 2012) have genetic support. Differences in COI genes validate characteristics such as the shape and distribution of the scales, the shape and chaetotaxy of the thoracic sternites, and the number of pairs of styles, which are useful for the distinction between closely related species. Moreover, all taxa of *Ctenolepisma* with trapezoidal urotergite X are close in the tree, although it is possible that there are several clades, so this suggests that they could have a common ancestor, which would have to be confirmed with the incorporation of a greater number of free-living species and with other sources of DNA data such as nuclear genes. However, the taxa of the same genus with a short and convex subtriangular urotergite X remain in a very separate clade, with the genus *Thermobia* standing between them. This suggests that forms like *C. lineatum* represent a very distinct lineage and that the shape of the urotergite X is a good character for separating genera. The urotergal chaetotaxy is not so relevant for the supraspecific classification of *Ctenolepisma* species, in spite of the proposal of Kaplin (1993). For example, species with 2 + 2 pairs of combs on urotergite V, such as *C. villosum*, *C. calvum*, and *C. rothschildi* that Kaplin grouped (forming genera or subgenera without biogeographic sense) occupy branches of our tree in which other species with 3 + 3 combs on urotergite VI are interposed, suggesting that divisions based on both types of abdominal chaetotaxy do not have much phylogenetic value. In any case, to demonstrate which characters are more plesiomorphic within Ctenolepismatinae, it would be necessary to have a greater number of representatives in this subfamily. Our phylogenetic tree places *Thermobia* species in an intermediate position between *Ctenolepisma lineatum* and related species (sharing with *Thermobia* the subtriangular shape of the urotergite X) and the group that includes *Ctenolepisma* species with trapezoidal urotergite X (sharing with *Thermobia* the arrangement of trichobothria and the pattern of scales on appendages, among other characters). This indicates that *Ctenolepisma* is not a monophyletic group and that the suprageneric division of Ctenolepismatinae, including *Ctenolepisma* + *Thermobia* requires further revision.

Identifying silverfish at the species level can be difficult work. Therefore, doubtful identifications and evident misidentifications have been detected regularly in the literature and in citizen science platforms where identifications are based only on dorsal view photographs. Including these records in databases is not advisable if their low reliability is not indicated as they can distort the monitoring of the expansion of several synanthropic species. Reviewing scientific literature (especially works written by specialists) is, for the moment, the best strategy to trace the actual and past distribution of these insects. It is interesting to note all the records summarized here and the probable misidentifications detected suggest that the expansion of most synanthropic silverfish species in most countries is probably older than currently believed. For example, *C. longicaudatum* was recorded (and published) for the first time in Europe in Portugal by Wygodzinsky (1945), which does not agree with the information provided by Kulma et al. (2021). But there are a lot of previous records of *Lepisma saccharinum* or other Lepismatidae from this country that could correspond to misidentifications of *Ctenolepisma longicaudatum* or any other species of Ctenolepismatinae. For example, *Ctenolepisma tavaresi*  Navás 1906 was described on the basis of specimens collected in a school in Lisbon (Navás 1906). Mendes (1978) could not find the types of this species but, considering its incomplete description and which synanthropic species were found in Portugal, stated that this is not a valid species and probably corresponds to *Ctenolepisma longicaudatum*. If so, the species was established in Portugal at least at the beginning of the 20th century. If other identification mistakes, such as those made by Bai et al. (2020) or those presented in Supplementary Material 4, were made in Europe before 1940; we can interpret that this species was established in Europe earlier than was registered in literature records. So, it is impossible to accurately record their expansion in most areas in previous centuries if records confirmed by Zygentoma specialists are not available.

## Conclusions

Nine species of synanthropic silverfish are almost globally distributed, although their distribution is difficult to determine reliably because of frequent misidentifications by non-experts and the lack of good references. Some published DNA sequences of these species clearly are based on misidentifications, and their comparison with sequences of reliably identified specimens has allowed us to reidentify them correctly. In the future, molecular studies of additional specimens and species from throughout the world should be assisted by expert identifications or, at least, be based on accurate morphological examination using keys and resources such as those provided in this work. Although molecular data is a really useful tool, it needs to be combined with a morphological review to continue spreading the knowledge of the different species and to monitor the distribution of the synanthropic silverfish in future decades.

## Supplementary Material

ieae045_suppl_Supplementary_Materials_1

ieae045_suppl_Supplementary_Materials_2

ieae045_suppl_Supplementary_Materials_3

ieae045_suppl_Supplementary_Materials_4

## Notes

This work is dedicated to the memory of Luis Mendes, who guided us in the study of Zygentoma and has recently passed away.
