# Supplementary material for: Worldwide revision of synanthropic silverfish (Insecta: Zygentoma: Lepismatidae) combining morphological and molecular data
Source: J Insect Sci. 2024 May 4;24(3):1. doi: 10.1093/jisesa/ieae045 (PMC11069193; doi:10.1093/jisesa/ieae045)
Supplement: ieae045_suppl_Supplementary_Materials_1 [file ieae045_suppl_supplementary_materials_1.pdf]

Supplementary Material 1. Examined specimens and related species[1].

This document includes a list of all synanthropic silverfish examined for this work with more detailed information and a list of synonyms for each considered species (the symbol † is used for a relevant misidentification included in the list). Studied specimens are separated in unpublished material (New records) and previously published material (Additional material examined). First records for a country or a province are marked with an asterisk (*). When a specimen has been used for genetic analysis the accession number is added. A commented list of non-synanthropic species (but occasionally found in houses) similar or related to synanthropic species of this manuscript and studied for morphological comparison is included at the end this supplementary material. Abbreviations as follows. For facilitating navigation across the document, enable the navigation panel in view.

AC. Authors personal collections.
AMS. Australian Museum, Sydney.
BOS. Biology of Organism and Systems collection of University of Oviedo, Spain.
MNCN. Museo Nacional de Ciencias Naturales (National Museum of Natural Sciences), Madrid, Spain.
MUHNAC. Museu Nacional de História Natural e da Ciéncia, University of Lisbon, Portugal.
MNKB. Museum of Natürkunde of Berlin, Germany.
UCO. University of Córdoba, Department of Zoology collection, Spain. Most specimens of this collection will be deposited definitively in MNCN and other institutions.

[1]Supplementary document to the paper «**Worldwide revision of synanthropic silverfish (Insecta: Zygentoma, Lepismatidae) combining morphological and molecular data» (2024)**. By Rafael Molero-Baltanás, Andrew Mitchell, Miquel Gaju-Ricart & Jairo Robla in *Journal of Insect Science.*

Order **Zygentoma** Börner, 1904

Family **Lepismatidae** Latreille, 1802

Subfamily **Acrotelsatinae** Mendes, 1991

Genus ***Acrotelsa*** Escherich, 1905

Acrotelsa collaris (Fabricius, 1793**)**

Synonyms. *Lepisma collaris* Fabricius, 1793; *Lepisma niveofasciata* Templeton, 1843; *Lepisma mucronata* Packard, 1873; *Lepisma cincta* Oudemans, 1890;

New records. CAPE VERDE: Boavista, Tapada Pecho Santis: 1♀ + 2 juveniles (N. López *leg.*), 26-IX-2005 (UCO, Ref. Z2207); Boavista, Riveira Calhau: 2♂ + 2♀ + 4 juveniles (N. López *leg.*), 30-IX-2005 to 03-X-2005 (UCO, Refs. Z2210, Z2212, Z2219 and Z2223); Pico Farcado: 1♀ + 1 juvenile (N. López *leg.*), 29-IX-2005 (UCO, Ref. Z2211); Boavista, Lajada de Curral Vaca: 2 juveniles (N. López *leg.*), 03-X-2005 (UCO, Ref. Z2213); Boavista, Rocha Estancia: 2♀ + 2 juveniles (N. López *leg.*), 21-IX-2005 (UCO, Ref. Z2216 and Z2217); Boavista, Ladeira Estancia: 1♂ (N. López *leg.*), 02-X-2005 (UCO, Ref. Z2218); Boavista, Santo Antonio: 1♂ + 2♀ (N. López *leg.*), 27-IX-2005 (UCO, Ref. Z2221). Boavista, Rochinha: 1♀ + 2 juveniles (N. López *leg.*), 21-IX-2005 (UCO, Ref. Z2222); Sal island, Murdeira: 1♀ (N. López *leg.*), 26-VI-2007 (UCO, Ref. Z2850). **INDIA**: Varanasi: 1 *ex.* (Sandreep Kumar *leg.*), 14-V-2017 (AMS, Ref. K541563). Specimen used for genetic analysis (OR732107).

Additional examined material. Specimens studied in Kahrarian et al. (2014) from Iran have been re-examined.

Subfamily **Lepismatinae** Mendes, 1991

Genus ***Lepisma*** Linnaeus, 1758

Lepisma saccharinum Linnaeus, 1758

Synonyms. *Forbicina plana* Geoffrey, 1762; *Lepisma vulgare* Scopoli, 1763; *Lepisma semicylindrica* Degeer, 1782; *Forbicina semicylindrica* Geoffroy, 1762; *Forbicina saccharina* de Geer, 1785; *Machilis cylindrica* Lacordaire & Boisduval, 1835; *Lepisma quercetorum* Wygodzinsky, 1945; † *Lepisma myrmecobium* non Silvestri *sensu* Paclt, 1966: 149; Mendes, 1988: 26 [confirmed misidentification after examining specimen studied by Paclt in Zoologisches Institut und Zoologisches Museum, Hamburg].

New records. AUSTRALIA: Tasmania, Mt. Stuart, 1 Raymont: 1♂ + 1♀ (Steven Bunton *leg.*), 23-IV-2020, in a private garage (AMS, K541610 & K541611). Specimens used for genetic analysis (OR732104 & OR732108). **PORTUGAL**: Algarve, Lagos: 1♂ (R. Molero *leg.*), 04-IV-2007, in a hotel (UCO, Ref. Z2664). **SPAIN: Asturias**: Mieres, Turón, Yana'l Monte: 1♀ (J. Robla *leg.*), 3-VII-2021, on a wooden post of a rock wall, in a rural area (UCO, Ref. Z2770); Oviedo: 1♂ (J. Robla *leg.*), 4-VII-2021, in a house bathroom, at midnight (UCO, Ref. Z2771); Lena, Pola de Lena: 1♀ (*leg.* V. González), 28-II-2022, in a house bedroom (AC). **Pontevedra**: Lalín, Devesa de Arriba: 1♂ + 1♀ (R. Molero *leg.*), 26-VII-1994, in a house (UCO, Ref. Z2703). **Valladolid**: Valladolid: 2 juveniles (R. Molero *leg.*), 22-VII-1994, in a house (UCO, Ref. 2689).

Additional examined material. Specimens studied in Mendes et al. (1994), Molero-Baltanás et al. (1996) and in Molero-Baltanás et al. (2014) from Spain have also been examined, together with some specimens studied in Claus et al. (2022) from Belgium.

Subfamily **Ctenolepismatinae** Mendes, 1991

Genus ***Ctenolepisma*** Escherich, 1905

Ctenolepisma calvum (Ritter, 1910)

Synonyms. *Peliolepisma calva* Wygodzinsky, 1967.

New Records. SPAIN*: **Córdoba*:** Córdoba**:** 5♀ (M. Gaju *leg.*), 7-IX-2021 and 31-X-2023, in the storage room of a house; 2♀ used to start a culture in UCO university and 2♀ mounted on slide (UCO, Ref. Z2764; AMS, K541644). One of the specimens collected in 7-IX-2021 used for genetic analysis (OR732096). This represents the first reliable record of this species for Spain.

Additional examined material. Several specimens from Prague (Czech Republic) studied in Kulma et al. (2022) have also been examined. Some of these specimens are stored in UCO collection (Ref. Z2868).

Ctenolepisma lineatum (Fabricius, 1775)

Synonyms. *Lepisma lineata* Fabricius, 1775; *Lepisma longicornis* de Villers, 1789; *Lepisma vittata* Fabricius, 1798; *Lepisma subvittata* Guérin-Méneville, 1838; *Lepisma annuliseta* Guérin-Méneville, 1838; *Lepisma pilifera* Lucas, 1840; *Lepisma parisiense* Nicolet, 1847; *Forbicina annuliseta* Kolenati, 1858; *Forbicina lineata* Kolenati, 1858; *Forbicina parisiense* Kolenati, 1858; *Forbicina subvittata* Duméril, 1860; *Lepisma quadriseriatum* Packard, 1873; *Lepisma reticulatum* Schött, 1897; *Ctenolepisma quadriseriatum* Escherich, 1905; *Ctenolepisma reticulatum* Escherich, 1905; † *Ctenolepisma lineata* var *pilifera* (Lucas). Papers where specimens have previously been identified as *pilifera* but actually belong to *Ctenolepisma nicoletii* (Lucas, 1846) include Mendes 1978 and several subsequent papers by Mendes and by Molero-Baltanás et al. previous to clarifying the status of *C. nicoletii* (Molero-Baltanás et al. 2012).

New records. SPAIN: Asturias*: Grado, Somines: 2♂ + 1♀ (J. Robla *leg.*), 10-VI-2021, under wooden planks in a granary (UCO, Ref. Z2767; AMS, K541643). One specimen used for genetic analysis (OR732093); 3 juveniles (J. Robla *leg.*), 30-VI-2021, same habitat (UCO, Ref. Z2768); Oviedo: 1♂ (J. Robla *leg.*), 11-VII-2021, on the wall of hall building (UCO, Ref. Z2769).

Additional examined material. All the specimens studied in Molero-Baltanás et al. (2012), from different countries of Europe, have been re-examined.

Ctenolepisma longicaudatum Escherich, 1905

Synonyms. *Lepisma ciliatum dives* Silvestri, 1908; *Ctenolepisma urbana* Slabaugh, 1940; *Ctenolepisma longicaudatum coreanum* Uchida, 1943; *Ctenolepisma pinicola* Uchida, 1964.

New records. AUSTRALIA: Queensland, Chillagoe, Chillagoe Caving Club: 1♂ (D. Mitchell & B. Dane *leg.*), 17-II-2016, building (AMS, K541679). Tasmania, Hobart, Mt.

Stuart, 1 Raymon: 1♀ (S. Bunton *leg.*), 18-VIII-2011, building (AMS, K377675). Specimen used for genetic analysis (MT674899). **SPAIN: Asturias*:** Oviedo, Fernando Vela street: 1♂ + 1♀ (A. Arias *leg.*), 2019, house (BOS); Oviedo, Pedro Masaveu: 2♀ (A. Anadón *leg.*), 2021, house (BOS); Oviedo, Pedro Masaveu: 25♂ + 34♀ + 7 juveniles (C. Lastra *leg.*), 2021-2022, house (BOS); Oviedo: 1♀ (D. Roiz *leg.*), 7-VII-2021, near a carpet in a house bathroom at night (UCO, Ref. Z2765); Oviedo: 1♂ + 1♀ (M. Álvarez *leg.*), 10-VII-2021, on a carpet in a house bedroom (UCO, Ref. Z2766); Pola de Siero, Siero: 1♂ + 1♀ (N. Noval *leg.*), VIII-2022, in a house bedroom (UCO, Ref. Z2867); **Barcelona**: Rubí, in a house, 2♂ + 3♀ (M. Gaju leg.), IV-2010-2017, used for starting a laboratory culture, one female mounted on slide (UCO, Ref. Z2873; AMS, K541555) and one male used for genetic study (OR732097). **Murcia**: Águilas, Calabardina: 1♀ (R. Molero *leg.*), 6-XII-1999, in a hotel (UCO, Ref. Z2307). **CAPE VERDE**: Boavista island, Tamareira: 1♂ + 1♀ (N. López *leg.*), 25-IX-2005 (UCO, Ref. Z2248); Boavista island, Ervatão: 2♂ + 2♀ (N. López *leg.*), 2-X-2005 (UCO, Ref. Z2214). Fogo Island, Achada Furna: 1♂ + 1♀ (P. Oromí *leg.*), 26-I-1999 (UCO, Ref. Z2849). **NAMIBIA**: Swakopmund: 1♀ (M. Gaju *leg.*), 14-VIII-2017, in a hotel room (UCO, Ref. Z2857). **UKRAINE***: Kiev: 2♀ (N. Matushkina *leg.*), 2015 (without concrete date), in a house (UCO, Ref. Z2872).

Additional examined material. All the material studied in Mendes et al. (1994), Molero-Baltanás et al. (1994), Molero-Baltanás et al. (1996) and in Molero Baltanás et al. (2015) from Spain has been re-examined.

Ctenolepisma (Sceletolepisma) villosum (Fabricius, 1775)

Synonyms. *Lepisma villosa* Fabricius, 1775; *Lepisma targionii* Grassi & Rovelli, 1898; *Ctenolepisma targionii* Grassi & Rovelli, 1898.

New records. SPAIN: Albacete*: Alcaraz: 1 juvenile (R. Molero *leg.*), 04-VI-1994, in a hotel (UCO. Ref. Z2668); **Ciudad Real*:** Puertollano, 1♀ (R. Molero *leg.*), 20-II-1995, in a house (UCO. Ref. Z2670). **Córdoba**: in a house, 1 juvenile, 5-V-202 (AMS, K541573). Specimen used for genetic study (OR732092); same locality, 1♂ + 1♀, 20-V-2023, (R. Molero leg.), used for breeding under laboratory conditions (a population of about 30 specimens has been obtained).

Additional examined material. All the specimens identified as *Ctenolepisma targionii* in Mendes et al. (1994), Molero-Baltanás et al. (1992), Molero-Baltanás et al. (1994), Molero-Baltanás et al. (1995) and Molero Baltanás et al. (2015) from Spain, in Molero-Baltanás et al. (2000) from Italy, and in Molero-Baltanás et al. (2018) from Iran have been re-examined.

Ctenolepisma rothschildi (Silvestri, 1907)

Synonyms. *Ctenolepisma diversisquamis* Silvestri, 1908; *Ctenolepisma brachyura* Silvestri, 1918; *Ctenolepisma incita* Silvestri, 1918; *Ctenolepisma reducta* Folsom, 1923.

New records. AUSTRALIA: Queensland, Longreach: 1♂ (G. Smith *leg.*), 12-VIII-2013 (AMS, K261295). Specimen used for genetic analysis (OR732095); Northern Territory, Darwin, two juveniles, 29-VIII-2018, **in a garage,** G. Smith & G. Brown *leg*. UCO Ref. Z2874. **CAPE VERDE:** Boavista, Ribeira de Rabil: 3♂ + 1♀ (N. López *leg.*), 26-IX-2005, under stones (UCO, Ref. Z2220).

Additional examined material. All the specimens listed in Kahrarian et al. (2014) from Iran have been studied.

Genus ***Thermobia*** Bergroth, 1890

Thermobia aegyptiaca (Lucas, 1840)

Synonyms. *Lepisma aegyptiaca* Lucas, 1840; *Thermobia longimana* Escherich, 1905; *Lepisma piliferum* Lucas, 1840; *Thermobia cincta* Wahlgren, 1906; *Thermobia campbelli* Wygodzinsky, 1972; *Ctenolepisma campbelli* Barnhart, 1951;

Additional examined material. All the specimens listed in Kahrarian et al. (2014) from Iran have been studied, together with specimens of *T. aegyptiaca* in Molero-Baltanás et al. (2012) from Egypt.

Thermobia domestica (Packard, 1873)

Synonyms. *Lepismodes inquilinus* Newman, 1863; *Lepisma domestica* Packard, 1873; *Lepisma furnorum* Rovelli, 1884; *Lepismodes furnorum* Bergroth, 1894; *Thermophila furnorum* Sharp, 1894; *Thermobia furnorum* Brown, 1923; *Lepismodes domestica* Kaplin, 1977.

New records. SPAIN: Asturias: Oviedo, Espíritu Santo industrial park: 1♂ + 1♀ + 1 juvenile (J. Robla *leg.*), 8-VII-2021, on bazaar pallets (UCO Ref. Z2763). This represents the second reliable record of this species in Spain. **Sevilla:** Several specimens in a lab culture (J. Robla *leg.*), 2022-2023. One of them (AMS, K541645) used for genetic analysis (OR732103). **ALGERIA***: Tindouf, Dajla, Saharawi refugee camp: 1♂ (D. lópez *leg.*), 2-III-2003 (UCO, Ref. Z2845). **CANADA**: British Columbia**:** Vancouver: 1 juvenile (M. Koch *leg.*), 10-XI-2018, domestic (UCO, Ref. Z2632). **URUGUAY***: Montevideo: 1♀ (C. Morey *leg.*), 1-IX-1960, in a bakery (UCO, Ref. Z2777).

Additional examined material. All the specimens listed in Kahrarian et al. (2014) from Iran, and in Bernal et al. (2022) from Spain, have been studied.

***Thermobia* sp.**

New records. IRAN: Hormozgan, Dargazan Shahbakak village: 1 *ex.* (Seyed Aghil Jaberhashemi*leg.*), 30-VII-2017 (AMS, K541538). Specimen used for genetic analysis (OR732099). Presumably *Thermobia aegyptiaca* but without confirmation due to the bad state of conservation.

Commented list of non-synanthropic silverfish species studied for morphological (and genetic) comparison

Apart from the species indicated in this list, it is likely that some not examined or still not described species of Lepismatidae native to several areas of the world can be confused with the domestic species studied in this work. For example, we have examined an undescribed species of silverfish belonging to the subfamily Heterolepismatinae that has been found in houses of Chile (A. Zúñiga-Reinoso col.) and which shows a habitus that could be confused with young *Lepisma saccharinum* if no accurate identification is carried out.

1) Species related to *Ctenolepisma lineatum*

In this section we include species belonging to the genus *Ctenolepisma* that bear a short subtriangular convex urotergite X and a dorsal pattern of scales with longitudinal bands alternatively dark and light, which are the only characters used for some entomologists to distinguish this species from other Lepismatidae. They also share bearing 3+3 bristle-combs on urotergites II-VII. A closely related group of species, mainly from Macaronesian region, could also be confused, but they show 3+3 bristle combs on urotergites II-VI (the seventh urotergite has only 2+2 bristle combs); this group includes *Ctenolepisma algharbicum*, *C. vieirai*, *C. feae*, etc., and is in need of revision.

1A. *Ctenolepisma almeriense* Molero-Baltanás, Gaju-Ricart & Bach de Roca, 2005

Studied material. All the material studied in Molero-Baltanás et al. (2005) for the original description of this species and in Molero Baltanás et al. (2015) has been re-examined.

Found in anthropic habitats. No data, but probable in south-eastern Spain, where this species seems to be endemic.

Differences with *C. lineatum*. Adults of this species bear only two pairs of styli, have the bristle combs of their thoracic sternites at least in two rows, and have femoral scales more similar to those of *C. nicoletii* (subtriangular, apically truncate).

1B. *Ctenolepisma brauni* Wygodzinsky, 1941

Studied material. Some specimens studied by Mendes (1993) and deposited in MUHNAC.

Found in anthropic habitats. No data, but probable in rural areas of North Africa.

Differences with *C. lineatum*. This species requires revision (and redescription). Mendes (1993) considered this species from North Africa as synonym of *C. lineatum*, interpreting the variability observed in the number of pairs of styli and in the shape of the last abdominal tergite as intraspecific variability. Nevertheless, we have observed in specimens studied by Mendes that they do not match with the characteristics described for the European *C. lineatum* in Molero-Baltanás et al. (2012). It seems more similar to *C. almeriense* considering the chaetotaxy of thoracic sternites and femoral scales, but at least females bear three pairs of styli.

1C. *Ctenolepisma nicoletii* (Lucas, 1846)

New records. SPAIN: Córdoba, 1♂ + 4♀ (R. Molero *leg.*), 26-VIII-2017 (AMS, K541559). 2♀ specimens used for genetic analysis (OR732102 & OR732091).

Additional material studied. All the material presented in Molero-Baltanás et al. (2012) and in Molero Baltanás et al. (2015) from Spain, Portugal, Morocco and Algeria.

Found in anthropic habitats. This species has been found in houses in Spain and Portugal (Molero-Baltanás *et al.* 1996; Mendes 2002, Molero Baltanás *et al.* 2015). These findings can be considered as occasional since they represent less than 1% of the records of this species.

Differences with *C. lineatum*. They were clearly established in Molero-Baltanás et al. (2012). The shape of thoracic sternites (mainly, prosternum), the shape of femoral scales and the number of pairs of styli (always two pairs in adults of *C. nicoletii*) are enough to distinguish both species. Canarian specimens attributed to *C. nicoletii* (and previously to *C. lineatum* or *Lepisma eatoni*) seem to be especially different, which suggests that they could correspond to endemic species (probably more than one) of this archipelago, but this hypothesis requires further revision.

1D. *Ctenolepisma rubroviolaceum* (Schött, 1897)

Studied material. We have not been able to see the types of this species deposited in the California Academy of Sciences, but have clear clues that this species, considered as synonymous of *C. lineatum* by Paclt (1967) and Wygodzinsky (1972), is clearly different to *C. lineatum*. Examining free-living specimens of Arizona and Utah previously identified as *C. lineatum* by Mendes (1992a; 1996), we have detected a lot of differences with the European *C. lineatum* redescribed by Molero-Baltanás et al. (2012).

Found in anthropic habitats. It is possible that this species, native to North America, can enter in houses, and can be confused with *C. lineatum*, that is also present as synanthropic in North America.

Differences with *C. lineatum*. The differences of the native specimens from Arizona and Utah are related to scales shape and distribution on appendages, shape and chaetotaxy of thoracic sternites or pattern of thoracic trichobothria. Probably similar differences can be found in native *Ctenolepisma* that were recorded as *C. rubro-violaceum*. As this species was not described from Arizona or Utah but from California, we do not know if Californian free-living *Ctenolepisma* are conspecific with those from Utah and/or Arizona, but we believe that the authentic *C. lineatum* occurs only in North America as an introduced synanthropic species and the form(s) found in natural habitats of this continent correspond to different taxa.

Some other silverfish from other regions that were previously identified as *C. lineatum* actually do not correspond to this species, such as some those from Cape Verde (Mendes, 1992b), that deserve their designation as a new species in future work.

2. Species related to *Ctenolepisma longicaudatum*

In this section we include species belonging to the genus *Ctenolepisma* that show a trapezoidal tenth urotergite X, uniform dorsal greyish or greyish-brown cover of scales (or a not described pattern of scales) and 3+3 bristle combs on urotergites II-VI.

2A. *Ctenolepisma abyssinicum* Mendes, 1982

Studied material. We have seen one specimen from Abdis Abbeba mounted on slide in Lisbon collection of Centro de Zoologia, prepared by L.F. Mendes, now in MUHNAC.

Found in anthropic habitats. Some specimens were found in a house in Addis Abeba (Ethiopia) according to Mendes (1982).

Differences with *C. longicaudatum*. Adults of species reach a lower size than *C. longicaudatum*, but they could be misidentified with juveniles of *C. longicaudatum* in their distribution area if an accurate study is not carried out observing their chaetotaxy (lower number of macrochaetae per comb) and/or their dorsal scales (heterogeneous in size and structure, some of them paucirradiate as in *C. rothschildi*).

2B. *Ctenolepisma armeniacum* Molero-Baltanás, Gaju-Ricart, Bach de Roca & Mendes, 2010

Studied material. We have studied the holotype of this species and some additional specimens collected in Iran, published in Kahrarian et al. (2016).

Found in anthropic habitats. Although no data of the habitat are available about this species, it could enter accidentally in houses as observed for other species of this group.

Differences with *C. longicaudatum*. The main differences were explained in Molero-Baltanás et al. (2010). The arrangement of macrochaetae of thoracic sternites in two rows is one of the most important. The number of macrochaetae per comb is higher in *C. armeniacum*. The habitus of this species when alive is not known, but probably they are very similar to *C. longicaudatum*, apart from a more intense epidermic pigment.

2C. *Ctenolepisma ciliatum* (Dufour, 1831)

New records. SPAIN: Córdoba: 1♀ (R. Molero *leg.*), 20-IV-2021 (AMS, K541561). Specimen used for genetic analysis (OR732105).

Additional material studied. Most samples from Spain studied in Molero Baltanás et al. (2015), from Italy (Molero-Baltanás et al. 2000) and from Iran (Kahrarian et al. 2014) have been examined.

Found in anthropic habitats. Some specimens of *C. ciliatum* have been found inside houses in Spain and Portugal (Mendes 1978; Molero-Baltanás et al. 1992; 1996) but these records can be considered as accidental since they represent less than 1% of the available records of this species.

Differences with *C. longicaudatum*. Insects belonging to *C. ciliatum* have a more intense epidermic pigment and their dorsal scales are usually more brownish or form 2-4 longitudinal fringes (the pattern is always uniform in *C. longicaudatum*), but as these characters show an important intraspecific variability, the main characters to distinguish between both species require microscopic examination. Most females of *C. ciliatum* have a shorter ovipositor (but there is an important variability of this character in this species), and their thoracic sternites have a different shape: they are more acute, with straight hind margin and a higher number of bristle-combs (see Molero-Baltanás et al. 2010).

2D. *Ctenolepisma iranicum* Molero, Kahrarian & Gaju, 2016

Studied material. The species studied in Molero, Kahrarian and Gaju (2016), from Iran, have been examined.

Found in anthropic habitats. The few samples of this species recorded so far have been found in natural habitats, but it could enter accidentally in houses of its distribution area, as observed for other species of this group.

Differences with *C. longicaudatum*. Molero, Kahrarian and Gaju (2016) discussed the differences between *C. iranicum* and *C. longicaudatum*.

2E. *Ctenolepisma nigrum* (Oudemans, 1890)

Studied material. No specimens belonging to this species have been examined. As its available descriptions are not updated and the description given by Silvestri (1913) actually corresponds to *C. rothschildi* (Irish 1995), *C. nigrum* requires a thorough revision.

Found in anthropic habitats. Considering that some records of this species could be misidentified with C. rothschildi, no clear information about the habitat of this species is available. It is possible that *C. nigrum* can occur accidentally as domestic.

Differences with *C. longicaudatum*. Irish (1995) revised some characters of *C. nigrum*, clarifying that this species bears 3+3 combs on urotergites II-VI, which clearly distinguish it from *C. rothschildi*. Compared to *C. longicaudatum*, it is smaller, with a lower number of macrochaetae in their combs and more intense epidermic pigment. Dorsal scales are blackish or greyish black if we follow its original description, but the variability of this species is not assessed.

3. Species related to *Ctenolepisma calvum* and *Ctenolepisma rothschildi*

In this section we include species belonging to the genus *Ctenolepisma* that show a trapezoidal tenth urotergite X, uniformly coloured cover of scales (light or with variable pigmentation, or pattern of scales not described), 3+3 bristle combs on urotergites II-V (2+2 in urotergites VI-VIII) and absence of median combs on urosternites. Most of these species require revision, but characters are presented coming from their most updated descriptions to show their differences with the *C. calvum* and *C. rothschildi*. The characters for distinguishing between these two species are presented in the identification key of the main document of this work.

3A. *Ctenolepisma guineense* Mendes, 1985

Studied material. One paratype of this species has been examined in the collection of L.F. Mendes, now in MUHNAC.

Found in anthropic habitats. No data.

Differences with *C. calvum*. Both sexes of *C. guineense* bear two pairs of abdominal styli. The number of macrochaetae per comb is, in general, clearly higher in this African species (for example, posterior combs of nota with 4 macrochaetae, infralateral combs of urotergites with 4-11 macrochaetae, etc.). The labial palp has 5 papillae and the ovipositor is clearly longer than in *C. calvum*.

Differences with *C. rothschildi*. Dorsal scales of *C. guineense* are not heterogeneous according to the original description of Mendes (1985), but we have seen pauci-radiate scales in the paratype. This author also indicates that both species are different in the relative length and width of the articles of maxillary and labial palps, although these characters frequently show instraspecific variability. Considering this, the species *C. guineense* should be revised to find additional differences or synonymized with *C. rothschildi*.

3B. *Ctenolepisma nigericum* Mendes, 1982

Studied material. One specimen of this species was examined in the collection of L.F. Mendes, now in MUHNAC.

Found in anthropic habitats. The few known specimens of this species are collected in natural habitats.

Differences with *C. calvum*. *C. nigericum* has a clearly longer urotergite X, a higher number of macrochaetae in its combs, and more intense epidermic pigment in its appendages.

Differences with *C. rothschildi*. Following the key presented by Mendes (1982), *C. nigericum* has a shorter ovipositor that does not surpass the apex of styli IX, the inner process of the coxite IX has a higher ratio length/width. The dorsal cover of scales seems to be not clearly heterogeneous. The maximum body length of *C. nigericum* is higher.

3C. *Ctenolepisma submagnum* Silvestri, 1908

Studied material. Two specimens (male and female) from **Guinea** (Conakry), Mount Nimba, Zougué valley (N. Matushkina *leg.*), 5-X-2011 (UCO, Ref. Z2855).

Found in anthropic habitats. No data. Apparently found only in natural habitats.

Differences with *C. calvum*. Both sexes of *C. submagnum* bear three pairs of styli, and *C. calvum* bears only one pair. The ovipositor is clearly longer and appendages have intense pigment in *C. submagnum*. The apical article of the labial palp has five papillae (a lower number in *C. calvum*).

Differences with *C. rothschildi*. Both sexes of *C. submagnum* bear three pairs of styli, and *C. rothschildi* bears only two pairs. The shape of the urotergite X of *C. submagnum* is more subtriangular and not clearly trapezoidal as in *C. rothschildi*. The metasternum of *C. subgmagnum* has 2+2 combs of macrochaetae, and only 1+1 combs in *C. rothschildi*.

3D. *Ctenolepisma targionianum* Silvestri, 1908

Studied material. One specimen from Kenya (studied by L.F. Mendes and deposited in MUHNAC) and several specimens from Venezuela (deposited in UCO collection) have been studied. Although there are some similarities between African and American populations, some differences have been found suggesting that this species requires revision. Anyway, both American and African specimens are clearly different from synanthropic species.

Found in anthropic habitats. No data. Specimens studied by Mendes (1982) were found in natural habitats.

Differences with *C. calvum*. Both sexes of *C. targionianum* bear three pairs of styli, and *C. calvum* bears only one pair. The ovipositor is clearly longer and appendages have intense pigment in *C. targionianum*. The apical article of the labial palp has five papillae (a lower number in *C. calvum*).

Differences with *C. rothschildi*. Both sexes of *C. targionianum* bear three pairs of styli, and *C. rothschildi* bears only two pairs. The dorsal scales of *C. targionianum* seem to be of uniform appearance.

4. Species related to *Ctenolepisma (Sceletolepisma) villosum*

In this section we include species belonging to the genus *Sceletolepisma* that show a trapezoidal tenth urotergite X, uniformly coloured cover of scales (light or dark greyish, sometimes blackish, or pattern of scales not described) and presence of median combs on urosternites (this character should be detected to avoid misidentification with species included in the previous section). All species indicated below show 3+3 bristle combs on urotergites II-V (2+2 in urotergites VI-VIII), with the exception of *C. plusiochaetum* Silvestri, 1922. Some of the species of this group require revision, but characters are presented coming from their most updated descriptions to show their differences with *C. villosum*.

4A. *Ctenolepisma (Sceletolepisma) corvinum* Silvestri, 1908

Studied material. The holotype of this species has been examined (loaned by the Berlin Museum für Naturkunde, collection number SESAM-Kat.Nr:31-Z). It is a female in poor condition and most diagnostic characters of the species are not well preserved in this specimen. The characters supporting its inclusion in the subgenus *Sceletolepisma* are still sufficiently visible. Fortunately, Irish (1987) presented a more updated redescription using additional material.

Found in anthropic habitats. This species has not been found as synanthropic.

Differences with *C. villosum*. Males of *C. corvinum* bear two pairs of styli, those *of C. villosum* have only one pair. The urosternite VII of *C. corvinum* bears 1+1 lateral combs and one median comb; the same urosternites in *C. villosum* lacks median comb.

4B. *Ctenolepisma (Sceletolepisma) decellei* Mendes, 1982

Studied material. No specimens of this species have been studied.

Found in anthropic habitats. The only specimen used for the description of this species was apparently found in natural habitat (Tibesti Mountains, Chad).

Differences with *C. villosum*. The male of *C. decellei* has two pairs of styli and adult males of *C. villosum* have only one pair. The metasternum of *C. decellei* has 2+2 combs of macrochaetae and the same sternite in *C. villosum* has only 1+1 combs.

4C. *Ctenolepisma (Sceletolepisma) dewittei* Mendes, 1982

Studied material. No specimens of this species have been examined.

Found in anthropic habitats. No data

Differences with *C. villosum*. As *C. dewittei* is related to *C. corvinum*, differences with *C. villosum* are similar: the male of *C. dewittei* has two pairs of styli and adult males of *C. villosum* have only one pair. The urosternite VII of *C. dewittei* bears 1+1 lateral combs and one median comb; the same urosternite in *C. villosum* lacks median comb.

4D. *Ctenolepisma (Sceletolepisma) lociplanum* Irish, 1996

Studied material. No specimens have been examined.

Found in anthropic habitats. All specimens collected were found in natural habitats, according to Irish (1996).

Differences with *C. villosum*. The urosternite VII of *C. lociplanum* bears 1+1 lateral combs and one median comb; the same urosternite in *C. villosum* lacks median

comb. The inner process of the coxite IX in *C. lociplanum* has a transverse comb of macrochaetae, which is absent in *C. villosum*.

4E. ***Ctenolepisma (Sceletolepisma) namibense*** Irish, 1987

Studied material. No specimens belonging to this species have been examined.

Found in anthropic habitats. Not found so far.

Differences with *C. villosum*. The urotergite I of *C. namibense* has 2+2 bristle combs, while this urotergite has only 1+1 combs in *C. villosum*. The inner process of the coxite IX in *C. namibense* has a transverse comb of macrochaetae, which is absent in *C. villosum*.

4F. ***Ctenolepisma (Sceletolepisma) ossilitorale*** Irish, 1987

Studied material. No specimens have been studied for this work.

Found in anthropic habitats. This species has been found in desert coasts of Namibia, in sandy habitat, not in domestic environments.

Differences with *C. villosum*. The urosternite VII of *C. ossilitorale* bears 1+1 lateral combs and one median comb; the same urosternite in *C. villosum* lacks median comb. The metasternum of *C. ossilitorale* has 2+2 combs of macrochaetae and the same sternite in *C. villosum* has only 1+1 combs. Females of *C. ossilitorale* bear only one pair of abdominal styli, but females of *C. villosum* have two pairs.

4G. ***Ctenolepisma (Sceletolepisma) pauliani*** Wygodzinsky, 1959

Studied material. No specimens belonging to this species have been examined.

Found in anthropic habitats. *C. pauliani* has been found only in natural habitats (sandy dunes).

Differences with *C. villosum*. The urosternite I of *C. pauliani* has one median comb of macrochaetae, but this urosternites lacks combs in *C. villosum*. The urosternites II of *C. pauliani* bears 1+1 lateral combs and one median comb; the same urosternite in *C. villosum* has only a median comb (the lateral combs are absent). When intact, antennae and cerci of *C. pauliani* are more than twice longer than body length, while in *C. villosum* these appendages are (at most) as long as body length, although usually a little shorter.

4H. ***Ctenolepisma (Sceletolepisma) penrithae*** Irish, 1987

Studied material. No material of this species has been examined for this study.

Found in anthropic habitats. This species has been found only in natural habitats (so far).

Differences with *C. villosum*. The urosternite VII of *C. penrithae* bears 1+1 lateral combs and one median comb; the same urosternite in *C. villosum* lacks median comb. Males of *C. penrithae* have two pairs of styli and those of *C. villosum* have only one pair. The epidermic pigment is darker in *C. penrithae*. The ovipositor of *C. villosum* is clearly longer than in *C. penrithae* (compared to styli IX).

4I. ***Ctenolepisma (Sceletolepisma) plusiochaetum*** **Silvestri, 1922**

Studied material. No specimens belonging to this species have been examined.

Found in anthropic habitats. This species has been occasionally found entering houses in Windhoek, according to Irish (1987), but its usual habitat is dry savanna and desert in southwestern Africa.

Differences with *C. villosum*. Living specimens are shiny black. Apart from this distinctive color, this species has a unique chaetotaxy. Urotergites II-VIII have 3+3 combs of macrochaetae (urotergites VI-VIII bear only 2+2 combs in *C. villosum*), and median combs are present in urosternites II-VII (the urosternite VII lacks median comb in *C. villosum*).

4J. *Ctenolepisma sanctahelenae* Wygodzinsky, 1970

Studied material. No specimens belonging to this species have been examined.

Found in anthropic habitats. Not found so far in domestic environments.

Differences with *C. villosum*. The species endemic to the Santa Helena Island has one pair of styli in both sexes; in *C. villosum* females bear two pairs of styli. The ovipositor of *C. sanctahelenae* has a sclerotized apical projection which is absent in *C. villosum*. The urosternite VII of *C. sanctahelenae* bears 1+1 lateral combs and one median comb; the same urosternite in *C. villosum* lacks median comb. The metasternum of *C. sanctahelenae* has 2+2 combs of macrochaetae and the same sternite in *C. villosum* has only 1+1 combs.

4K. *Ctenolepisma sanctithomae* Mendes, 1993

Studied material. One paratype of this species has been examined in the collection of L.F. Mendes, now in MUHNAC.

Found in anthropic habitats. The few specimens collected of this species were found in natural habitat.

Differences with *C. villosum*. In the specimen examined tibial scales have been detected, which is a character never previously described in the subgenus *Sceletolepisma* (all examined species, included *C. villosum*, have only setae on tibiae). Apart from this new character for this species, the urosternite VII of *C. sanctithomae* bears 1+1 lateral combs and one median comb; the same urosternite in *C. villosum* lacks a median comb. Males of *C. sanctithomae* have two pairs of styli and those of *C. villosum* have only one pair. The metasternum of *C. sanctithomae* has 2+2 combs of macrochaetae and the same sternite in *C. villosum* has only 1+1 combs.

4L. *Ctenolepisma spinipes* Irish, 1987

Studied material. No material of this species has been examined for this study.

Found in anthropic habitats. Not found so far in this type of habitat.

Differences with *C. villosum*. The urotergite I of *C. spinipes* has 2+2 bristle combs, while this urotergite has only 1+1 combs in *C. villosum*. *C. spinipes* has a very short ovipositor that at most reaches the base of the styli IX, very much longer in *C. villosum*. Females of *C. spinipes* have one pair of styli (two pairs in *C. villosum*).

4M. *Ctenolepisma subterebrans* Irish, 1987

Studied material. No specimens studied for this work.

Found in anthropic habitats. Only collected in natural habitats.

Differences with *C. villosum*. The urosternite VII of *C. subterebrans* bears 1+1 lateral combs and one median comb; the same urosternite in *C. villosum* lacks median comb. *C. subterebrans* has a short ovipositor that does not clearly surpass the apex of the styli IX; in females of *C. villosum* the apical part of the ovipositor is clearly longer. Females of *C. subterebrans* have one pair of styli (two pairs in *C. villosum*).

4N. *Ctenolepisma tanzanicum* Mendes, 1982

Studied material. The only specimen (holotype) used for the description of this species has not been examined.

Found in anthropic habitats. No. Collected only on one occasion in rotten wood.

Differences with *C. villosum*. The male of C. *tanzanicum* has two pairs of styli, while *C. villosum* has only one pair. The urosternite VII of *C. tanzanicum* bears 1+1 lateral combs and one median comb; the same urosternite in *C. villosum* lacks median comb.

4O. *Ctenolepisma terebrans* Silvestri, 1908

Studied material. Some specimens labelled as "Co-Typus" belonging to this species have been examined (loaned by the Berlin Museum für Naturkunde, collection number SESAM-Kat.Nr:57-Z). They are in poor condition and with broken abdomens, but most of their diagnostic characters have been checked, agreeing with the redescription given by Irish (1987). All scales on femora seem to be rounded and tibiae lack scales as observed in most species examined of the subgenus *Sceletolepisma*.

Found in anthropic habitats. Not collected out of natural habitats, but as some specimens have been observed entering into termite and ant colonies, it is likely that they could enter in buildings if they have the possibility.

Differences with *C. villosum*. Males of *C. terebrans* bear two pairs of styli and in C. villosum they bear only one pair. Females of *C. terebrans* have a sclerotized apex of the posterior gonapophyses of their ovipositor, which is not observed in C. villosum. The urosternite VII of *C. terebrans* bears 1+1 lateral combs and one median comb; the same urosternite in *C. villosum* lacks median comb.

5. Species related to *Lepisma saccharinum*

Although a lot of silverfish species have been confused with *Lepisma saccharinum* (see **Supplementary Material 4**), in this section we only included species of Lepismatinae with isolated dorsal macrochaetae, greyish or greyish brown uniform dorsal scales, similar fusiform body shape and head chaetotaxy, and long trapezoidal urotergite X (about as long as wide in the base or even higher in adults). These species cannot be distinguished from *Lepisma saccharinum* in usual photographs in dorsal view, but they are usually free-living and restricted to some areas of West Palaearctic.

5A. *Neoasterolepisma inexpectatum* Mendes, Molero-Baltanás, Bach de Roca & Gaju-Ricart, 1993

Studied material. All the specimens used for the original description of this species have been examined.

Found in anthropic habitats. This species seems to be associated with the litter of the laurisilva forest of the islands of Tenerife and Gran Canaria, but in these islands, it could enter in houses and be misidentified as *Lepisma saccharinum* because it has a similar appearance (silvery greyish uniform dorsal scales, similar shape of the tenth urotergite, etc.). Some *Neoasterolepisma* are associated with ant colonies and have yellowish colour, but in the Canarian archipelago most silverfish belonging to this genus are greyish and only occasionally found with ants.

Differences with *Lepisma saccharinum*. Differences between the genera *Neoasterolepisma* and *Lepisma* genera are based on microscopic characters. *Lepisma* species have closed posterior trichobothrial areas in the pronotum (see Supp. 2, Fig. S15) and *Neoasterolepisma* species have open posterior trichobothrial areas in this sclerite. The antennae of *Lepisma* have a special type of globose basiconic sensillum (type F or Silvestri's sensillum) and, in the same positions, *Neoasterolepisma* has a specialized type with thin branches called asteriform sensillum. The paramera of males in *Lepisma* are hyperdeveloped (Fig. 9), reaching or surpassing the apex of the coxite IX, and medium-sized in *N. inexpectatum*, about half the length of the coxite IX and clearly not reaching its apex. The number of macrochaetae of urosternal combs in *N. inexpectatum* is slightly lower than in *L. saccharinum*.

5B. *Neoasterolepisma myrmecobium* (Silvestri, 1908)

Studied material. All the specimens studied in Mendes et al. (1992) and in Mendes et al. (1993) from Canary Islands have been examined.

Found in anthropic habitats. This species is occasionally associated to ants, but most records correspond to free-living silverfish collected in several Atlantic archipelagos (Madeira, Canary Islands, Cape Verde). There are no records of this species as domestic, but it could enter in buildings in its area of this distribution. The species has been cited in geographic areas very far from its original areas, such as Peru (Silvestri, 1940). If these records do not correspond to misidentifications, we can interpret that *N. myrmecobium* has been accidentally introduced, which suggests dispersal with material transported by humans and occasional association with anthropic habitats.

Differences with *Lepisma saccharinum*. This species has been previously misidentied with *Lepisma saccharinum* and with other Lepismatinae such as *N. curtiseta*. For example, Mendes (1988) corrected one identification made by Paclt (1966). probably because it is very similar at first sight (body shape, urotergite X shape, silvery greyish dorsal color, etc.). Microscopic differences between the genera *Neoasterolepisma* and *Lepisma* genera are indicated in the previous section on *N. inexpectatum*. Moreover, the infralateral groups of urotergites in *N. myrmecobium* bear only one macrochaeta with an outer thin seta (although the lateral macrochaeta is very close to the infralateral), while in *L. saccharinum* these infralateral groups have two macrochaetae and an outer thin seta. The number of macrochaetae of urosternal combs in *N. myrmecobium* is slightly lower than in *L. saccharinum*. The hind margin of the urotergite X in *N. myrmecobium* is slightly concave and more straight in *L. saccharinum*.

6. Species related to *Thermobia aegyptiaca* and *T. domestica*

The genus *Thermobia* requires revision. The status of *T. infelix* Silvestri, 1907 is uncertain and some specimens attributed to *T. domestica* could correspond to different species. Two species were described in the revision of Irish (1988), but they have been found only in South Africa.

6A. *Thermobia nebulosa* Irish, 1988

New records. NAMIBIA: Luderitz, Shark Island: 1♀ + 1 *ex.* (J. Iris *leg.*), 19-VI-2017 (AMS, K541577). Both specimens used for genetic analysis (OR732094 & OR732098).

Found in anthropic habitats. No. Only collected in desertic in natural habitats.

Differences with *T. aegyptiaca* and *T. domestica*. This species is clearly different to synanthropic species, both with Palaearctic origin, because it shows a clearly sclerotized apex of the ovipositor. Other differences are related to the shape and chaetotaxy of thoracic sternites, designed in Irish (1988).

6B. *Thermobia vallaris* Irish, 1988

Studied material. NAMIBIA: Kaukausib fountain, 2♂ + 5♀, 27/03/1982, J. Irish *leg*. paratypes designed by Irish (1988) deposited in MUHNAC (except 1♀ studied by SEM in UCO).

Found in anthropic habitats. No. Collected in natural habitats, mainly under stones.

Differences with *T. aegyptiaca* and *T. domestica*. This species is clearly different to both synanthropic species because its ovipositor is sclerotized apically, as in *T. nebulosa*. Moreover, there are additional differences in thoracic sternites shape and chaetotaxy. In a preliminary revision of one specimen of *T. vallaris* we have detected truncated scales on femora; this modified type is absent in Palaearctic species.

References

Bernal I, Molero-Baltanás R, Viejo JL. 2022. First record of the synanthropic species *Thermobia domestica* (Packard, 1837) (Zygentoma: Lepismatidae) in Spain. Boletín Sociedad Entomológica Aragonesa. 71:153-154.

Claus R, Vantieghem P, Molero-Baltanás R, Parmentier T. 2022. Established populations of the indoor silverfish *Lepisma saccharinum* (Insecta: Zygentoma) in red wood ant nests. Belg. J. Zool. 152:45–53.

Irish J. 1987. Revision of the genus *Ctenolepisma* Escherich (Thysanura: Lepismatidae) in southern Africa. Cimbebasia (A). 7(11):147–207.

Irish J. 1988. Revision of *Thermobia* Bergroth (Thysanura: Lepismatidae). Cimbebasia. 10:15-30.

Irish J. 1995. New data on Lepismatidae, mainly from Italy and north east Africa, with notes on the status of *Ctenolepisma rothschildi* Silvestri (Insecta: Thysanura). Ann. Mus. Civ. Stor. Nat. Giacomo Doria. 90:559–570.

Irish J. 1996. Lepismatidae from North-East Namibia, including the first record of *Namunukulina* Wygodzinsky from subequatorial Africa (Insecta: Thysanura). Mitteilungen aus dem Zoologischen Museum in Berlin. 72(1):15–19.

Kahrarian M, Molero–Baltanás R, Reza M, Shanavaee MR. 2014. A faunistic study on Lepismatidae (Zygentoma) in Kermanshah (Iran). Entom. Gen. 35:53–60.

Kahrarian M, Molero–Baltanás R, Gaju M. 2016. The genus *Ctenolepisma* (Zygentoma: Lepismatidae) in Western Iran, with description of three new species. Zootaxa. 4093(2):217–230.

Kulma M, Molero-Baltanás R, Petrtýl M, Patoka J. 2022. Invasion of synanthropic silverfish continues: first established populations of *Ctenolepisma calvum* (Ritter, 1910) revealed in the Czech Republic. BioInvasions Records. 11(1):110–123

Mendes LF. 1978. Nota sobre o género *Ctenolepisma* (Zygentoma, Lepismatidae) em Portugal. Arq. Mus. Boc. Lisbon. 6(2):279–298.

Mendes LF. 1982. Notas e descrições de Lepismatidae afrotropicais (Zygentoma: Apterygota) II. Géneros *Ctenolepisma* e *Namunukulina*. Revue Zoologique Africaine (Bruxelles). 93(3):591–661.

Mendes LF. 1985. Notas e descriçoes de Lepismatideos afrotropicais (Zygentoma, Apterygota). III. Tisanuros da Guine-Bissau. Boletim da Sociedade Portuguesa de Entomologia. 72:1–33.

Mendes LF. 1988. Revisão do género *Lepisma* Lin., 1758 *s.lat*. (Zygentoma, Lepismatidae). Boletim da Sociedade Portuguesa de Entomologia (Suppl.). 2:1–236.

Mendes LF. 1992a. Novos dados sobre tisanuros (Microcoryphia e Zygentoma) da América do Norte. Garcia de Orta, Séries Zoologia, Lisboa. 16(1-2):171–193.

Mendes LF. 1992b. Nova contriuicão para conhecimento dos Thysanura (Microcoryphia e Zygentoma: Insecta) da República Democratica de Cabo Verde. Garcia de Orta, Séries Zoologia, Lisboa. 12(1-2):225–233.

Mendes LF. 1993. New data on the thysanurans (Microcoryphia and Zygentoma: Insecta) from Northern Africa and from the Near East. Garcia de Orta, Séries Zoologia, Lisboa. 18(1-2):79–93.

Mendes LF. 1996. Some new data on the Microcoryphia and Zygentoma (Insecta) from the United States. Garcia de Orta, Séries Zoologia, Lisboa. 21(1):117–126.

Mendes LF. 2002. Tisanuros (Microcoryphia e Zygentoma: Insecta) de Portugal. Novos dados e considerações. Comunicações do Instituto de Investigação Científica Tropical. 3:9-47.

Mendes LF, Bach de Roca C, Gaju-Ricart M. 1992. New data on the thysanurans fauna of the Canary islands. I. Zygentoma. Garcia de Orta, Séries Zoologia, Lisboa. 16(1-2):195–203.

Mendes LF, Bach de Roca C, Gaju-Ricart M & Molero-Baltanás R. 1994. *Trichotriuroides boneti* gen. et sp. n (Zygentoma, Nicoletiidae) and new data on Zygentoma in the collection of the Museo Nacional de Ciencias Naturales in Madrid (Spain). EOS. Revista Española de Entomología. 69:21–29

Mendes LF, Molero–Baltanás R., Bach de Roca C, Gaju–Ricart M. 1993a. Novos dados sobre a fauna de tisanuros das ilhas Canariás. II. Zygentoma. Notas e descrições de três novas espéces. Garcia de Orta, Séries Zoologia, Lisboa. 16(1–2):195–203.

Molero R, Tahami MS, Gaju M, Sadehgi S. 2018. A survey of basal insects (Microcoryphia and Zygentoma) from subterranean environments of Iran, with description of three new species. ZooKeys. 806:17-46.

Molero-Baltanás R, Bach De Roca C, Gaju-Ricart M. 1992. Los Zygentoma de Andalucía (Insecta: Apterygota). Zool. Baetica. 3:93-115.

Molero–Baltanás R, Fanciulli PP, Frati F, Carapelli A, Gaju–Ricart M. 2000. New data on the Zygentoma (Insecta, Apterygota) from Italy. Pedobiologia. 44:320–332.

Molero-Baltanás R, Gaju-Ricart M, Bach de Roca C, Mendes LF. 1994. New faunistic data on the Lepismatidae of Spain. Acta Zool. Fenn. 195:107-110.

Molero-Baltanás R, Gaju-Ricart M, Bach de Roca C. 1996. Los Lepismatidae antropófilos de España. Boletín RSEHN. 125:178-181.

Molero-Baltanás R, Gaju-Ricart M, Bach de Roca C. 2005. *Ctenolepisma almeriensis* n. sp. of Lepismatidae (Insecta, Zygentoma) from south–eastern Spain. Anim Biodiv Conserv. 28(1):91–99.

Molero-Baltanás R, Gaju-Ricart M, Bach de Roca C, Mendes, LF. 2010. On *Ctenolepisma ciliata* and a new related species, *Ctenolepisma armeniaca* sp.n. (Zygentoma, Lepismatidae). Deutsche Entomologische Zeitschrift. 57(2):243-252.Molero Baltanás R, Gaju Ricart M, Bach de Roca C. 2012. New data for a revision of the genus *Ctenolepisma* (Zygentoma: Lepismatidae): redescription of *Ctenolepisma lineata* and new status for *Ctenolepisma nicoletii*. Ann Soc Entomol Fr. 48(1–2):66–80.

Molero Baltanás R, Gaju Ricart M, Bach de Roca C. 2014. Lepismas y libros: actualización del conocimiento sobre *Lepisma saccharina* (Zygentoma: Lepismatidae) en España. Boletín Sociedad de Entomología Aragonesa. 54:351-357.

Molero Baltanás R, Gaju Ricart M, Bach de Roca C. 2015. Actualización del conocimiento del género *Ctenolepisma* Escherich, 1905 (Zygentoma, Lepismatidae) en España peninsular y Baleares. Boletín Asociación Española de Entomología. 39(3–4):365–390.

Paclt J. 1966. Neue Beiträge zur Kenntnis der Apterygoten-Sammlung des Zoologischen Staatsinstituts und Zoologischen Museums Hamburg. II. Lepismatidae und Maindroniidae. Entomologishe Mitteilungen aus dem Zoologischen Museum Hamburg. 157:147–162.

Paclt J. 1967. Thysanura. Fam. Lepidotrichidae, Maindroniidae, Lepismatidae. Gen. Ins. 218e:1–86.

Silvestri F. 1913. On some Thysanura in the Indian Museum. Rec. Indian Museum. 9:51–62.

Silvestri F. 1940. Primo contributo alla conoscenza dei Tisanuri del Peru e descrizione di un genere e due specie dell'Argentina settentrionale. Bollettino del Laboratorio di Entomologia e Agraria della Facoltà Agraria in Portici. 4:444–458.

Wygodzinsky P. 1955. Thysanura. Commentationes Biologicae. 15(11):1–4.

Wygodzinsky P. 1972. A review of the silverfish (Lepismatidae, Thysanura) of the United States and the Caribbean area. Am. Mus. Novit. 2481:1–26
