# Supplementary material for: Worldwide revision of synanthropic silverfish (Insecta: Zygentoma: Lepismatidae) combining morphological and molecular data
Source: J Insect Sci. 2024 May 4;24(3):1. doi: 10.1093/jisesa/ieae045 (PMC11069193; doi:10.1093/jisesa/ieae045)
Supplement: ieae045_suppl_Supplementary_Materials_2 [file ieae045_suppl_supplementary_materials_2.pdf]

Supplementary Material 2. Morphological characters used for Lepismatidae taxonomy, species diagnosis and glossary[1].

This document includes illustrated descriptions of the most important characters and terminology used in Zygentoma taxonomy and a detailed glossary of Zygentoma morphology. Illustrations are **a**) Scanning Electron Microscopy (SEM) photographs; **b)** drawings made with a camera lucida attached to a light microscope; and **c)** microphotographs taken with light microscope if considered useful. Note that not all morphological characters used for Lepismatidae taxonomy and phylogeny are included in this document, but only those that are interesting for diagnosis and identification of synanthropic silverfish of the world. Most of these characters have been used and discussed by previous Zygentoma experts (e.g., P. Wygodzinsky, L.F. Mendes, G. Smith, R. Molero-Baltanás and J. Irish). Some additional useful references are the description of the general anatomy of a Lepismatidae (Denis 1949; Molero-Baltanás et al. 2015, Mendes 2018), the unpublished PhD Thesis of J. Irish (Irish, 1990) and an ongoing morphological taxonomy work of Lepismatidae (Molero-Baltanás et al. under preparation). Some of the characters that are mentioned but not explained here will be detailed and discussed in that work. An extensive glossary is also provided at the end of this document. Figures presented in the main text of the manuscript (and not here) are referred with the number used there (e.g, Fig. 1, 2, 3), and figures only included in this Supplementary material are referred with an 'S' (e.g., Fig. S1, S2, S3). For facilitating navigation across the document, enable the navigation panel in view.

[1]Supplementary document to the paper «**Worldwide revision of synanthropic silverfish (Insecta: Zygentoma, Lepismatidae) combining morphological and molecular data» (2024)**. By Rafael Molero-Baltanás, Andrew Mitchell, Miquel Gaju-Ricart & Jairo Robla in *Journal of Insect Science.*

1) Colour, size and body shape

Epidermic pigmentation. Subterranean species and most synanthropic species have light epidemic pigment, even absent, compared with other species that can be more active at the surface of the ground during the daytime hours. Some free-living species have more intense epidermic pigmentations than their synanthropic relatives (example: *C. ciliatum* compared to *C. longicaudatum*). Although it is useful to describe this character, a variability has been detected for species where enough specimens have been examined, so this trait should not be used in most cases as a key character.

Scales color pattern. This character provides some clues for the identification of living specimens just in the moment where they are found, but it is not diagnostic at the species level for most Lepismatidae. The pattern of *Thermobia domestica* with dark and light pigmented fringes (especially transverse bands) is very characteristic, but *T. aegyptiaca* has a similar pattern and both species have been confused with some *Ctenolepisma* species, such as *C. lineatum* or free-living related species. Several species show parallel longitudinal lines of scales alternating dark and light colors, but the contrast between these lines is variable depending on the moulting stage, becoming less evident in more uniformly dark colored specimens that are close to molting. This is the same problem with species with uniform greyish pattern, as C. *longicaudatum* or *C. villosum*: some of them have been described as having dark greyish or silvery greyish dorsal scales, but sometimes specimens are found with light greyish scales (a specimen belonging to *C. longicaudatum* from Saragossa, Spain, was covered by whitish, almost transparent scales, as well as another one from Cordoba belonging to *C. villosum*; **Figs**. 28B and 28D). Even in specimens with dark scales, these can be partially or totally lost, and the general color becomes not so dark, leading to confusions with other species with a lighter cover of scales, such as *C. calvum*. Moreover, juveniles are usually lighter than adults, so a young specimen of *C. villosum* can be confused with a subadult of *C. calvum*. Moreover, the pattern of other species has not been described since they have been studied only with dead specimens preserved in alcohol and some others, as *C. rothschildi*, seem to have a variable pattern, at least if we trust in the different descriptions that have been given for different synonyms of this species.

Body shape. Most silverfish (including all synanthropic taxa) have a fusiform body shape. In some species the thorax is wider than the abdomen and stands out clearly from abdomen base, but in some other this difference of width is less marked. The ratio length/width of the body can be diagnostic in some cases. Nevertheless, precise differences cannot be established since the shape can be variable in living specimens depending on physiological state (gravid females have bigger abdomens, specimens in dry environments can show shorter abdomens because partial dehydration process, etc.) and also in dead specimens preserved in alcohol because differences in the expansion of membranes separating the different body segments.

Size. Although the length of the body, antennae and caudal filaments (those of appendages, relative to body length) can be useful to distinguish species, silverfish are ametabolous and grow during their entire life. Some species can reach a greater or longer maximum body length than others, but the size of young specimens of most species overlap; for example, *Ctenolepisma longicaudatum* and *Acrotelsa collaris* can have about 16-18 mm (or perhaps even more) of body length, but most adults with lower body size

have similar length to adults of other species such as *C. targionii/villosum* or *C. calvum*. Moreover, their appendages are frequently broken. So, although it is advisable to include the maximum body length and the maximum observed for antennae and caudal filaments in descriptions of species, it is not recommended to use them in an identification key.

2) Types of macrochaetae

Genera of Lepismatidae included in the subfamilies Lepismatinae and Heterolepismatinae have **smooth and apically bifid macrochaetae** (**Fig**. 6A). This type is very likely the more plesiomorphic in Zygentoma since the remaining families of this order, including those considered more primitive, show it. Other silverfish present **feathered (=pectinate or plumose)** macrochaetae, i.e., with their surface covered with some numerous short and small barbs or pectinations. There are mainly two types of plumose macrochaetae; **type 1** (**Figs**. 6B and S1) has one or two rows of pinnulae concentrated in one side of the macrochaeta (see Mendes 1988a) and **type 2** (**Fig**. S2) has a lot of small barbs surrounding all the surface of the macrochaeta. In some species, several subtypes of these macrochaetae can be distinguished in different parts of the same insect.

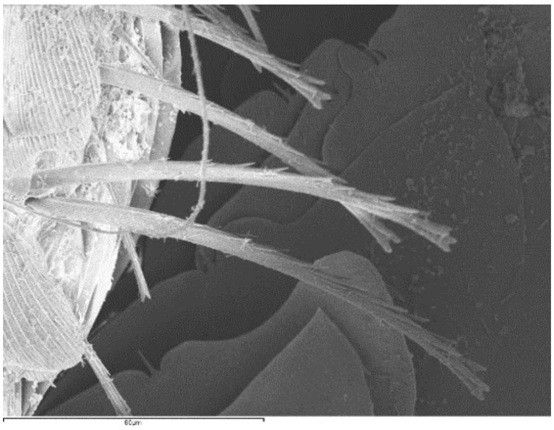

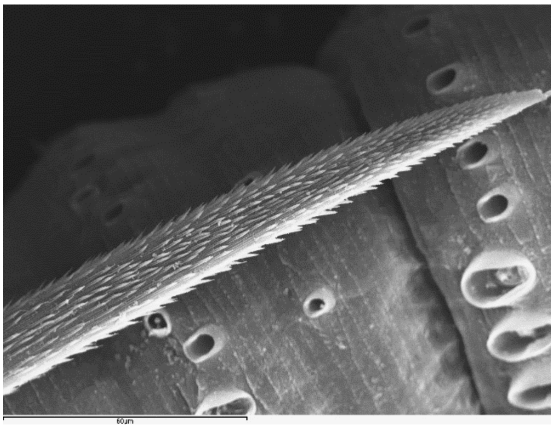

Fig. S1 (Left). Feathered macrochaetae, Type 1 of *Ctenolepisma* sp. (Venezuela). **Fig. S2** (Right). Feathered macrochaetae, Type 2 of *Acrotelsa collaris* (Cape Verde).

3) Types of scales covering the body

Most scales covering the body of silverfish are **rounded orbicular**, i.e., with backwards directed lateral prolongations of their basal surface surrounding their socket (**Fig**. S3). Their surface presents a lot of **rays** (= **ribs**) that are usually parallel, but they can be crossed in some parts; these rays can be more densely distributed or more spaced (separated). The size of scales can be variable, but ventral scales are in most species relatively uniform (**Fig**. 13A, S4); dorsal scales are more heterogeneous and in some species of *Ctenolepisma* some thoracic scales are larger and with a lower number of rays, greatly separated from each other if compared with the usual size and structure (**paucirradiate** scales); *C. rothschildi* has several scales of this type on pronotum (**Fig**. 13C); dorsal scales of *C. calvum* have also speciated rays but they are more uniform (**Fig**. 13B). In *Acrotelsa collaris*, a lobulate type of scales has been detected (indicated for the first time in this work, **Fig**. S5).

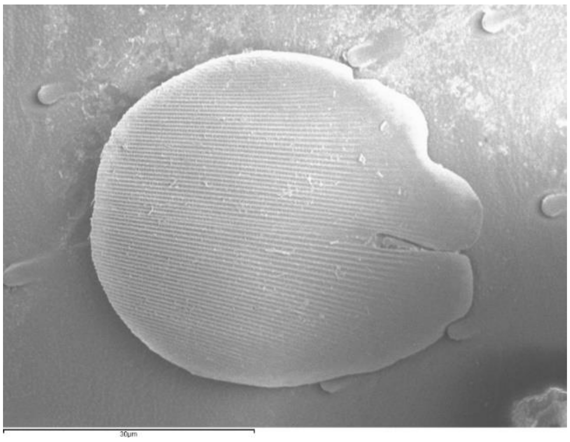

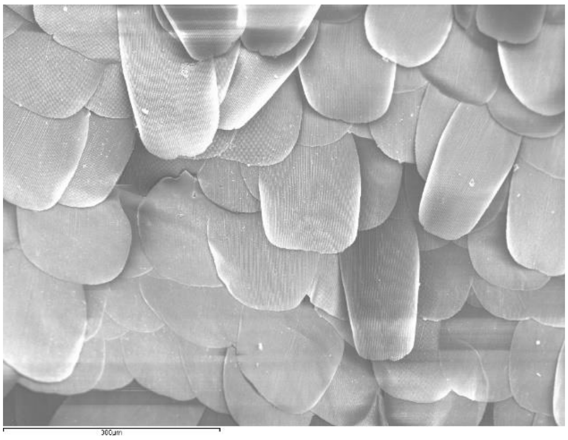

Fig. **S3** (Left). Usual rounded orbicular scale with dense rays. *C. targionianum* (Venezuela). **Fig**. **S4** (Right). Ventral scales of *Acrotelsa collaris* (Cape Verde), relatively uniform in size and density of rays and with shapes that range from rounded to subrectangular.

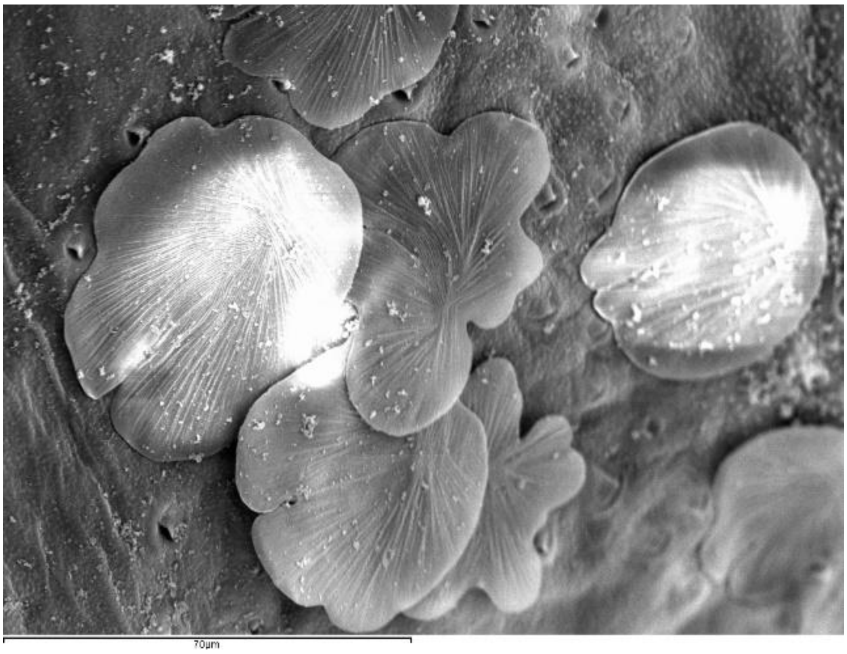

Fig. S5. Lobulate scales seen dorsally on head and pronotum of *Acrotelsa collaris* (Cape Verde).

4) Types and distribution of scales covering appendages

This character had rarely been used before Molero-Baltanás et al. (2010, 2012), who detected that there is a variability in the shape and distribution of scales on legs and other appendages in species of *Ctenolepisma*. The usefulness of these traits was confirmed by Smith et al. (2021) for some Australian species of the genus *Visma*, and in descriptions of species of other Australian genera and subfamilies. The problem with this character is that scales on appendages are small, usually hyaline, and most visible in ventral view and with light microscopy or SEM, but usually not detectable with stereomicroscope with usual magnification (up to x40); moreover, most of these scales are lost if specimens are not fresh enough. Although the variability observed is impressive, this character is constant for each species or group of species. Some taxa, as *Acrotelsa*, have all their appendages except the flagellum of antennae and, apparently, their terminal filaments, covered with rounded scales similar to those of the body but smaller (**Figs**. 4 and S6). But other taxa have most appendages and articles devoid of scales, only with setae, or with

scales that have modified shapes (subtriangular truncate, subrectangular, lanceolate, etc.). For example, some species only have scales on scapus of the antenna and on coxae, lacking scales in the remaining articles of legs (**Fig.** S7), such as *L. saccharinum*, while other species such as *Ctenolepisma (Sceletolepisma) villosum* and *C. ciliatum* have additional rounded scales on femora (**Fig**. 8B and also **Figs**. S8 and S9 in this document). Differences in the scale cover between the outer and the inner sides of some articles of the legs are frequent (compare **Fig**. S8 and S9); see also Molero et al. (2010). Other species have rounded scales on coxae and scapus but lanceolate scales on tibiae, femora, styli and cerci (*C. lineatum*, **Figs**. 8D, 15B and S10), or truncate scales on femora (*C. nicoletii*, see Molero et al. 2012), etc.

In non-synanthropic species it seems that this diversity of shapes and distribution of scales on appendages is even higher, and probably useful to distinguish between closely related species that previously were considered as the same species or cryptic species. Within Ctenolepismatinae, the subfamily that includes most synanthropic species, the same type and distribution of scales has been detected in *C. ciliatum*, *C. longicaudatum*, *C. villosum*, *T. domestica* and *T. aegyptiaca*, can be used to distinguish one or two clades including these five species (together with some other free-living in Palaearctic region) from another clade including *C. lineatum* and *C. nicoletii*, and another one including *C. calvum* and *C. rothschildi*. Most of these clades are congruent with molecular results given in this work.

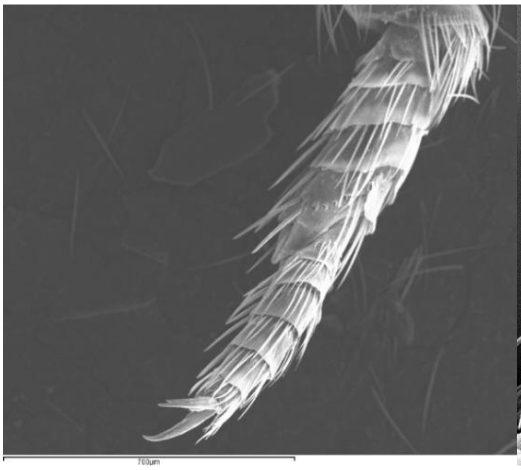

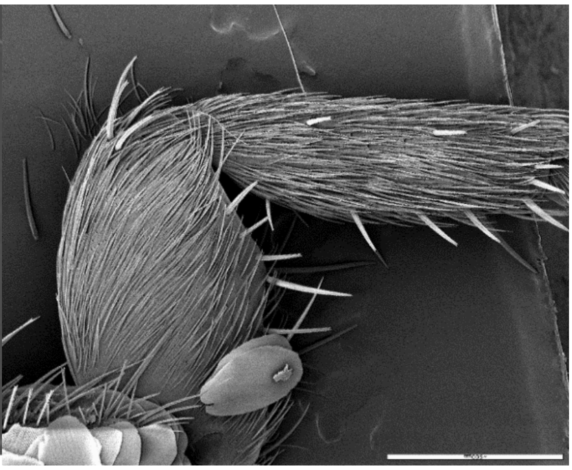

Fig. S6 (Left). *Acrotelsa collaris*. (Cape Verde), tarsus of one leg covered with scales and some setae. The remaining articles of the legs are covered with similar scales. **Fig. S7** (Right). *Allacrotelsa kraepelini* (Spain), apex of coxa covered with scales (one of them detached), and femur and tibiae lacking scales, only covered by setae and some macrochaetae. *Lepisma saccharinum* has a similar pattern.

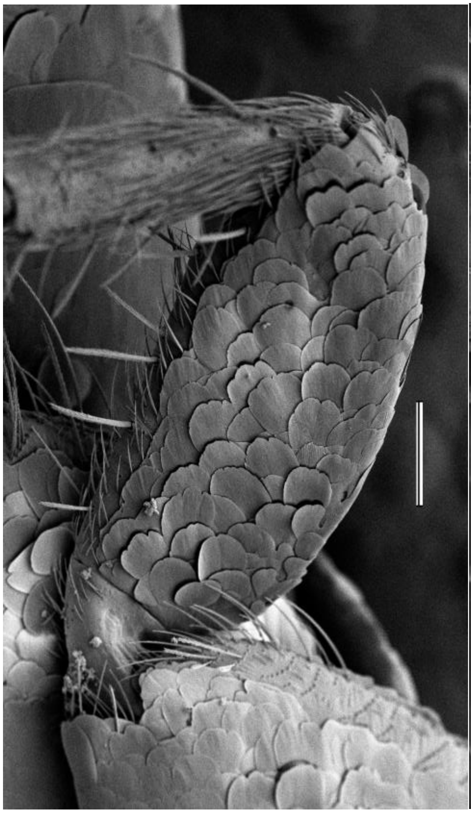 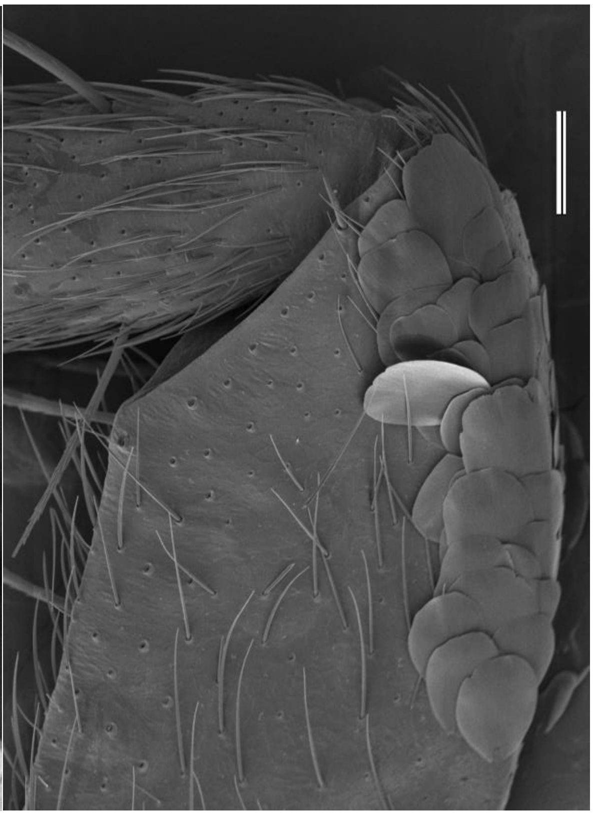

Fig. S8 (Left). *Thermobia domestica*, inner side of the leg (visible in ventral view of the insect) showing the femur completely covered by rounded scales with similar shape to those of coxa (in the bottom part of the SEM photograph). Scale: 0.2 mm. **Fig. S9** (Right). The same species, outer side of the leg (visible in dorsal view of the insect) showing the femur only covered with rounded scales on its anterior margin, on the right part of the SEM photograph. The remaining surface of the femur and the tibia (on the upper part of the photo) is covered only with setae. Scale: 0.1 mm.

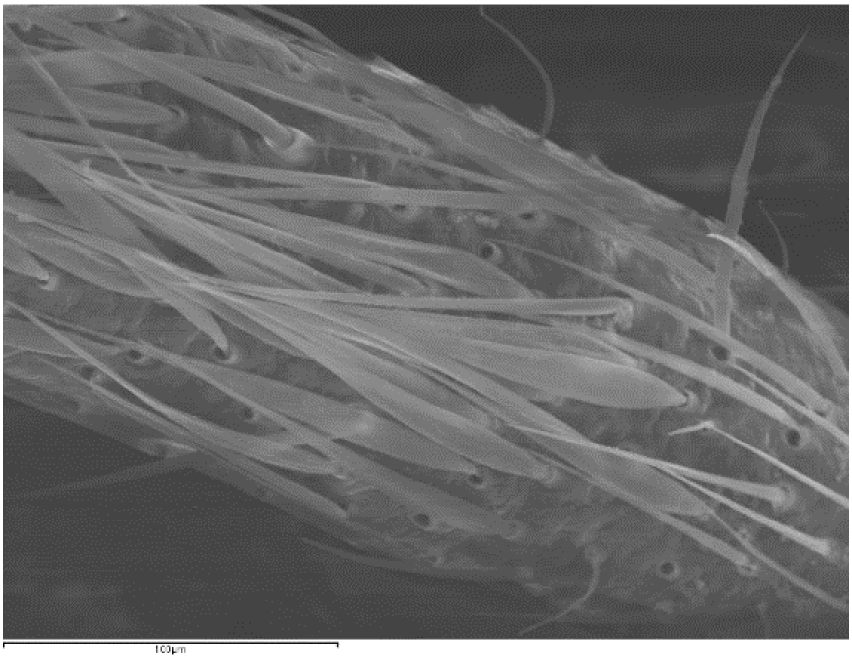

Fig. S10. *Ctenolepisma lineatum* (Spain), showing narrow and acutely pointed lanceolate scales on the stylus IX.

5) Cephalic chaetotaxy

Macrochaetae of frons of Ctenolepismatinae are usually arranged in tufts (or bushes) that can be smaller or larger, they even can be fused between together. The occurrence of tufts is variable on clypeus and labrum depending on the genus and species. In Lepismatinae, macrochaetae are not usually grouped in dense tufts, but more disperse. In *Ctenolepisma*, *Thermobia* and other genera of Ctenolepismatinae, a gap devoid of setae is characteristic in the median part of the frons (see **Fig**. 11B), while in *Lepisma* and other Lepismatinae (see **Fig**. 11A) the frons is continuously setose, without a gap in the middle, but usually less densely hairy because of not forming tufts.

6) Number and arrangement of papillae of the last article of the labial palp

This character has been mentioned as important for taxonomy of Zygentoma (Larink 1984), but some caution should be given because the number of papillae seems to be variable in some species, including the synanthropic *Ctenolepisma longicaudatum* and *C. villosum* (see morphological remarks of these species in the main text of the present work). The arrangement of papillae is more constant: they can be arranged on a single row, as in *Ctenolepisma* or *Thermobia*, or in two rows (3+2), as in *Lepisma saccharinum* or in *Acrotelsa collaris* (**Fig**. S11).

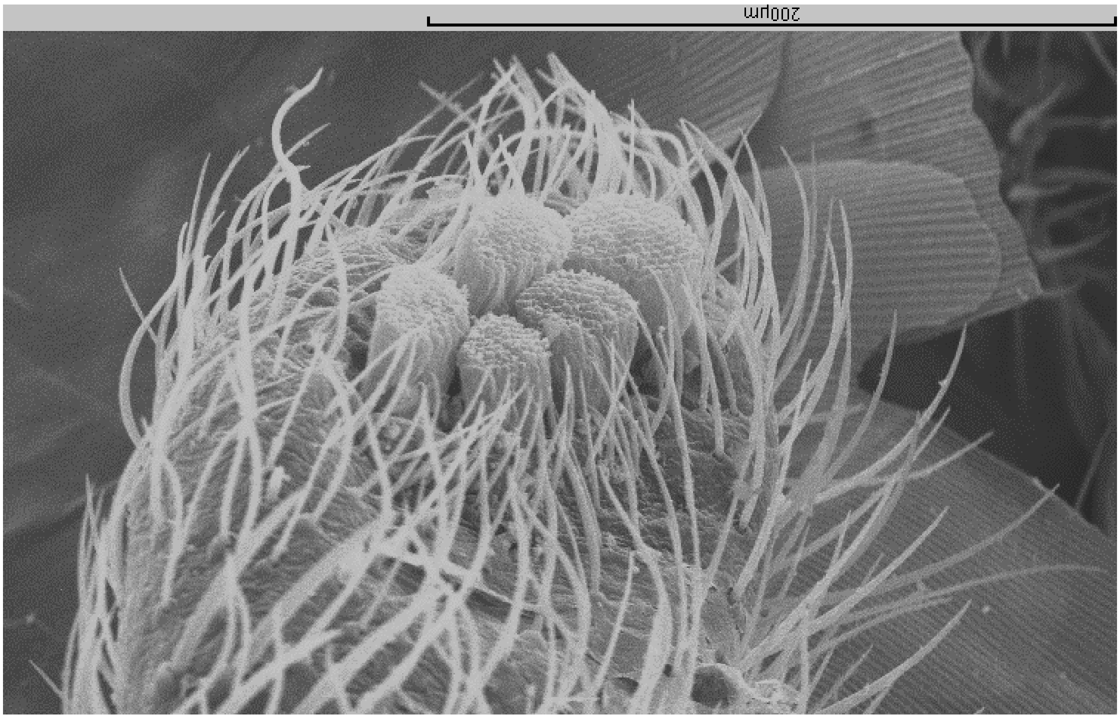

Fig. S11. *Acrotelsa collaris* (Cape Verde), last article of the labial palp with papillae arranged in two rows.

7) Chaetotaxy of the anterior margin of pronotum

The pronotum, i.e., the dorsal plate of the prothorax, can be devoid of macrochaetae along its anterior margin, as in *Lepisma saccharinum*, or can have a **pronotal collar**, i.e., a transverse field of macrochaetae that consists of one to several rows of macrochaetae irregularly arranged connecting the anterolateral corners of the pronotum and placed just behind the occiput (**Fig**. S12). In *Ctenolepisma* and *Thermobia*, this collar is reduced in

its lateral extremes to a single row of macrochaetae, the anterolateral row, that can consist of feathered (in *C. lineatum* and related species) or smooth bifid macrochaetae (as in *C. longicaudatum* and related species such as *C. ciliatum* or *C. iranicum*; see **Fig**. S13).

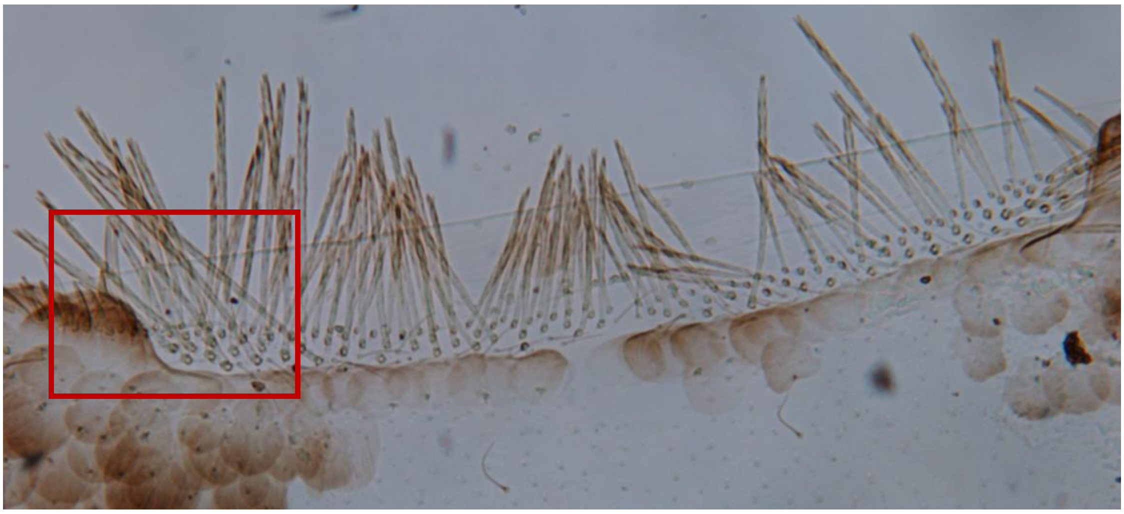

Fig. S12. Anterior margin of the pronotum of *Ctenolepisma iranicum* showing the macrochaetae of the collar. Some short setae of the anterolateral row are visible on the left part of the red square.

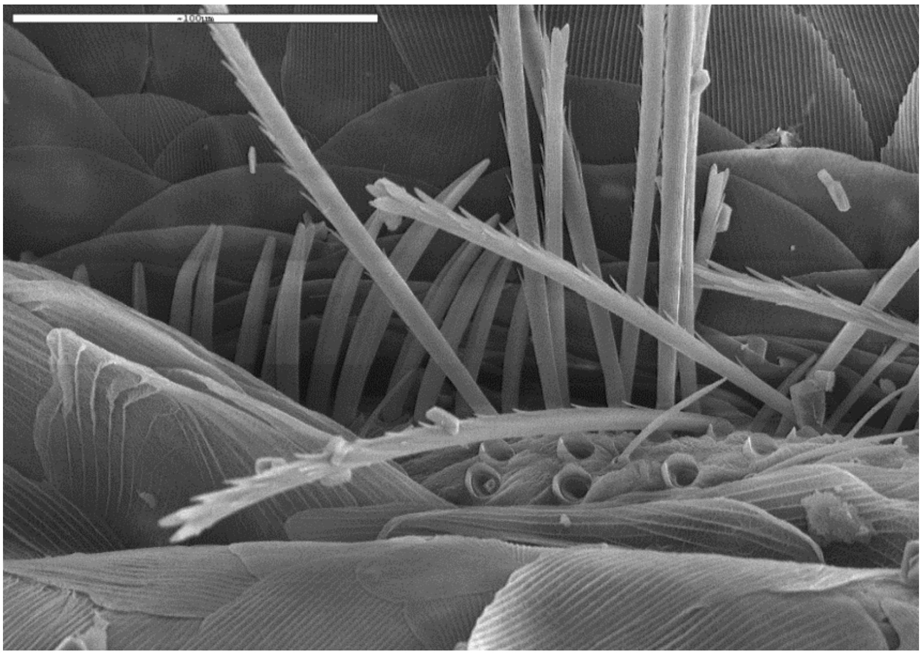

Fig. S13. Detail of the pronotal collar of *Ctenolepisma ciliatum* showing some insertions and feathered macrochaetae, and the anterolateral row (on the left) with smooth macrochaetae. The area visible in this photo correspond with the red square marked in the previous photograph. This character and the one presented in Fig. S10 are similar in *C. longicaudatum*, *C. calvum*, *C. rothschildi*, *C. villosum* and other species of the genus with trapezoidal tenth urotergite.

A third type of chaetotaxy corresponds to *Acrotelsa* and some other related genera of the subfamily Acrotelsatinae, that have only 1+1 groups of macrochaetae (anterolateral tufts) but the middle part of the anterior margin of the pronotum is devoid of setae.

8) Chaetotaxy of the lateral and posterior margins of thoracic nota

On lateral margins of thoracic plates, setae and macrochaetae can be isolated or arranged in combs. The first option is observable in *Lepisma saccharinum*, and lateral combs of macrochaetae can be observed in Ctenolepismatinae (**Fig**. S14). In posterior margins of nota, most Ctenolepismatinae can bear 1+1 posterolateral bristle-combs inserted more or less close to the posterolateral corners of the plate, but in some taxa these combs can be reduced to one or few macrochaetae. This is the case of *Ctenolepisma calvum*, where it was described that has only 1+1 isolated macrochaetae, but we have checked several specimens and some of them have small combs of 2-3 macrochaetae. Thoracic nota of *Lepisma* and *Acrotelsa* lack setae on their posterior margin or have some small setulae that can be detected only with high magnification.

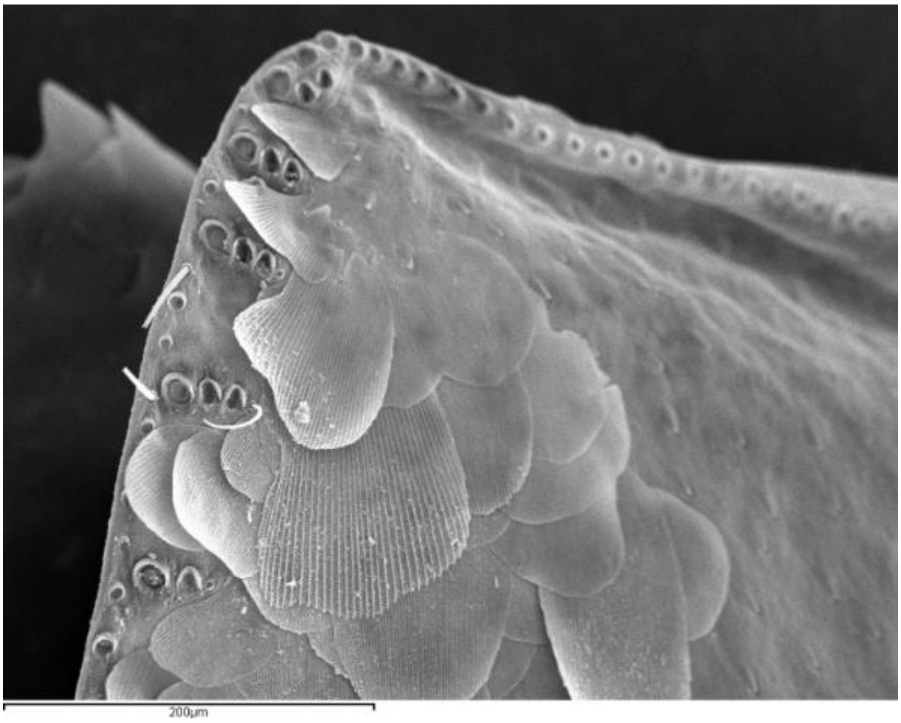

Fig. S14. *C. targionianum* (from Venezuela). Anterior part of the lateral margin of the pronotum with several combs consisting of only 2-3 macrochaetae each (only the insertions of macrochaetae are visible). The insertions of the anterolateral row of the anterior margin are also visible in the right upper part of the photo.

9) Type and distribution of trichobothrial areas

Trichobothria or bothriotricha are long and straight fine hairs common in terrestrial arthropods as mechanoreceptors. They are inserted in areas of the tegument named as trichobothrial areas (hereinafter in this section, T-areas), where they are usually accompanied by other types of sensilla (trichodea, and chaetic sensilla). Mendes (1986) studied the distribution of T-areas on the disc of the nota and distinguished two main types of these areas: open (in contact with the lateral margin of the notum) and closed (surrounded by scales that separate them from the lateral and posterior margins of the notum). The usual number of T-areas in a notum is 4 (2 anterior and 2 posterior). In *Lepisma*, the anterior areas are open, and the posterior ones are closed (**Fig**. S15). But in *Ctenolepisma*, *Thermobia* and most Ctenolepismatinae all of them are open, associated with one lateral comb of macrochaetae (**Fig**. S16).

Recently, the first author and G. Smith from Australia have observed that the more anterior of posterior position of T-areas in the lateral margins is constant for each species and similar in related species. This position in Ctenolepismatinae can be referred to the lateral comb where the trichobothria are associated (**Fig**. S17). If we give the name N to the last lateral comb of a notum, N-1 to the penultimate, N-2 to the antepenultimate, and so on, the posterior T-areas are usually associated with the last comb (N), but the anterior T-areas can be inserted in different positions. For example, we can distinguish between species of *Ctenolepisma* that have the anterior T-area of the pronotum associated with the N-2 combs, such as *C. lineatum* or *C. nicoletii*, while other species have this anterior T-area associated with the N-3 combs (anterior to antepenultimate), such as *C. ciliatum* or *C. longicaudatum*. In the first two species, the anterior T-areas of the mesonotum are associated with the N-1 combs, but in the last two species, the anterior T-areas of the mesonotum are associated with the N-2 combs (see **Fig**. S17).

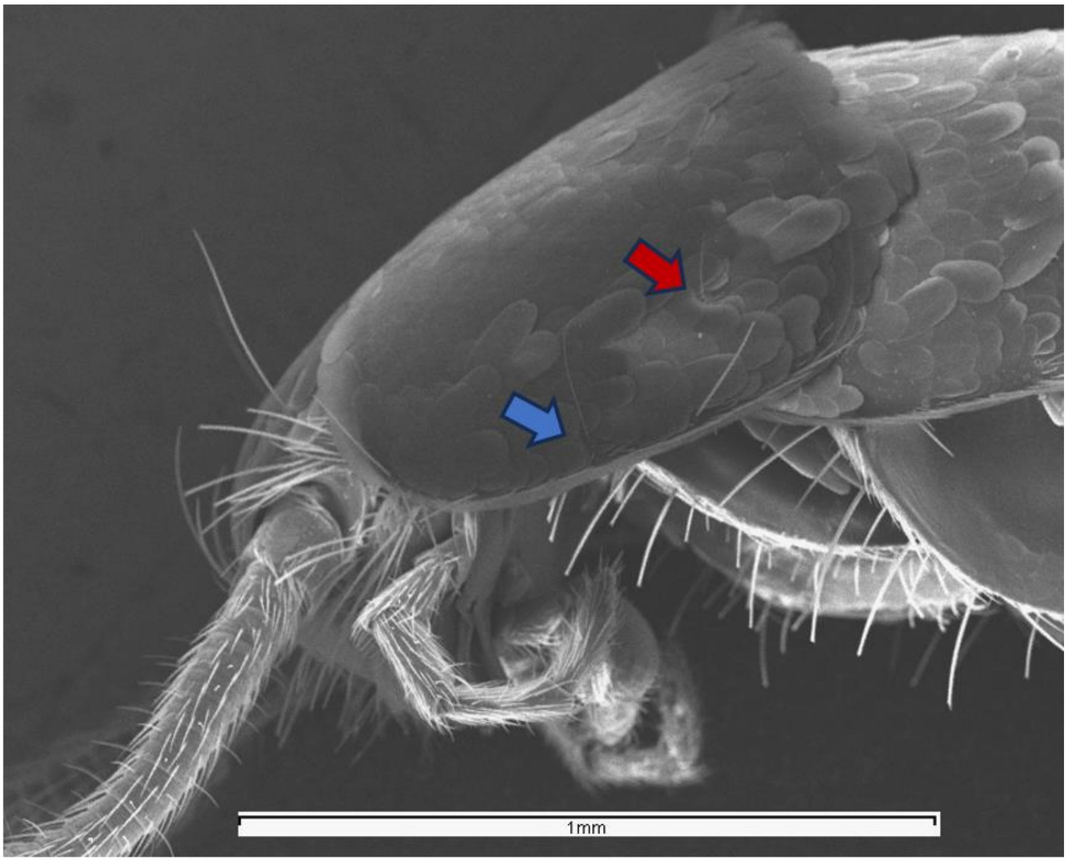

Fig. S15. SEM photograph showing the prothorax of *Lepisma saccharinum* in lateral view. In the pronotum, the anterior trichobothrial area is open, in contact with the lateral margin of the sclerite (blue arrow) and the posterior trichobothrial area is closed, completely surrounded by scales (red arrow). The trichobothrial setae are visible on each of both areas.

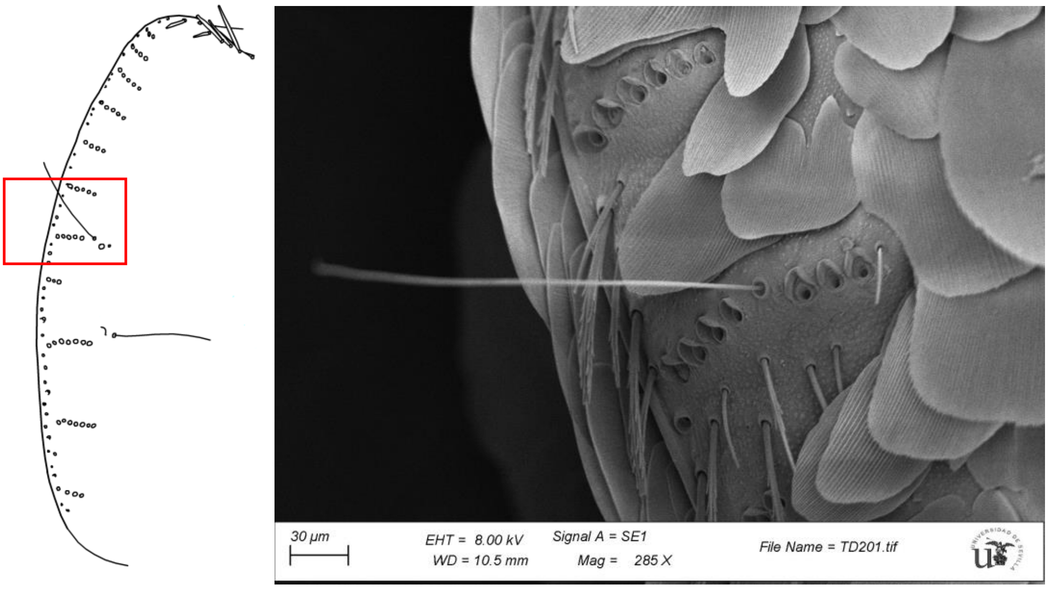

Fig. S16. Lateral side of the pronotum of *Thermobia domestica*. The drawing on the left shows the unique position of trichobothria in this genus, documented for the first time in this work. The anterior trichobothrium is on the N-4 comb, and the posterior one on the N-2 (antepenultimate lateral comb). An explanation of the terminology used for these positions is explained in the text and in Fig. S17. An area similar to that surrounded by the red square is presented in the SEM photograph on the right. The position of the anterior trichobothrium is clearly seen associated with one comb (the posterior of the two shown in the photograph). Most macrochaetae are detached. When the trichobothrial setae has come off, it is difficult to identify its position with light microscopy, but with SEM it is clearly visible that the insertion has a different shape and structure than other types of setae.

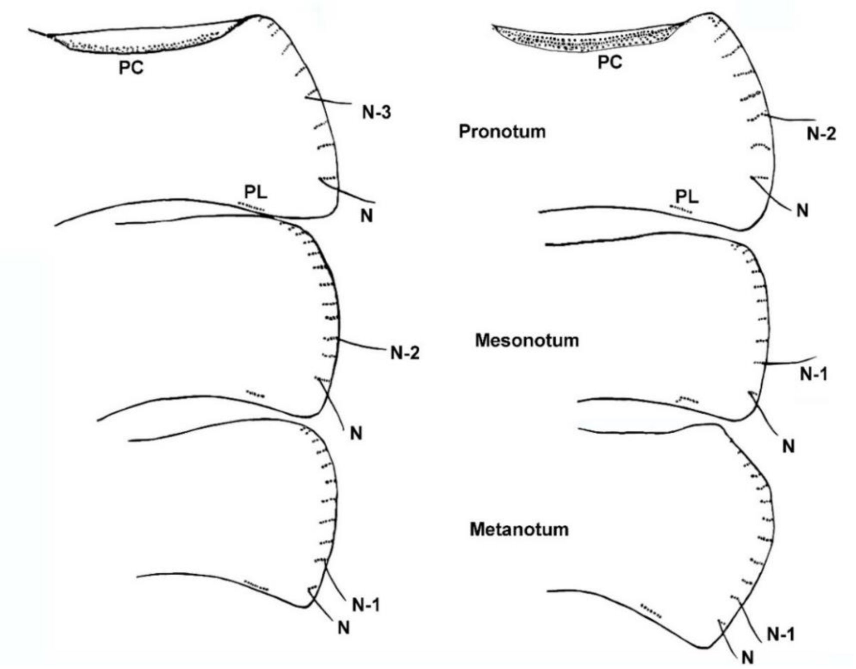

Fig. S17. Arrangement of trichobothrial areas on lateral combs of thoracic nota of two species of *Ctenolepisma*: *C. longicaudatum* (left) and *C. lineatum* (right). PC: Pronotal collar. PL: Posterolateral comb of pronotum (a similar comb is visible on the same position of mesonotum and metanotum). N: trichobothrium associated with the last lateral comb. N-1: trichobothrium associated with the penultimate lateral comb. N-2: trichobothrium associated with the antepenultimate lateral comb. N-3: trichobothrium associated with the lateral comb placed before the antepenultimate one.

The thin and long setae of T-areas often break off, so only their insertion can be detected, and this is difficult even with light microscopy observation. This makes it necessary to see several specimens or use fresh material where this situation is less frequent.

In some cases, combs of lateral margins of nota are reduced so it becomes difficult to describe the position of the anterior and posterior trichobothria of each side referring to lateral combs. In this case, it is better to follow the descriptive method given by Smith et al. (2021) where the position is presented as the ratio distance to the anterior margin/total length of the notum. The occurrence of T-areas in other parts of the body (head, legs) is not well studied yet, but probably deserves more attention in the future. On antennae, trichobothrial setae are useful to detect sections of the flagellum that are repetitive in its pattern of sensilla (Molero-Baltanás et al. 2000).

10) Thoracic sternites: shape and chaetotaxy

In most Lepismatidae, sternal plates are developed and posteriorly free, attached to the ventral tegument only by their anterior margin, covering the bases of coxae (**Fig**. 3C, and S24 after the glossary), but in representatives of the family Acrotelsatinae, thoracic sternites are not free and coxae cover the sterna; see Fig. 190 in Smith (2016), where this character is discussed with more detail. Thoracic sternites (probably better considered as a group of characters) have not been described for several common silverfish, but it has been proved its utility for distinguishing species. The relative size of these sternites is a diagnostic character; in some Lepismatidae, the prosternum is smaller and with a different shape than meso- and metasternum, while in other taxa the difference in size and shape between these sternites is less pronounced. The ratio length/width (L/W) of each sternite is an interesting metric. The shape of the posterior margin and the chaetotaxy are variable characters, and even intraspecific variability can be detected; but after examining several individuals, the constant traits for each taxon can be easily identified (macrochaetae arranged in combs or not along posterolateral margin, number of combs, number of rows of macrochaetae of each comb, etc).

For example, this character is very diagnostic for distinguishing *Ctenolepisma lineatum* from free-living species that are very similar and live in South Europe (see Molero Baltanás et al. 2012; Molero-Baltanás et al. 2005) and probably in SW North America. In this work details are provided for several species where the thoracic sternites had never been previously described. The thoracic sternites of *Ctenolepisma calvum* and *Ctenolepisma* (*Sceletolepisma*) *villosum* are presented in **Figs**. 21D-F and in **Figs**. 30B-D, respectively. Those of *Ctenolepisma longicaudatum* are shown in **Fig**. S18. Differences in shape and in number of combs of macrochaetae can be checked when comparing their respective drawings. Those of *Thermobia domestica* and *Thermobia aegyptiaca* are presented in **Fig**. **S19** in this document.

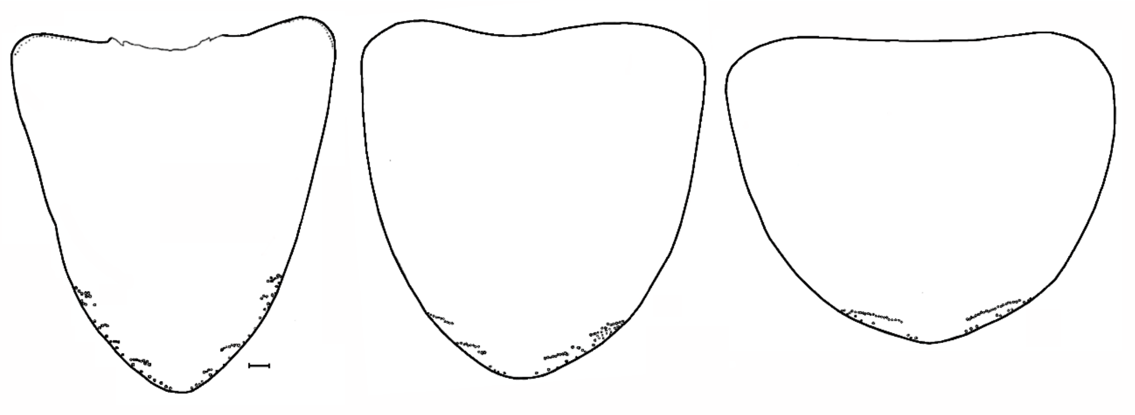

Fig S18. Thoracic sternites of *Ctenolepisma longicaudatum*. Prosternum (left), mesosternum (middle) and metasternum (right). Scale: 0.1 mm. The prosternum has usually 3-4 pairs of combs in its distal half. The mesosternum usually shows 2-3 pairs of combs and the metasternum only 1+1 (sometimes each comb is divided into two very close ones). In the metasternum, the distance between the subapical combs is usually shorter than the width of a comb. Compare with Figures 19D-F and 24B-D in the main text for other species of *Ctenolepisma,* and with Fig. S19 that presents some thoracic sternites of *Thermobia* species.

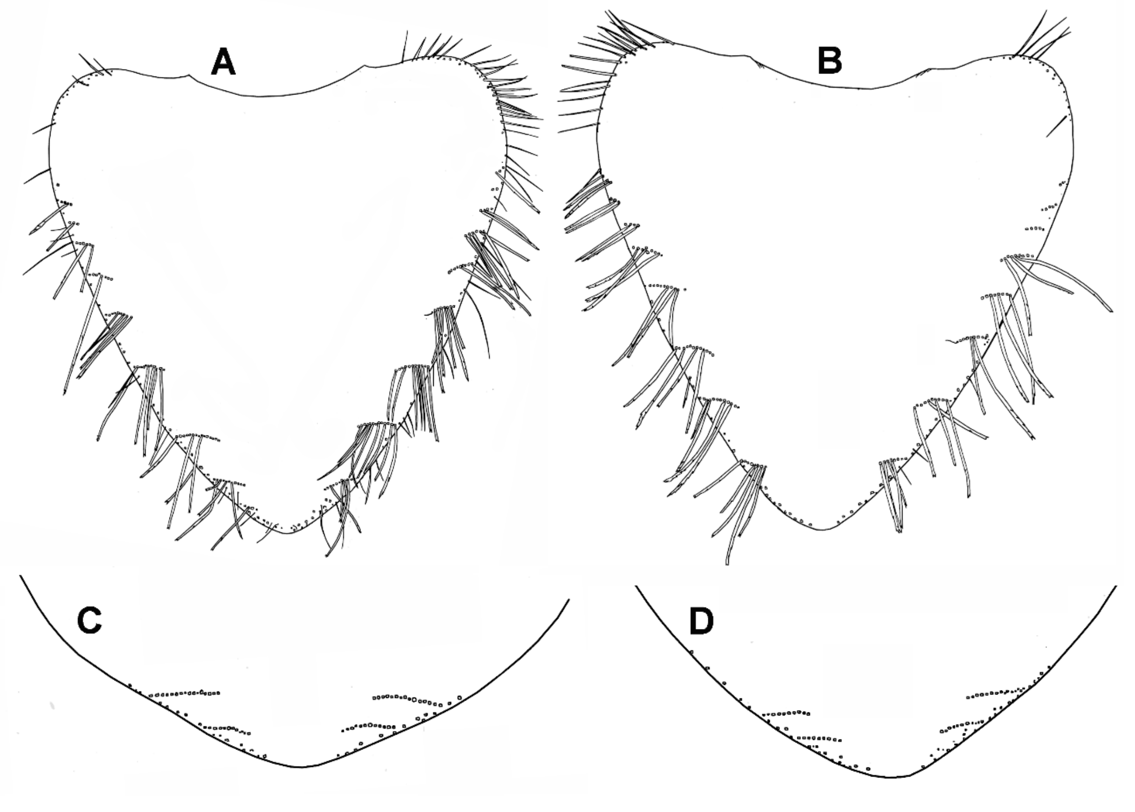

Fig. S19. Thoracic sternites of *Thermobia* species (all drawings from specimens collected in Iran and similar to those from Egypt). **A**: Prosternum of *T. aegyptiaca*. **B**. Prosternum of *T. domestica*. **C**. Posterior margin with subapical combs of the metasternum of *T. aegyptiaca*. **D.** Posterior margin of *T. domestica*. When macrochaetae are come off, only represented by their insertions.

11) Chaetotaxy of urotergites

This is one of the characters that has been used more frequently for the taxonomy of Lepismatidae for diagnosis of genera and species. Most of the previously published identification keys of silverfish use the arrangement of macrochaetae as the main (and frequently, as the unique) character for distinguishing species. The macrochaetae of the posterior margin of abdominal tergites in a high number of species are grouped in combs (**Fig**. 10B); the highest number of combs is 3+3, i.e., bearing at each side one comb in submedian position, one in sublateral position and one in lateral position (**Fig**. 12).

Some of the combs on these three positions can be absent, and only 1+1 or 2+2 combs can be visible (sometimes all combs are absent, as for example in the urotergite IX of several genera of Ctenolepismatinae). This arrangement in combs is a high proportion of genera of Lepismatidae, although some different states of the character are present in several specialized genera, such as in some apomorphic psammophilous genera of Namib desert. *Thermobia* has a maximum number of 2+2 combs on each abdominal tergite while some urotergites of *Ctenolepisma* (at least II-V) bear 3+3 combs of macrochaetae.

In other species (for example, in most Lepismatinae) the number of macrochaetae of each comb is reduced to only one, and there are only 1+1 isolated macrochaetae of submedian and sublateral positions, and the lateral comb is split into 1+1 isolated lateral macrochaetae and 1+1 small groups named as infralateral groups (this denomination is due to the fact that the lateral parts of each dorsal plate of the abdomen fold to embrace the sides of the body of the silverfish); see Mendes (1991), drawings in Robla et al. (2023) and **Fig**. 10A. This is the most common arrangement in Lepismatinae, including the genus *Lepisma*. Nevertheless, this character has to be assessed carefully, because macrochaetae can be very hyaline or detached and thus hard to see in macrophotographs or stereomicroscope, so microscopic examination is necessary to confirm their presence, frequently detecting their insertions. Moreover, some intraspecific variability has been detected in several taxa, suggesting that the number of combs or isolated macrochaetae on one specific segment should not be the only diagnostic character for defining a species (Molero et al. 2010) and that this character can be different in closely related species (Irish 1987), not being appropriate to establish groups with a phylogenetic sense, as Kaplin (1993) proposed.

12) Shape and chaetotaxy of the last abdominal tergite (urotergite X). Fig. 2.

This character has been frequently used in Lepismatidae taxonomy. In fact, it is very useful for distinguishing groups of species (mainly at generic level) but not so relevant to distinguish very closely related species. This abdominal tergite has a basal part whose width is similar to that of the preceding tergite, and a more or less big median expansion directed towards the rear. The shape of this expansion, usually referred as the shape of the abdominal tergite, ranges from trapezoidal with straight or slightly convex or concave hind margin (**Fig**. 2B, 2C) to triangular with acute to rounded posterior apex (**Fig**. 2A, 2D). The median expansion could be long (**Fig**. 2A, 2C) or short (**Fig**. 2B, 2D) and its ratio length/width has been used to describe species and as one character to distinguish species (Mendes 1982), although it should be combined with other characters; for this measure, the basal part of the tergite that has a similar width than the ninth urotergite is not considered. In *Acrotelsa*, the urotergite X is large, triangular, and posteriorly acute

(**Fig**. 2A). In *Ctenolepisma* of the subgenus *Sceletolepisma* (sensu Irish), which is going to be separated as an independent genus, and some species of the subgenus *Ctenolepisma s.str*., this posterior plate of the body is trapezoidal, with the hind margin straight or slightly concave or convex (**Fig**. 2B). In *Ctenolepisma lineatum*, *Thermobia domestica* and other related species, the shape is subtriangular, the tergite is very short and the posterior apex is more or less rounded or subacute (**Fig**. 2D).

Most genera of Ctenolepismatinae have one comb of macrochaetae in each posterolateral corner of this tergite, but some of the genera with large acutely triangular shape show several pairs of combs on their lateral sides (for example, in *Acrotelsa*) and some other genera have only marginal setae but not clearly visible combs, as happens in *Lepisma*. Some intraspecific variability of the shape has been detected that should be considered for diagnosis of species; for example, Mendes (1993) identified this variability in North African *Ctenolepisma* previously identified as *C. lineatum* that could probably correspond to a different species (very likely, to *C. brauni*).

13) Urosternal chaetotaxy

The arrangement of macrochaetae in the ventral side of the abdomen is generally used in combination with that of the dorsal side for diagnosis of genera and species of silverfish. Most urosternites of Lepismatidae usually have 1+1 lateral combs of macrochaetae on their posterior margin, and some genera or related group of species show one additional comb in median position of this margin. The median comb has been used by Irish (1987) to distinguish the subgenus *Sceletolepisma*, which represents probably a natural group inside the subfamily Ctenolepismatinae, and to establish several groups of species that perhaps represent different lineages in South African fauna, but this is not so clear if all the species of the subgenus are considered. As commented for urotergal chaetotaxy, these characters should be considered carefully because its possible intraspecific variability should be checked in some taxa. Additionally, some non-specialist entomologists have not examined it carefully compared with dorsal chaetotaxy (in live specimen, it is easier to observe insects in dorsal view) and some mistakes have been published. For example, Querner et al. (2022) mentioned that *Lepisma saccharinum* lacks abdominal combs, which is an error (See **Figs**. S20 and S21).

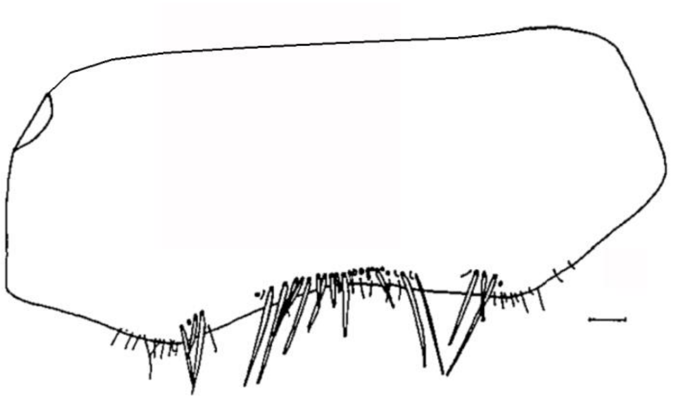

Fig. S20. Urosternite V of *Lepisma saccharinum*, with one median comb and two small lateral combs of macrochaetae on its hind margin. Compare with Fig. S.21. Scale: 0.1 mm.

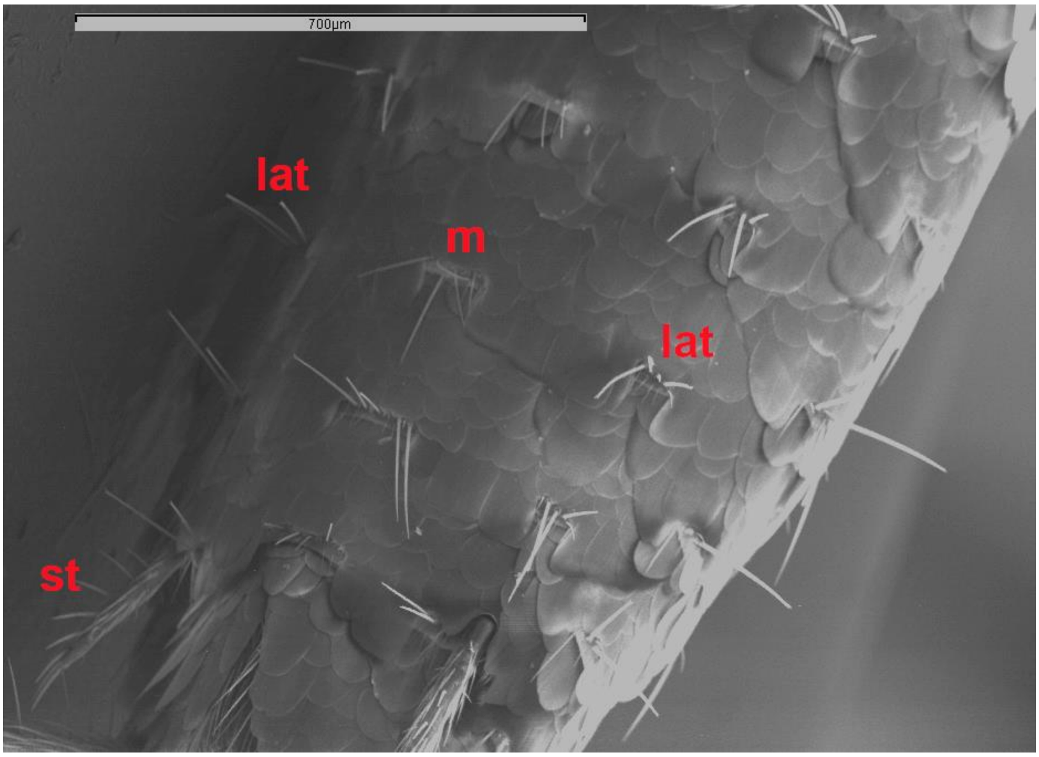

Fig. S21. SEM photograph of the ventral side of the abdomen of *Lepisma saccharinum*, showing the lateral and median combs of some urosternites. The median comb (m) and the lateral combs of macrochaetae (lat) of the urosternites VI are marked, as well as the stylus of the right side on the urosternite VIII.

14) Number of pairs of abdominal styli

Abdominal styli are appendages inserted at both sides of the posterior margin of some abdominal sternites. They are usually present on the ninth ventral sternite, that is divided in both sexes into two lateral coxites (**Fig**. 14A). The eight sternite, also divided into coxites in females (**Fig**. 14B), often bears styli. The presence of one pair of styli in the seventh and preceding sternites is frequent in other families of Zygentoma (for example, in most Nicoletiidae), but less frequent in Lepismatidae. The number of pairs of these appendages is characteristic of each species, but its number increases during postembryonic development, and the definitive number should be examined only in adults. But the problem in ametabolous insects is determining which is the minimum size to consider a specimen as adult if studies of the reproductive system have not been carried out. For example, in *Ctenolepisma lineatum*, the third pair of styli is not developed until the insect reaches about 8 mm of length. So, this character is not appropriate to identify young specimens. Moreover, the number can be different in both sexes of the same species. For example, in *Ctenolepisma villosum*, males bear one pair of styli and females bear two pairs.

15) Paramera

The presence or absence of paramera in males of Lepismatidae has a suprageneric relevance in their taxonomy. They are absent in several genera, for example in those belonging to the subfamily Ctenolepismatinae. When present, their shape and size, and some other traits can also be used for distinguishing genera. The genus *Lepisma*, for example, bears hyperdeveloped paramera with a large glandular area, reaching or surpassing the apex of the inner process of the ninth coxite (**Fig**. 9), while *Ctenolepisma*

and *Thermobia* lack paramera and males of *Acrotelsa* have small paramera without a clearly visible glandular area that do not surpass the inner process of the ninth coxite.

16) Type of ovipositor and number of divisions

The ovipositor results from the association of long appendages of the eighth and ninth segments, called **gonapophyses**, that are fused to form a long cylindric structure. In Zygentoma two main types of ovipositors have been distinguished (Mendes 1988b). Primary type ovipositors are thin and long, with small and thin specialized setae at their apical parts (**Figs**. 14B, 23E, 31G, S22). Secondary type ovipositors are stout and more or less sclerotized, bearing at their apex some fossorial spines. Since the structure of these spines and the degree of sclerotization varies in genera sharing this type of ovipositor, it seems that this is a homoplastic character inside Lepismatidae. Within synanthropic silverfish, the only species bearing this specialized type of ovipositor is *Acrotelsa collaris* (**Fig**. S23). The length of the ovipositor should be measured only in adult specimens, as well as the length of the ovipositor that surpasses the apex of the ninth styli or the apex of the ninth coxites relative to the length of these appendages. The number of divisions is also characteristic of each species, but sometimes hardly visible even in specimens mounted for microscopic examination. Nevertheless, these characters seem to show some intraspecific variability that should be assessed before considering these as differential characters between related species.

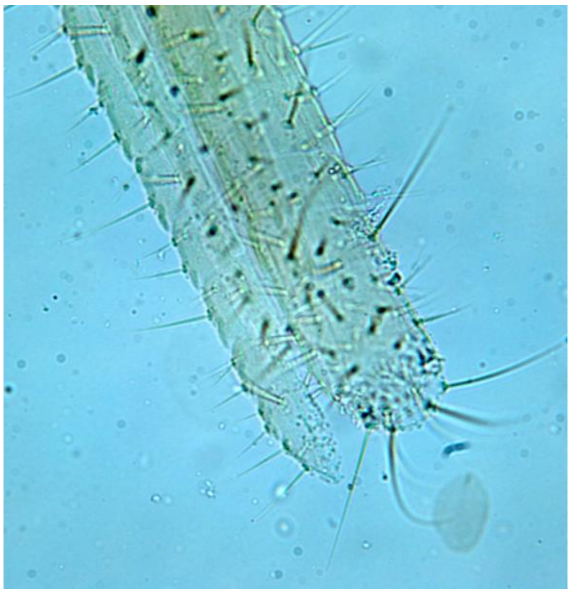
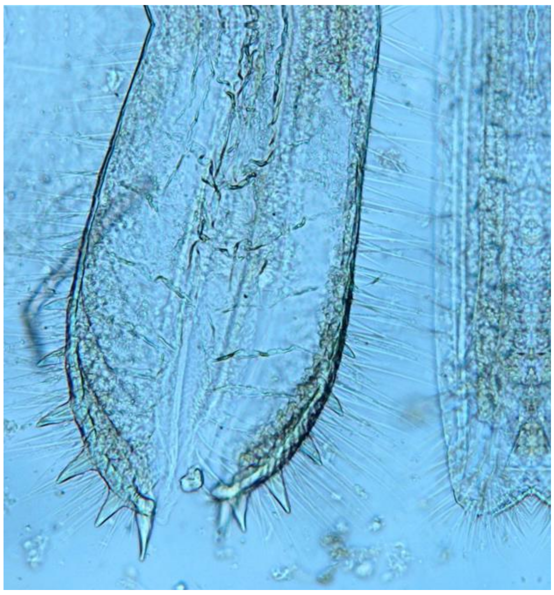

Fig. S22 (Left). Ovipositor of *Ctenolepisma lineatum*, of primary type. **Fig. S23** (Right). Ovipositor of *Acrotelsa collaris*, of secondary type, showing apical sclerotized teeth on the gonapophyses IX (gonapophyses VIII, on the right of the micrograph, lack spines).

Glossary

Some of the most common terms used in Zygentoma morphology and taxonomy (**Fig. S24**). Alphabetic list.

Bristle-comb. See macrochaetae.

Bush. See macrochaetae.

Chaetotaxy. Entomologists use this word considering that it is more precise than "setation" for referring to the arrangement and nomenclature of the different types of setae in the tegument of an insect.

Comb. See macrochaetae.

Coxites. A plate resulting of the division of the ventral plates (urosternites) in two plates at both sides of the penis (in males) or at both sides of the ovipositor (in females). In males, only the last sternite (ninth) is divided, resulting in two coxites (Coxites IX). In females, the two last urosternites are divided, resulting in coxites VIII and coxites IX. Frequently, an abdominal stylus is inserted in the posterior margin of each coxite, delimiting an inner process and an outer process. See urosternites and see stylus. See also **Fig**. S24.

Macrochaetae or **macrosetae** (singular, **macrochaeta**, or **macroseta**). Strong bristles that are bigger than the remaining setae of the body and function as mechanoreceptors (chaetic sensilla). They are present on the head (especially on frons and clypeus), usually on margins of thoracic sclerites, legs, terminal filaments and on the posterior margins of abdominal segments, dorsally and ventrally, but their arrangement is variable. They can be grouped in rows named **bristle-combs** (or, simply, combs of macrochaetae) or isolated. In the head they can be aggregated in dense groups (**tufts** or **bushes**) or not.

Mesonotum. Dorsal plate of the second thoracic segment.

Mesosternum. Ventral plate of the second thoracic segment.

Metanotum. Dorsal plate of the third thoracic segment.

Metasternum. Ventral plate of the third thoracic segment.

Notum (plural, nota). Dorsal plate in general. Frequently used for thoracic dorsal plates.

Paramere (plural, **parameres** or **paramera**). Paramera are bag-shaped organs that can be present ventrally in the ninth abdominal segment of males of most Zygentoma, at both sides of the penis and found on the inner side of the ninth coxites.

Paucirradiate. Scale with few rays.

Pronotum. Dorsal plate of the first thoracic segment.

Prosternum. Ventral plate of the first thoracic segment.

Urosternites. Ventral plates of the abdomen. In Lepismatidae, nine of these plates are visible. In males, the last one is divided in two lateral plates (coxites) at both sides of the penis. In females, the two last urosternites are divided in two coxites at both sides of the ovipositor. See also coxite.

Urotergites. Dorsal plates of the abdomen. In Zygentoma, there are ten segments, and their dorsal plates are numbered from urotergite I to urotergite X.

Stylus (plural, **styli**). Abdominal appendage, slender and fusiform, ending in one or several spines, present in the posterior margin of some abdominal sternites, usually the last ones. The term stylet is not appropriate since it refers better to piercing mouthparts of certain insects.

Tuft. See macrochaetae.

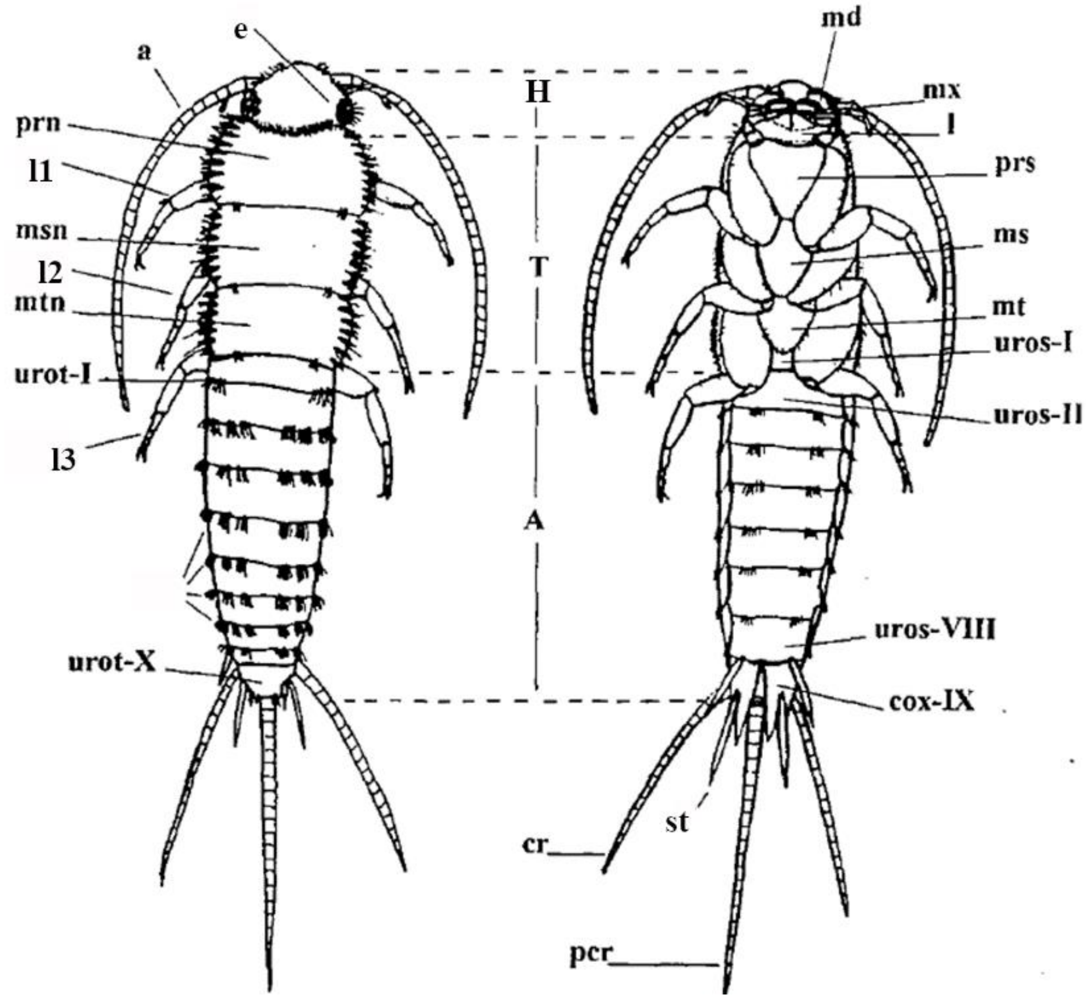

Fig. S24. Basic external anatomy of a Lepismatidae. Abbreviations: a: antenna; e: eye; prn: pronotum; l1: first leg; msn: mesonotum; mtn: metanotum; l2: second leg; mtn: metanotum; urot-I: urotergite I (dorsal plate of the first abdominal segment); urot-X: urotergite X (dorsal plate of the last abdominal segment); H: head; T: thorax; A: abdomen; md: mandible; mx: maxilla; l: labium; prs: prosternum; ms: mesosternum; mt: metasternum; uros-I: urosternite I (ventral plate of the first abdominal segment); uros-VIII: urosternite VIII (ventral plate of the eighth abdominal segment); cox-IX: coxite IX (left half of the divided abdominal plate of the ninth segment); st: abdominal stylus of the ninth segment; cr: cercus; pcr: paracercus (appendix dorsalis or median caudal filament).

References

Denis R. 1949. Traité de Zoologie: Anatomie, Systematique, Biologie. In: Grasse P-P. (Ed.). Super-Ordre des Ectotrophes. Paris: Masson. p. 209–275.

Irish J. 1987. Revision of the genus *Ctenolepisma* Escherich (Thysanura: Lepismatidae) in southern Africa. Cimbebasia (A). 7(11):147–207.

Irish J. 1990. Phylogeny of the Lepismatidae (Thysanura), with a revision of the southern African genera. Unpublished thesis. Faculty of Science, University of Pretoria.

Kaplin VG. 1993. On the systematics and phylogeny of the genera *Ctenolepisma* and *Sceletolepisma* stat. n. (Thysanura, Lepismatidae). Zoologicheskii Zhurnal. 72(7):34-51.

Larink O. 1984. The labial palp as a taxonomic characteristic in the Lepismatidae (Insecta: Zygentoma). Ann. Soc. Roy. Zool. Bel. 114:43–50.

Mendes LF. 1982. Notas e descrições de Lepismatidae afrotropicais (Zygentoma: Apterygota) II. Géneros *Ctenolepisma* e *Namunukulina*. Rev. Zool. Afric. (Bruxelles). 93(3):591–661.

Mendes LF. 1986. International Seminar on Apterygota. In: Dallai R. (Ed.). Sur quelques caracteristiques morphologiques des Lepismatidae (Zygentoma: Insecta). III. Les aires trichobothriales. Siena (Italy): University of Siena.

Mendes LF. 1988a. Actas III Congresso Iberico de Entomologia. In: Universidad de Granada (Ed.). Sobre algumas características morfológicas dos Lepismatidae (Zygentoma: Insecta). V. O tipo de macroquetas. Granada: Universidad de Granada. p. 143–148.

Mendes LF. 1988b. Actas III Congresso Iberico de Entomologia. In: Universidad de Granada (Ed.). Sobre algumas características morfológicas dos Lepismatidae (Zygentoma: Insecta). IV. Parámeros e ovopositor. Granada: Universidad de Granada. p. 133-142.

Mendes LF. 1991. Advances in management and conservation of soil fauna. In: Veeresh GK, Rajagopal D, Viraktamath CA. (Eds). On the phylogeny of the genera of Lepismatidae (Insecta: Zygentoma). New Delhi Bombay Calcutta: Oxford & IBH Publishing Co. Pvt. Ltd. p. 3–13.

Mendes LF. 1993. New data on the thysanurans (Microcoryphia and Zygentoma: Insecta) from Northern Africa and from the Near East. Garcia de Orta, Séries Zoologia, Lisboa. 18(1-2):79–93.

Mendes LF. 2018. Insect Biodiversity: Science and Society. Foottit RG, Adler PH (Eds.). Biodiversity of the Thysanurans (Microcoryphia and Zygentoma). Oxford (UK): John Wiley & Sons Ltd. pp. 155–198.

Molero-Baltanás R, Fanciulli PP, Frati F, Carapelli A, Gaju-Ricart M. 2000. New data on the Zygentoma (Insecta, Apterygota) from Italy. Pedobiologia. 44:320–332.

Molero-Baltanás R, Gaju-Ricart M, Bach de Roca C. 2005. *Ctenolepisma almeriensis* n.sp. of Lepismatidae (Insecta, Zygentoma) from south-eastern Spain. Anim. Biodiv. Conserv. 28(1):91–99.

Molero-Baltanás R, Gaju-Ricart M, Bach de Roca C, Mendes LF. 2010. On *Ctenolepisma ciliata* and a new related species, *Ctenolepisma armeniaca* sp.n. (Zygentoma, Lepismatidae). Deutsche Entomologische Zeitschrift. 57(2):243-252.

Molero-Baltanás R, Gaju-Ricart M, Bach de Roca C. 2015. Actualización del conocimiento del género *Ctenolepisma* Escherich, 1905 (Zygentoma, Lepismatidae) en España peninsular y Baleares. Boletín de la Asociación Española de Entomología. 39(3–4):365–390.

Molero Baltanás R, Gaju Ricart M, Bach de Roca C. 2012. New data for a revision of the genus *Ctenolepisma* (Zygentoma: Lepismatidae) redescription of *Ctenolepisma lineata* and new status for *Ctenolepisma nicoletii*. Ann. Soc. Entomol. Fr. 48(1–2):66–80.

Querner P, Szucsich N, Landsberger B, Erlacher S, Trebicki L, Grabowski M, Brimblecombe P. 2022. Identification and Spread of the Ghost Silverfish (*Ctenolepisma calvum*) among Museums and Homes in Europe. Insects. 13:855.

Robla J, Gaju-Ricart M, Molero-Baltanás. 2023. Assessing the Diversity of Ant-Associated Silverfish (Insecta: Zygentoma) in Mediterranean Countries: The Most Important Hotspot for Lepismatidae in Western Palaearctic. Diversity. 15(5):635.

Smith GB. 2016. Revision of the genus *Anisolepisma* (Zygentoma: Lepismatidae: Acrotelsatinae). Rec. Aust. Mus. 68(6):269–312.

Smith GB, Mitchell A, Molero-Baltanás R. 2021. Molecular and morphological studies identify a new genus within the Heterolepismatinae (Zygentoma: Lepismatidae). Zootaxa. 5030:1–118.
