# Supplementary material for: Worldwide revision of synanthropic silverfish (Insecta: Zygentoma: Lepismatidae) combining morphological and molecular data
Source: J Insect Sci. 2024 May 4;24(3):1. doi: 10.1093/jisesa/ieae045 (PMC11069193; doi:10.1093/jisesa/ieae045)
Supplement: ieae045_suppl_Supplementary_Materials_4 [file ieae045_suppl_supplementary_materials_4.pdf]

Supplementary Material 4. History of taxonomy and identification of synanthropic Lepismatidae: some errors and confusions (Past and recent)[1]

This supplementary document shows historical and recent examples of errors and confusions in the identification and taxonomy of several species of synanthropic Lepismatidae. Some of these errors simply show the historical difficulties in making appropriate diagnoses of such species when using inappropriate characters, but others perhaps reveal a lack of concern of researchers and/or editors for correct identification in different studies recently published. This document does not intend to present an exhaustive list of all the errors committed, but only a representative sample of some of the most relevant ones (A-G sections), highlighting that the problem is not only a historical issue but continues today and is expanding through several platforms. Although there are few available expert taxonomists to be consulted, some entomologists that are not aware of the current taxonomy and diversity of these insects made important mistakes. The low value placed on taxonomic research of non-key groups represents a threat to the future identification of many animal groups that are more diverse than is generally believed. 'Artificial intelligence' tools cannot be a substitute tool for taxonomic expertise, at least in animal groups that require a thorough microscopic examination for their sure identification, as happens with Zygentoma. After the work of Escherich (1905), the taxonomy of Lepismatidae was clarified and diagnosis of the most common species were accurate enough. Non-experts investigating silverfish could have consulted this or subsequent works by specialists such as F. Silvestri, J. Paclt, P. Wygodzinsky, L.F. Mendes, etc. to avoid identification mistakes. Most of these authors did not publish their work in English and this could lead to some native English-speaker authors to look for inappropriate works that were not made by authentic experts, or to dispensing with consulting any reference work. This is the only reason to justify inexplicable identification errors that have been spread even in articles published recently in indexed journals and in which, not only their authors, but also the editors and reviewers of these articles, are responsible for the obvious identification errors presented. This document finish with a table (section H) comparing the descriptions of *Ctenolepisma villosum* and *C. targionii* made by Yosii (1939) and Stach (1935) respectively, the first on the basis of Japanese specimens and the second on the basis of Egyptian insects (Table 1). A third description, made by the first author of the present work in his unpublished thesis (Molero Baltanás, 1995) on the basis of Spanish and Italian *C. targionii* (material published in by Molero-Baltanás *et al*. (1996; 2000; 2015), is presented in the same Table. Evident similarities are detected, with only subtle differences that can be interpreted as intraspecific variability, supporting the synonymy between *C. villosum* and *C. targionii*. For facilitating navigation across the document, enable the navigation panel in view.

[1]Supplementary document to the paper «**Worldwide revision of synanthropic silverfish (Insecta: Zygentoma, Lepismatidae) combining morphological and molecular data»** (2024).By Rafael Molero-Baltanás, Andrew Mitchell, Miquel Gaju-Ricart & Jairo Robla in *Journal of Insect Science.*

A. Mistakes with *Lepisma saccharinum* Linnaeus, 1758 in the XVIII and XIX centuries.

The two first mistakes during the history of silverfish appeared in the first description of the first species of Zygentoma established (Linnaeus, 1758). One consisted of attributing a feminine grammatical gender to the word *Lepisma*, but this word is of neuter gender (Molero-Baltanás et al. 2016). The second one was to assume that the geographic origin of this species is America. Linnaeus referred to the description of the book “Insects of Jamaica” by Brown (1756) named in page 425 as book-worm, so it was synanthropic in America (i.e., even in those years its occurrence in America could have been interpreted as not being native). Brown’s description could fit with any Lepismatidae. Linnaeus refers also to illustration byof George Adams in his book “Micrographia illustrata”. We have consulted the fourth edition of this book (Adams, 1771) where the figure 147 (Plate 31, besides page 84) shows a silverfish that could correspond to the authentic *Lepisma saccharinum* but has annulated antennae and caudal filaments with alternative dark and light annuli, which does not correspond to this species. The shape of the last abdominal tergite is not clear, but we attribute this to inaccuracy of the illustrations of G. Adams. The poor morphological details included in this original description led other entomologists to give the same latin name to other Lepismatidae that probably belonged to different genera. For example, in a subsequent edition of his “Systema Naturae”, Linnaeus (1767) includes additional references to drawings by other authors; one of them was in Geoffroy (1762) with the name *Forbicina plana* (Plate XX) but the drawing presented is more similar to a *Ctenolepisma* species than to a *Lepisma*. Geoffroy used the name *Forbicina* because it was used by Aldrovandi (1602) for an insect that was drawn with an aspect of silverfish (page 570). But the name *Forbicinis* (in plural) was used in this old book for a lot of insects with elongated shape (earwigs, etc.). This inaccuracy led to Olivier (1792) to present several synonyms of *Lepisma saccharinum*, only 34 years after its first description.

During the 19th century, some accurate drawings of *L. saccharinum* were published, especially those of Guérin-Meneville (1844), Nicolet (1847) and Lubbock (1873), that fit with the current concept of this species. But most records of this species did not give morphological details or the few characters indicated cannot be considered valid to distinguish between *L. saccharinum* and other silverfish species. The American origin of this species is also defended in most papers of this century. As a consequence of this, some records attributed to this taxon during the 19th century should be considered with caution because they could correspond to any *Ctenolepisma* or even to other genera of Lepismatidae. One of these doubtful records corresponds to that given by Ridley (1881) for the Canary islands. The specimens were found in hotels, so it is not unlikely that they correspond to *L. saccharinum*, but they could belong to a *Ctenolepisma* or to a *Neoasterolepisma* species, and it is difficult to conclude which one is the most probable hypothesis. The record of this species in Tunisia given by Parona (1884) is also very doubtful since no morphological support is provided, as well as details of locality or habitat. Records of this species by Nasonov (1886) from Caucasian region are also doubtful since they could not correspond to the genus *Lepisma* but, even if belonging to this genus, they could fit better with species recently described by Kaplin (2016) from this area: *Lepisma adygei* or *Lepisma xylophilum*, although both species may be not valid because they could fit within the intraspecific variability of *Lepisma saccharinum*. However, the records of *L. saccharinum* by Grassi (1887) are more reliable since morphological evidence is provided by this author for Italian insects.

B. Confusions between *Lepisma saccharinum* and *Ctenolepisma* species

One important fact to be considered is that silvery grey domestic species of silverfish different to *Lepisma saccharinum*, such as *C. longicaudatum*, *C. villosum* (as *L. targionii* in Italy) were not described or recorded in Europe and North America until the end of the 19th century and the beginning of the 20th century. This could mean that these species of *Ctenolepisma* did not arrive to these continents until this period, but the confusion is, in our opinion, the most probable hypothesis, because during the 19th century these species were unknown (undescribed) and their dorsal colour is uniformly greyish dorsally as in *L. saccharinum*. They could have occured in Europe and North America previously but not detected and identified as *L. saccharinum*. To support this, we can give several examples of species of *Ctenolepisma* that, even in the 21st century, have been identified as '*L. saccharina*', not only in citizen science platforms, but in scientific literature, in spite of the existence of taxonomic resources to distinguish between both genera (as indicated previously, after the revision of Escherich, 1905, the morphological distinction between both genera and between most species of synanthropic silverfish is possible if appropriate literature is consulted). This type of confusion is one of the most frequent in scientific literature. One example is the paper by Phillips & Gillett-Kaufman (2018) which deals with *Lepisma saccharina*, but all the photographs presented in this publication correspond to a species of the genus *Ctenolepisma*, probably *C. longicaudatum* (frontal tufts and combs of macrochaetae that are absent in Lepisma are clearly visible in their illustrations). The authors, studying a silverfish from Florida, seem to assume incorrectly that *L. saccharina* is the only species that can be identified as "silverfish" occurring in that geographic area. A second example is the publication by Joshi et al. (2020), dealing with silverfish in India, showing a photograph of a specimen of the genus *Ctenolepisma* and indicating that it corresponds to an immature stage of '*Lepisma saccharina'*. Examples of these wrong identifications are abundant.

Example: https://inaturalist.org/observations/138944687. Consulted: 10-XII-2023.

This photograph with low resolution probably corresponds to a *Ctenolepisma* species. It is identified in i-Naturalist as *Lepisma saccharinum*, but the silverfish in the photo certainly does not belong to the genus *Lepisma*.

Another relevant example is the error detected by molecular study of the silverfish sequenced by Bai et al. (2020) and identified as *Lepisma saccharinum* but resulting in being a *Ctenolepisma* species related to *C. rothschildi* and *C. longicaudatum*. This is discussed in the main text of this work. This is not an isolated example of papers by Asian authors that are not specialists in Zygentoma and do not use appropriate resources to identify them. In a work on pest control, Wang et al. (2006) refer to the effects of a natural product to control silverfish pests. They identify the silverfish as *Lepisma saccharina*, when the dead silverfish that they present in photographs (Fig. 5 of that work), probably captured in Taiwan, do not correspond to this species, but probably to a *Ctenolepisma* species.

C. Probable confusions between *Lepisma saccharinum* and *Neoasterolepisma* species.

Example: https://inaturalist.org/observations/145737298. Consulted: 10-XII-2023.

Some species of the genera *Neoasterolepisma* and *Tricholepisma* are closely related to the genus *Lepisma*. Although most species of these genera are golden yellowish and live with ants, there are *Neoasterolepisma* that seem to be associated with ants only occasionally and sometimes *Lepisma saccharinum* is found in ant colonies. This implies that records such as this of a "Lepisma-like" silverfish with ants in Gomera island (Canary Islands) could probably correspond to a native and endemic species of *Neoasterolepisma*, such as *N. myrmecobium* or an undescribed species.

D. Confusions between *Thermobia* and *Ctenolepisma* species

The typical colour pattern of dorsal scales of *Thermobia domestica* is different from the typical pattern of *Ctenolepisma lineatum* and related species, but the intraspecific variability of this character in *Thermobia* species is not well understood, and in southern Europe and southwestern North America the occurrence of native species of *Ctenolepisma* that could have similar colour patterns is very likely, and also understudied. The analysis of sequenced specimens in Fig. 1 shows that several silverfish identified as *Thermobia* are actually *C. lineatum* or related species.

Example: https://inaturalist.org/observations/100251706. Consulted: 10-XII-2023.

This is an example of a silverfish in the coast of the Gulf of California that is identified as belonging to *Thermobia* but probably corresponds to *Ctenolepisma*.

E. Confusions between *Ctenolepisma lineatum* (Fabricius, 1775) and related species

In North America, some records of *C. lineatum*, especially those of insects found in natural habitats, probably do not correspond to this species.

Example: https://www.inaturalist.org/photos/142782707. Consulted: 10-XII-2023.

This photo from California probably shows a *Ctenolepisma rubroviolaceum* or a related species. *C. rubro-violaceum* was described from California, but some authors considered this species and *C. lineatum* as synonyms, but we have studied specimens from western North America and their microscopic characters do not match with the redescription of European *C. lineatum*, a species that was described from Switzerland; see Molero Baltanás et al. (2012). *C. lineatum* occurs in North America, but as an introduced taxon that in most areas is found only in domestic habitats. Other *Ctenolepisma*-like North American species belong to undescribed native species.

Example: https://www.inaturalist.org/observations/4644864. Consulted: 10-XII-2023.

Confirming this will require a revision of native species of Lepismatidae in North America but the preliminary observations of the first author discard the possibility of all these forms corresponding to *C. lineatum*.

Moreover, a similar situation exists in *Ctenolepisma* from North Africa and the Near East, where some species occur that have been previously identified as *C. lineatum*. One of them, *C. brauni*, has been considered synonym of *C. lineatum* by Mendes (1993), but we have examined North African specimens attributed to *C. lineatum* and they are clearly different to the typical European *C. lineatum*. A preliminary study of some Canarian and Cape Verdean species that could be identified at first sight

as *C. lineatum* suggests that several undescribed species occur in the Macaronesian Region. Probably most records of *C. lineatum* in the Eastern Atlantic archipelagos are not authentic *C. lineatum*.

C. lineatum has been mentioned in some countries of the South-eastern Mediterranean and the Near East, but perhaps the records of correctly identified specimens of this species in this area correspond to domestic habitats. This is probably the case of the record published in Mendes *et al*. (2011). But specimens from this area from natural habitats re-examined by the first author of this work do not correspond to *C. lineatum* but to *C. brauni* (or perhaps to a similar undescribed species). This could be the case of specimens studied by Wygodzinsky (1952). Stach cited the species in Egypt, but a re-examination of some specimens labelled as *C. lineatum* by this author revealed that they are actually *Thermobia aegyptiaca* (Molero Baltanás et al., 2012). Maybe Stach did not dissect the specimens and examining them only with a stereomicroscope led him to this mistake.

Summarizing, records of *C. lineatum* from Europe probably do not correspond to this species if the silverfish is found in natural habitats.

Example: https://www.inaturalist.org/observations/186680926. Consulted: 10-XII-2023.

This silverfish from South Africa captured in this photograph probably belongs to the genus *Ctenolepisma* but to another species different from *C. lineatum*. The scale pattern is not characteristic of the European species.

F. Confusions between *Ctenolepisma longicaudatum* Escherich, 1905 and species with similar colour and shape of the urotergite X

Records of this species are becoming very common in the scientific literature on synanthropic silverfish, which could be interpreted as a recent expansion of this species. Although it is likely in northern European countries, the occurrence of *C. longicaudatum* in a lot of countries could date back several centuries, but the species could be misidentified with other silverfish. As indicated in the section of *Lepisma saccharinum*, the taxonomy and diagnosis of Lepismatidae species was not studied in sufficient depth until 1905, when Escherich published his 'System on Lepismatidae' and described *C. longicaudatum*. So, several records of silverfish with other names could actually correspond to this species.

Example: https://www.inaturalist.org/observations/165880640. Consulted: 10-XII-2023.

This photograph could correspond to *C. villosum* and not to *C. longicaudatum*, but confirmation requires microscopic examination of chaetotaxy. A lot of similar photographs occur in citizen science platforms that could be *C. longicaudatum*, *C. villosum*, *C. rothschildi* or *C. calvum*. Misidentifications cannot be demonstrated with the evidence of photographs only, but most identifications based on photographs of these species can be considered as doubtful. Differences between these species are not conveniently described in most works, especially if not presented by specialists.

Consulting the literature on synanthropic silverfish that could disorient non-experts, we detected that in the identification key presented in Aak et al. (2019) we can read for *C. longicaudatum*: "*Three bristle combs, of which two dorsally, on each side of the abdominal segments II – VI*". This statement could lead to misinterpretation, because, if we take into account ventral bristle combs, the description is only valid for abdominal segment II: there are four bristle combs on each side of the abdominal segments III–VI. The comb that, according to this description is not placed dorsally, could be interpreted as ventral and not as the infralateral comb inserted on the part of the abdominal tergites

that bend laterally. In fact, a correct description of this chaetotaxy should mention that in *C. longicaudatum* there are three bristle combs on each side of the abdominal tergites II–VI, of which two are dorsal and one is infralateral, and there is one additional bristle comb on each side of abdominal sternites III–VIII.

The specific identity of *C. longicaudatum* will be even more difficult because we have identified (with genetic tools, see Fig. 1) more than one lineage of silverfish that have been identified with this name. This probably means that assessing probable morphological differences between these lineages will require microscopic investigation.

G. Errors related to *Ctenolepisma calvum* (Ritter, 1910)

In the main text of this work, after the redescription of this species, we have discussed the mistakes made by Ritter (1910) when describing this species as a new genus (*Peliolepisma*) and Wygodzinsky's obstinacy in maintaining this genus as different to *Ctenolepisma* only on the basis of bearing only one macrochaeta on each side of the posterior margin of the thoracic nota. We have demonstrated that these posterior combs in some specimens of *C. calvum* can bear more than one macrochaetae and that this low number is similar to other *Ctenolepisma* species. This does not mean that *C. calvum* belongs to the same clade as other lineages of *Ctenolepisma* because this genus requires revision and probably splitting into several genera (and perhaps the name *Peliolepisma* could be resurrected), but not on the basis of this unique character and even less on the basis of characters defined by Ritter, that are incorrect. In this section we focus mainly on the morphological mistakes or doubtful records presented by non-specialist authors in recent years referring to this species.

We have also discussed in the main text of this work the inaccuracies of the descriptions, identification keys or comparative tables recently presented by some non-specialist authors. In this supplementary document we present additional errors related to the recent records of this species in Europe.

The first record of this species in Europe (Erlacher, 2017) was presented in a webpage and indicates the occurrence of this species in a house and a Museum in Chemnitz (Germany). In our opinion, the reliability of this record is based on the following statement that was indicated in that webpage: "The Chemnitz specimens were identified together with a colleague from the University of Lisbon, who has been intensively studying this group of animals for decades." Although the name of this Portuguese colleague is not mentioned, we think that he can be no other than Luis Mendes. No morphological details are explained in this report, but we can give reliability to subsequent identification of S. Erlacher considering that they were helped by an expert.

In another webpage, Hegyessy (2021) documents with photographs. the occurrence of *C. calvum* in a locality of Hungary, without indicating expressly the habitat where the insect was found. The morphological details that the author uses for the description are only useful to distinguish *Ctenolepisma* species from *Lepisma saccharinum*. The photographs show that the insect presented corresponds very likely to *C. calvum*, but there is a lack of expertise in the morphological description used by the author. He concedes (in Hungarian language) that "There are many similar, poorly known species in this genus, so it is also possible that it is a different relative". Nevertheless, we concede that the occurrence of *C. calvum* in Hungary is very probable. However, the main mistake presented by Hegyessy consists of indicating that the species has been found in Portugal (Lisbon), probably misinterpreting Erlacher's comments about the origin of the person who helped him with the

identification. Some other authors subsequently include the occurrence of the species in Portugal on the basis of this mistake (Querner et al., 2022).

Mattson (2018) reports the occurrence of *C. calvum* in Norway, but his identifications should be taken with caution because the details presented to justify the specific identity of this silverfish are poor, as well as subsequent details given by Aak et al. (2019, 2021). Moreover, Mattson presents in the photo of page 14 of his work (in Norwegian language) a specimen of *Lepisma saccharinum* (on the left) together with a probable *C. calvum* (on the right) and he writes that the *Lepisma saccharinum* is a *C. longicaudatum* (clear misidentification). If the statement of Aak indicating that *C. calvum* and *C. longicaudatum* can co-occur is based on this photo, it is not well founded. Nevertheless, considering that Querner et al (2022) indicate that Erlacher has specimens from Norway in his database (unfortunately unpublished), we consider records from Norway as reliable. These identifications should be taken with caution.

Querner et al. (2022) presented several records of this species mixing academic works by non-experts and records based on photographs of i-Naturalist. Only the records included in Table S2 (Supplemantary material) that are based on observations by S. Erlacher or P. Querner can be considered reasonably reliable; the problem is that concrete details of the localities are not given. The authors present a list of museums sampled in supplementary document S1, but they do not indicate in which of them was the species collected. Specimens from Poland were used for DNA analysis and these represents the most reliable records since they match with the Spanish *C. calvum* identified and sequenced by us for the present work. As the records from Teneriffe (Spain), Russia, Croatia, Spain, Kosovo, Slovenia, Ukraine, Portugal, and Slovakia, are based only on i-Naturalist, they are considered doubtful. In the map of *C. calvum* they can be considered as doubtful with a question mark, except for the record from Slovakia, that has been subsequently published with enough support (Bednár et al., 2023). It must be said that the preliminary draft of this latter work on Slovakian silverfish had many errors that were corrected thanks to the fact that the editors of the journal where it was published were successful in finding an expert reviewer. This did not happen in the case of the editor of the journal where the work of Querner et al. (2022) was published. This example leads us to highlight the importance of taxonomists who are in danger of extinction.

As an example of the chaos that could be created the acceptance of i-Naturalist records without verification of real experts, we are going to assess the records in Spain and Portugal of *C. calvum* in this platform that are presented up until November 2022:

SPAIN

Tenerife (**Los Gigantes Cliffs**). https://spain.inaturalist.org/observations/51710311 (Consulted: 10-XII-2023). This photo could correspond to *C. calvum* or not. It would be interesting to know if it was located inside a building or in a natural habitat. If the latter, as it seems to be according to the coordinates given on i-Naturalist, it is very likely that the species photographed is a *Ctenolepisma longicaudatum*. Specimens of this species found in the Canary Islands and Cape Verde have that light appearance. In any case, it is likely that this photograph does not correspond to *C. calvum*.

Fuerteventura Island. https://spain.inaturalist.org/observations/105356412 (Consulted: 10-XII-2023). This photo corresponds to an insect in natural habitat. Most likely it belongs to the genus *Ctenolepisma* but there is very little probability that it corresponds to *Ctenolepisma calvum*, but rather to a native species, of which there are probably many to be described.

Even it could belong to the subfamily Lepismatinae. In fact, from that area one of us has found an undescribed species of *Neoasterolepisma*.

Albacete. https://spain.inaturalist.org/observations/102271519 (Consulted: 10-XII-2023). It could be *C. calvum*, but it is not sure (a juvenile of *C. villosum* or *C. longicaudatum* are options that cannot be discarded without microscopic examination).

Dos Hermanas (**Sevilla**). https://spain.inaturalist.org/observations/13366258 (Consulted: 10-XII-2023). Almost certainly this photo does not correspond to *C. calvum*. The photograph is of poor quality, but it is most likely a *C. longicaudatum* with light gray scales.

Reus (**Tarragona**). https://spain.inaturalist.org/observations/144303780 (Consulted: 10-XII-2023). It could be *C. calvum*, but it could also correspond to *C. villosum*, light specimens that we have also found in Córdoba of this species (see photograph in the main document).

Barcelona. https://spain.inaturalist.org/observations/18101991 (Consulted: 10-XII-2023). Very deteriorated specimen, lacking scales. It could correspond to any *Ctenolepisma* species.

Lloret de Mar (**Girona**). https://spain.inaturalist.org/observations/169146575 (Consulted: 10-XII-2023). Very low-quality photo, it could correspond to an adult of *C. calvum*, to an adult of *C. villosum* or to a scaleless or young specimen of any species of *Ctenolepisma*.

Madrid. https://spain.inaturalist.org/observations/188829732 (Consulted: 10-XII-2023). As the record from Albacete, it could be *C. calvum* but it could also correspond to *C. villosum*, light specimens that we have also found belonging to this species.

PORTUGAL

Vila Real de Santo Antonio. https://spain.inaturalist.org/observations/96463382 (Consulted: 10-XII-2023). This could be *C. calvum*, but the insect occupies a very small part of the photo, it has low resolution and it could correspond to a young specimen of *C. villosum* or *C. longicaudatum*.

Albufeira. https://spain.inaturalist.org/observations/178703772 (Consulted: 10-XII-2023). The photo shows a larva of an holometabolous insect that does not even belong to the order Zygentoma, absurdly identified as *Ctenolepisma calvum*.

Therefore, geographic distribution models of Lepismatidae based on these types of records should be interpreted with great skepticism since their basis is fundamentally dubious or evidently erroneous. The authors of this work would need to spend a very long time to detect and assess all erroneous or doubtful records, but we believe that this is only worthwhile if this work were sufficiently recognized or compensated. This is not the case since taxonomists are considered in recent decades as second-class scientists. It seems that it will take many more blatant errors before the scientific community becomes aware of the loss that is being incurred by relinquishing the sufficiently compensated expert work of taxonomic specialists.

With this document we simply criticize bad practices of spreading doubtful records and/or evident errors in web platforms. Perhaps for other groups of common animals photographs are enough for distinguishing species, but this is not the case for the Zygentoma, a group that in most countries of temperate and tropical regions requires further study to complete the knowledge of their fauna.

H. Comparison between descriptions

Table S4-1. Comparison between characters described for *Ctenolepisma targionii* (Grassi & Rovelli) by Stach (1935) on Egyptian species, those described for *Ctenolepisma villosum* (Fabricius, 1775) by Yossi (1939) on Japanese specimens, and those for specimens previously identified as *Ctenolepisma targionii* recorded in Italy, Spain and Iran and studied by Molero-Baltanás et al. (2000), Molero-Baltanás et al. (1996), Molero Baltanás et al. (2015) and Molero-Baltanás et al. (2018). The original text by Stach (1935) was in German and the text by Yosii (1939) was in Japanese and in German. The description of specimens studied by Molero-Baltanás et al. has been written directly in English. Translation from German to English has been done by native English speakers with a high knowledge of German. It seems that Yosii, when translating his Japanese text to German, followed the same description style used by Stach, so some parts of the descriptions of Stach of *C. targionii* (in German) and of Yosii of *C. villosum* are very coincident (marked with blue). Characters where differences could correspond to intraspecific variability are marked with yellow. One evident mistake is marked with green.

***Ctenolepisma targionii* description by Stach (1935) on Egyptian specimens**	***Ctenolepisma villosum* described by Yosii (1939) on Japanese specimens**	***Ctenolepisma targionii* studied by Molero-Baltanás *et al.* (1996; 2000; 2015, 2018) on Spanish, Italian and Iranian specimens**
The colour of the animal in alcohol after the scales are removed is whitish	The animal is greyish white, with silver reflections in life and yellowish white in the specimen that has been preserved in alcohol, and whose scales are denuded.	Epidermic pigment absent or, if present, very scarce, light yellowish over all the body.
The whole body of the animal is covered with scales, which are brownish on the back, colourless under the body. The scales are large, with numerous straight, parallel rays. The head is decorated with a few radiating bushes of feathered bristles.	The whole body is covered with scales, which are blackish on the back, colourless under the body. The scales are large, with numerous straight, parallel rays. The head is covered with radiating tufts. In the apex with two bifurcate bristles.	All the body is covered by scales, with the exception of appendages (although the scapus of the antenna, the coxae and the femora are covered by scales). Dorsal scales usually uniform dark greyish, frequently with silvery iridescence, sometimes almost blackish and sometimes light greyish or even whitish hyaline (this is more frequent in young specimens). Ventral scales hyaline or whitish.
The body length of the largest specimen available, from the anterior margin to the articulation with the medial filament was 10 mm.	The body length of the largest specimen available, was 10 mm;	Maximum body length: 8.7 mm in males and 9.6 mm in females Maximum thorax length observed: 3 mm in both sexes. Thorax width: up to 2.1 mm.
The length of the antennae 6 mm; the cerci 5–6 mm. Antennae are slightly longer than half the length of the body covered with numerous bristles, long trichobothria and small blister-like structures.	the length of the antennae 6 mm; the cerci 4.5 mm and the terminal filament 7 mm.	The maximum preserved length of an antenna is 6.5 mm, although it is very likely that they can be a little longer since its tip is easily broken. They are always shorter than body length, about 70–75% of this length. The maximum preserved length of a terminal filament is 5.2 mm, also broken at its end. It seems that they are a little shorter than antennae.

Table S4-1 (continued)

***Ctenolepisma targionii* description by Stach (1935) on Egyptian specimens**	***Ctenolepisma villosum* described by Yosii (1939) on Japanese specimens**	***Ctenolepisma targionii* studied by Molero-Baltanás *et al*. (1996; 2000; 2015, 2018) on Spanish, Italian and Iranian specimens**
The ultimate article of the labial palp in adult individuals of both sexes about as long as wide and equipped with 5 sensory papillae in a transverse row close to the upper margin.	The ultimate article of the labial palp is in adult individuals almost as long as wide and equipped with 3–4 sensory papillae in a transverse row	The apical article of labial palp has usually four papillae arranged in a single row. A fifth papilla, often smaller than the others, is visible in some specimens.
The penultimate article is thickened club-shaped in its distal half and as long as the last but about ⅓ narrower.	The penultimate article is as long as the apical one.	Apical article as long as the penultimate article of the labial palp.
On the lateral margins of the thoracic tergites there are transverse oriented marginal combs composed of 3–5 strong, feathered bristles located close to each other and curving backwards, in addition to the isolated bristles. Some of these strong bristles are about as long as half the length of the tergites in this species. In addition, on either side of the hind margin, there are dorsal combs composed of 3–7 bristles.	On the lateral margins of the thoracic tergites there are erect setae arranged in marginal combs composed of 4 macrochaetae. Some of these setae are about 2/3 of the length of the tergite. The anterior margin of the pronotum has a fringe of strong setae. The posterior margin has one comb on each side composed of 4–6 macrochaetae.	Pronotum with 8-9 pairs of lateral combs on each side, mesonotum with 11-13 lateral combs and metanotum with 9-11, composed of 2–7, 2–5 and 1–4 macrochaetae each. Hind margin of nota with 1+1 posterior combs, usually of 4–5 macrochaetae.
The tergites of abdominal segments I–VIII are equipped with bristle combs. Each side of the tergite of abdominal segment I carries only one bristle comb, which is completely lateral, already on the enveloping part of the tergite.	The tergites of abdominal segments I–VIII are equipped with bristle combs. Each side of the tergite of abdominal segment I carries only one bristle comb.	Urotergites I–VIII with combs of macrochaetae. Urotergite I with 1+1 combs in infralateral position.
On each side of the tergites of abdominal segments II–V there are 3 bristle combs, namely a dorsal outer, a dorsal inner and a lateral on the enveloping part of the tergite	On each side of the tergites of abdominal segments II–V there are 3 bristle combs, namely a dorsal outer, a dorsal inner and a lateral on the enveloping part of the tergite	Urotergites II–V with 3+3 combs of macrochaetae (with the usual position of those Ctenolepismatinae bearing this arrangement: one infralateral (comb A), one lateral (comb B) and one submedian (C).
On either side of the tergites of abdominal segments VI–VIII there are 2 combs, a dorsal inner and a lateral on the enveloping part of the tergite. On the tergite of abdominal segment IX there are no bristle combs.	On either side of the tergites of abdominal segments VI–VIII there are 2 combs, a dorsal inner and a lateral on the enveloping part of the tergite. On the tergite of abdominal segment IX there are no bristle combs.	Urotergites VI–VIII with 2+2 combs of macrochaetae (in infralateral and lateral position). Urotergite X bare.

Table S4-1 (continued)

Ctenolepisma targionii **description by Stach (1935) on Egyptian specimens**	***Ctenolepisma villosum*** **described by Yosii (1939) on Japanese specimens**	***Ctenolepisma targionii*** **studied by Molero-Baltanás** ***et al*****. (1996; 2000; 2015, 2018) on Spanish, Italian and Iranian specimens**
The dorsal outer and inner combs consist of 5–7 strong bristles. The lateral combs on tergites I-V are composed of 5–7, on VI-VIII of 9–10 and on IX of 15 strong bristles (1)	The dorsal outer and inner combs consist of 4–5 strong bristles. The lateral combs on tergites I-VIII are composed of 7–8 bristles.	Combs B and C with 4–6 macrochaetae, exceptionally 7. Combs A (infralateral) usually with more than 6 macrochaetae (the range is 5–9)
Tergite X is short, somewhat trapezoidal, with rounded corners, only very slightly concave on the posterior margin.	Tergite X is short, somewhat trapezoidal, with rounded corners, only very slightly concave on the posterior margin	Urotergite X trapezoidal with rounded posterior corners; its ratio length/width is 0.32-0.45, and its hind margin can be almost straight or clearly concave
On the side margins of the tergite there are short and long bristles in two approximate rows. The two bristle rows extend over the rounded posterior corners, however a small stretch of the hind margin of the tergite is free of bristles. On each side close to the rounded corner there is a bristle comb, which is composed of 7–8 strong, feathered bristles.	On each side close to the rounded corner there is a bristle comb, which is composed of 6–9 strong, feathered bristles	The 1+1 combs of the urotergite X have 7–9 macrochaetae each.
On each side of the sternites of abdominal segments III-VIII (and not I-VIII, as given by ESCHERICH) there is a lateral comb on the hind margin, which is composed of 12 in the female and 11–15 bristles in the male.	On each side of the sternites of abdominal segments III-VIII there is a lateral comb on the hind margin, which is composed of 6–7 bristles.	Urosternites III-VIII with 1+1 lateral combs consisting of 7–12 macrochaetae.
In the midline of the sternites of abdominal segments II-VI (and not I-VI, as given by Escherich), there is an unpaired medial comb, composed of 10 bristles on sternite II and 6–8 on the remainder.	In the midline of the sternites of abdominal segments II-VI (and not I-VI, as given by Escherich), there is an unpaired medial comb, composed of 6 bristles	Urosternites II-VI with a median comb of 6–12 macrochaetae.
Two pairs of stylets are present in the females, namely on abdominal segments VIII and IX; in the males only one pair is present. They are fairly long, about ¼ shorter than the subcoxa and with numerous fairly long, smooth bristles, as well as irregular longitudinal rows of strong, feathered bristles. At the apex of the styli there is a somewhat stronger feathered bristle.	Two pairs of stylets are present in the females, namely on abdominal segments VIII and IX; in the males only one pair is present. They are quite long and covered with numerous smooth bristles. At the apex of the styli there is a somewhat stronger smooth bristle, slightly more brownish in colour.	Males with only one pair of styli associated with the ninth abdominal segment. Females always with two pairs of abdominal styli. The apex of the styli bears a spine that is apparently smooth with low magnification but slightly feathered if observed with more detail.

(1) **Marked with green character**: It is an obvious mistake of Stach, because the urotergite IX is bare, as he previously states.

Table S4-1. Continuation of previous table.

***Ctenolepisma targionii* description by Stach (1935) on Egyptian specimens**	***Ctenolepisma villosum* described by Yosii (1939) on Japanese specimens**	***Ctenolepisma targionii* studied by Molero-Baltanás *et al*. (1996; 2000; 2015, 2018) on Spanish, Italian and Iranian specimens**
The coxa of abdominal segment IX in the female are covered with bristles on the medial margin from above to apex of the median process, the bristles being short and smooth above and on the median process long and fringed . In the males the median process is covered in bristles for a much shorter distance on the margin. The outer process is equipped with only a few, namely about 5 longer, stronger bristles and 5–7 shorter, weaker bristles, in both sexes.	In the males the median process is much shorter and thicker than in the females. The outer process is in both sexes with 4–5 weaker bristles.	Coxite IX with its inner process about 1.1–1.3 longer than wide at its base in males and 1.5–2 times longer than wide in females. The inner process is about 4–5 times longer than the outer process in both sexes (close to 4 in males). These metrics are lower in juveniles.
The median process is clearly longer than the outer process, however not especially long, as its apex only reaches to about ⅓ the length of the stylus.	The median process is clearly longer than the outer process, its apex only reaches to about half the length of the styli.	In both sexes the styli IX are 2 or more times longer than the inner process of the coxite IX (up to 2.5 times). The apex of the coxite usually reaches half the length of the styli in females and about 1/3 the length of the styli in males.
Penis thin, moderately long, of two articles. Parameres could not be noticed.	Penis cylindric, broad, bilobed, and with a shallow median indentation on posterior margin; projecting between both gonocoxites of IX abdominal segment. It lacks parameres entirely	Penis as usual in Lepismatidae. Without parameres, as in other Ctenolepismatinae.
Ovipositor long, extending beyond the apex of the styli stretched backwards by the whole length of the styli. The ventral (anterior) part of the ovipositor connected to abdominal segment VIII, is composed of 35 articles. The dorsal (posterior) part of the ovipositor connected with abdominal segment IX consists of 33–35 divisions.	Ovipositor long, extending beyond the apex of the styli stretched backwards by half the length of the stylets. The ventral (anterior) part of the ovipositor connected to abdominal segment VIII, is composed of 38 articles. The dorsal (posterior) part of the ovipositor connected with the IX abdominal segment consists of about 40 divisions.	In adult females, the ovipositor has 40 or more than 40 divisions and can surpass the tip of the inner process of the coxite by more than 3 times its length. These metrics can be lower in young females (shorter ovipositor, with lower number of divisions).

Ctenolepisma targionii **description by Stach (1935) on Egyptian specimens**	***Ctenolepisma villosum* described by Yosii (1939) on Japanese specimens**	***Ctenolepisma targionii* studied by Molero-Baltanás *et al*. (1996; 2000; 2015, 2018) on Spanish, Italian and Iranian specimens**
The last, distal division has a moderately long bristle near the apex and higher about 15 short bristles. On the penultimate division and about 8 following divisions there are 5 short bristles in two transverse rows, namely in the lower row 2, in the upper 3 bristles. From the 11th division there are 3 bristles in the lower row, 1 short bristle in the upper and on further articles in the lower row 2, in the upper 1 bristle. Starting from the 18th division these bristles become very small. In gonapophyses IX, the last distal division carries 3 moderately long bristles in a row near its apex; somewhat higher there are about 10 short sensory pegs that are not apically thickened to a club-shaped but rather truncated sensory pegs and further over 20 short bristles. On the penultimate division stand 9 – 10 short bristles arranged in two irregular transverse rows, on the following 3rd and 4th divisions 9, on the 5th and 6th 7, on the 7th to the 9th 5, on the 10th and 11th 4, on 12th 2 in the lower row and 1 bristle in the upper row and from the 14th division on there are only 2 bristles remaining in the lower row of each division, which become very tiny on succeeding divisions.	The last, distal division has a long bristle near the tip and about 6 short bristles basally. On the other divisions there are 4 short bristles each in transverse rows, namely 2 bristles each in lower and upper row.<br>The last distal limb bears near its tip 3 long bristles, each with a stalk in the base; more basally there are about 7 short bristles and 6-8 sensory rods, which are blunted at their tip. On other limbs there are 5 short bristles in two irregular transverse rows.	The chaetotaxy of the ovipositor has not been studied with detail in Spanish specimens since it has a considerable range of variation and it seems that it is not taxonomically relevant.
Locality:<br>Hill in the desert about 4–6 m north of Heluan, under stones; desert near Wadi farm about 4 km NE Heluan, under the stones. The sample identified by Wahlgren as *Ctenolepisma targionii* was collected in Heluan, under stones.<br>*Ctenolepisma targionii* (Grassi & Rovelli) Escherich appears to be a species endemic to the countries bordering the Mediterranean; according to Grassi & Rovelli it enters into human dwellings. It has so far been recorded from Sardinia, the Aeolian Islands, Sicily (Catania), the Apeninne Peninsular (Florence, Rome), from Spain (Flix in Tarragona province) and from Egypt (Heluan). I possess also an example from Albania (Tirana).	Locality: Hokkaido, Honshu, Sikoku, Kiusiu, Flores, India.<br>In houses, eats paper and …	This is a strict domestic or peridomestic species in the Iberian and the Italian Peninsulas. In the south of Spain one specimen was collected in a crop, but near a building. In Iran one specimen was collected in a cave.

References

Aak A, Rukke BA, Ottesen PS. 2019 Long-tailed silverfish (*Ctenolepisma longicaudata*) - biology and control. Norwegian Institute of Public Health Report. Norwegian Institute of Public Health, pp. 43.

Aak A, Hage M, Magerøy Ø, Byrkjeland R, Lindstedt HH, Ottesen P, Rukke BA. 2021. Introduction, dispersal, establishment and societal impact of the long-tailed silverfish *Ctenolepisma longicaudata* (Escherich, 1905) in Norway. BioInvasions Record. 10(2):483-498.

Adams G. 1771. *Micrographia illustrata*: or The microscope explained. Likewise, a natural history of aerial, terrestrial, and aquatic animals, considered as microscopic objects. London: Printed for the author. pp. 325 (71 Pl) pp.

Aldrovandi U. 1602. De animalibus insectislibri septem, cum singulorum iconibus ad viuum expressis. Autore Vlysse Aldrouando in almo Gymnasio Bonon. Cum indice copiosissimo. Bononiae: apud Ioan. Bologna: Bapt. Bellagambam. 12, pp. 45.

Bai Y, Chen J, Li G, Wang H, Luo J, Li C. (2020). Complete mitocondrial genome of the common silverfish *Lepisma saccharina* (Insecta: Zygentoma: Lepismatidae). Mitochondrial DNA B. 5(2):1552–1553.

Bednár F, Hemala V, Čejka T. 2023. First records of two new silverfish species (*Ctenolepisma longicaudatum* and *Ctenolepisma calvum*) in Slovakia, with checklist and identification key of Slovak Zygentoma. Biologia.

Brown P. 1756. The civil and natural history of Jamaica. London: Gray's-Inn. Printed for the author. pp. 503 (49 Pl).

Erlacher S. 2017. Aliens Unter Uns–Eine Begegnung Mit der Sechsten Art. Stadt Chemnitz. Pressemitteilung der Stadt Chemnitz vom. 4 April 2017. Available online: https://www.chemnitz.de/chemnitz/de/aktuell/presse/pressemitteilungen/2017/203.html Acessed on 21 October 2023.

Escherich K. 1905. Das System der Lepismatiden. Zoologica, N°43. Stuttgart: Universität Straßburg. pp. 164.

Geoffroy M. 1762. Histoire abregée des Insectes (Vol. 2). Paris. p. 613.

Grassi B, Rovelli G. 1889. Tavola analitica dei Tisanura da noi finora riscontrati. Bollettino della Società Entomologica Italiana. 21:3–8.

Guérin-Méneville FE. 1844. Iconographie du Règne animal de G. Cuvier 7, Texte explicatif [Lepisma]. Vol. 3:9-20.

Hegyessy G. 2021. Ezüstérmes Rovar – Ezüstös Ősrovar. Available online: http://kazinczyferencmuzeum.hu/2021/01/04/ezustermes-rovar-ezustos-osrovar/ (In Hungarian). Accessed on 21 October 2023.

Joshi MJ, Raj VP, Solanki CB, Vaishalikumari VB. 2020. Silverfish (*Lepisma saccharina*): An Overview and their Management. Agriculture & Foods: e-Newsletter. 2(3):490-493.

Kaplin VG. 2016. New Species of the Bristletail Families Ateluridae and Lepismatidae (Zygentoma) from Abkhazia and Adygea. Zoologicheskii Zhurnal, 96(7):885–898. [Original Russian Text: Kaplin VG. 2016. Zoologicheskii Zhurnal. 95(8): 931–944.]

Linnaeus C. 1758. Systema Naturae (Ed. 10; Vol. 1). Holmiae.

Linnaeus C. 1767. Linnaeus, C. (1758-1767). Systema Naturae (Vol 1(2)). 12. Holmiae.

Lubbock J. 1873. Monograph of the Collembola and Thysanura. Ray Soc. pp. 276.

Mattson J. 2018. Kre i Norge ved to av dem. Insekt-Nytt. 43:13–18. [In Norwegian].

Mendes LF. 1993. New data on the thysanurans (Microcoryphia and Zygentoma: Insecta) from Northern Africa and from the Near East. Garcia de Orta, Séries Zoologia, Lisboa. 18(1-2):79–93.

Mendes LF, Molero-Baltanás R, de Roca CB, Gaju-Ricart M. 2011. New data and new species of Microcoryphia and Zygentoma (Insecta) from Israel. Ann. Soc. entomol. France. 47(3-4):384-393.

Molero R. 1995. Estudio taxonómico de los Zygentoma de España (Insecta: Apterygota). Unpublished Thesis. Universidad de Córdoba, Spain. 580 pp.

Molero R, Tahami MS, Gaju M, Sadeghi S. 2018. A survey of basal insects (Microcoryphia and Zygentoma) from subterranean environments of Iran, with description of three new species. ZooKeys. 806:17-46.

Molero-Baltanás R, Fanciulli PP, Frati F, Carapelli A, Gaju-Ricart M. 2000. New data on the Zygentoma (Insecta, Apterygota) from Italy. Pedobiologia. 44(3-4):320-332.

Molero-Baltanás R, Gaju-Ricart M, Bach de Roca C. 1996. Los lepismátidos antropófilos de España. Real Sociedad Española de Historia Natural. 125:178-181.

Molero Baltanás R, Gaju Ricart M, Bach de Roca C. 2012. New data for a revision of the genus *Ctenolepisma* (Zygentoma: Lepismatidae): redescription of *Ctenolepisma lineata* and new status for *Ctenolepisma nicoletii*. Ann. Soc. Entomol. Fr. 48(1-2):66-80.

Molero Baltanás R, Gaju Ricart M, Bach de Roca C. 2015. Actualización del conocimiento del género *Ctenolepisma* Escherich, 1905 (Zygentoma, Lepismatidae) en España peninsular y Baleares. *Boletín de la Asociación Española de Entomología*. 39(3):365-390.

Molero-Baltanás R, Smith GB, Mendes LF, Gaju-Ricart M, Bach de Roca C. 2016. Case 3704 *Lepisma* Linnaeus, 1758 (Insecta, Zygentoma, Lepismatidae): proposed reversal of Direction 71 (1957) regarding the gender of the name. Bull. Zool. Nomencl. 73(1):7–16.

Nasonov NV. 1886. Thysanura Kavkaza. Izvěst. imp. Obšc. Ljubit. Jestestvozn. 50, Trudy zool. Otděl 1:307-307.

Nicolet H. 1847. Essai sur une classification des insectes aptères de l'ordre des Thysanoures. Ann. Soc. entomol. France. Sec. 2. 5:335-395.

Olivier AG. 1792. Insectes. In: Encydopédie méthodique, Histoire naturelle. Paris. pp. 506-508.

Parona. 1884. Materiali per lo studio della fauna tunisiana raccolti da G.e L. Doria. IV. Sopra alcune Collembola e Thysanura di Tunisi. Ann. Mus. Stor. natur. Genova, Sec. 2. 1:425-438.

Phillips E, Gillett-Kaufman JL. 2018. Silverfish *Lepisma saccharina* Linnaeus (Insecta: Zygentoma: Lepismatidae). EENY705/IN1211, 5/2018. EDIS, 2018(4).

Querner P, Szucsich N, Landsberger B, Erlacher S, Trebicki L, Grabowski M, Brimblecombe P. 2022. Identification and Spread of the Ghost Silverfish (*Ctenolepisma calvum*) among Museums and Homes in Europe. Insects. 13:855.

Riddley HN. 1881. Notes on *Thysanura* collected in the Canaries and Madeira. Entomol. Monthly Mag. 18:14.

Ritter W. 1910. Neue Thysanuren and Collembolen aus Ceylon and Bombay, gesammelt von Dr. Uzel. Ann. naturh. Hofmus. 24:379-398.

Stach J. 1935. Die Lepismatiden-Fauna Ägyptens. Annales Musei Zoologici Polonici. 11(4):27–111.

Wang S-Y, Lai W-C, Chu F-H, Lin C-T, Shen S-Y, Chang S-T. 2006. Essential oil from the leaves of *Cryptomeria japonica* acts as a silverfish (*Lepisma saccharina*) repellent and insecticide. J Wood Sci. 52:522–526.

Wygodzinsky P. 1952. Results of the Zoological Scientific Expedition of the National Museum in Praha to Turkey. 5. Thysanura (Machilidae and Lepismatidae). Sborník entomol. Odd. nár. Mus. Praha. 26(377):1-9.

Yosii R. 1939. Wiederbeschreibung eines Bücherwurms, *Ctenolepisma villosa*. Zoo. Mag. Tokyo. 51:677–682.
